# CRISPR-Cas9-mediated knockup of *OsDREB1C* enhances rice yield without compromising grain quality

**DOI:** 10.1016/j.xplc.2025.101433

**Published:** 2025-06-27

**Authors:** Yanmin Luo, Xiaodeng Zhan, Yingxin Zhang, Beifang Wang, Guanqi Wang, Yue Zhang, Guzi Li, Qunen Liu, Xihong Shen, Daibo Chen, Yongbo Hong, Weixun Wu, Guoyou Ye, Shihua Cheng, Gang Pan, Liyong Cao

**Affiliations:** 1 State Key Laboratory of Rice Biology and Breeding, China National Rice Research Institute, Hangzhou 311400, China; 2 Northern Rice Research Center of Baoqing, Shuangyashan 155600, China; 3 National Nanfan Research Institute (Sanya), Chinese Academy of Agricultural Sciences, Sanya 572025, China; 4 Key Laboratory of Northern Japonica Rice Research in Heilongjiang Province, Baoqing Northern Rice Research Center, Baoqing, Heilongjiang 155600, China; 5 International Rice Research Institute, Los Banos, Laguna 4031, Philippines; 6 Department of Agronomy, Zhejiang University, Hangzhou 310058, China

## Abstract

This study presents a CRISPR-Cas9-based strategy for engineering structural variations in the *OsDREB1C* gene in rice, leading to a yield increase of over 20% without compromising grain quality. The resulting homozygous plants are transgene-free, highlighting the potential of this approach for precise and effective crop improvement.

Dear Editor,

CRISPR-Cas9 genome editing has revolutionized plant breeding by enabling precise genetic modifications in staple crops such as rice and wheat (Zhu et al., 2020). Through targeted gene knockout or knockdown, this technology has demonstrated remarkable success in improving yield potential, stress resistance, and nutritional quality (Zhang et al., 2017, 2021; Li et al., 2024). In rice breeding, CRISPR-Cas9 has been successfully used to induce genomic structural variation (SV) and enhance pesticide resistance (Lu et al., 2021). However, inversions, an often-overlooked type of SV, remain rarely reported despite their potential for crop improvement (Hu et al., 2024). Inversions occur when CRISPR-Cas9 induces double-strand breaks at the promoter and coding regions of two oppositely oriented genes on the same chromosome. This inversion-style rearrangement can swap the genes’ promoters, altering their expression (Lu et al., 2021), and potentially enhancing desirable phenotypes such as yield.

In this study, we report the CRISPR-Cas9 engineering of SVs in *OsDREB1C* (*Os06g0127100*), a rice yield-enhancing gene, by increasing grain number and weight (Wei et al., 2022). We hypothesized that *OsDREB1C* could be repositioned under a promoter with high transcript activity following CRISPR-Cas9-mediated inversion. Database analyses (The Rice Annotation Project Database, Rice Genome Annotation Project, and CRISPR- Applicable Functional Redundancy Inspector in Rice) identified the histone H1 gene *OsH1* (*Os06g0130800*) as an ideal candidate (Supplemental Figure 1A and 1B; Supplemental Table 1), located 209.11 kb from *OsDREB1C* (Figure 1A) with opposite transcriptional orientation. Functional redundancy of *OsH1* was confirmed by two knockout mutants (*osh1-1* and *osh1-2*) that showed no agronomic defects (Supplemental Figure 2; Supplemental Table 2), consistent with histone H1’s conserved role in epigenetic regulation across species (Wierzbicki and Jerzmanowski, 2005; Rea et al., 2012). We then determined the expression patterns of both *OsH1* and *OsDREB1C* in the *japonica* rice cultivar Jingeng818 during the booting stage. Both genes were ubiquitously expressed, with *OsH1* exhibiting remarkably higher levels of gene expression than *OsDREB1C* (Supplemental Figure 1C). Given the opposite directions of *OsDREB1C* and *OsH1* on chromosome 6, we employed CRISPR-Cas9 to edit their 5′ untranslated regions (UTRs) using single guide RNAs (sgRNAs). Our goal was to invert the genomic segment between the two genes’ cutting sites and swap their promoters. A vector containing two sgRNAs was first expressed in rice protoplasts, as verified by PCR using the specific primer pairs OsDREB1C-F2/OsH1-F1 and OsDREB1C-R2/OsH1-R1, respectively (Supplemental Table 8). As expected, inversion of the genomic segment containing *OsDREB1C-OsH1* was successfully achieved (Supplemental Figure 3A). The sgRNA-expressing vector and its empty vector (EV) were next introduced into rice calli of the Jingeng818 background via *Agrobacterium*-mediated transformation. From 8.85 g of transformed calli, we obtained 165 hygromycin-resistant calli. Inversion events were validated in ten calli, showing five distinct genotypes on the right side (Promoter_OsH1_::OsDREB1C). This represented a positive callus percentage of 6.06% (10 of 165) (Supplemental Tables 3 and 4).

**Figure 1:**
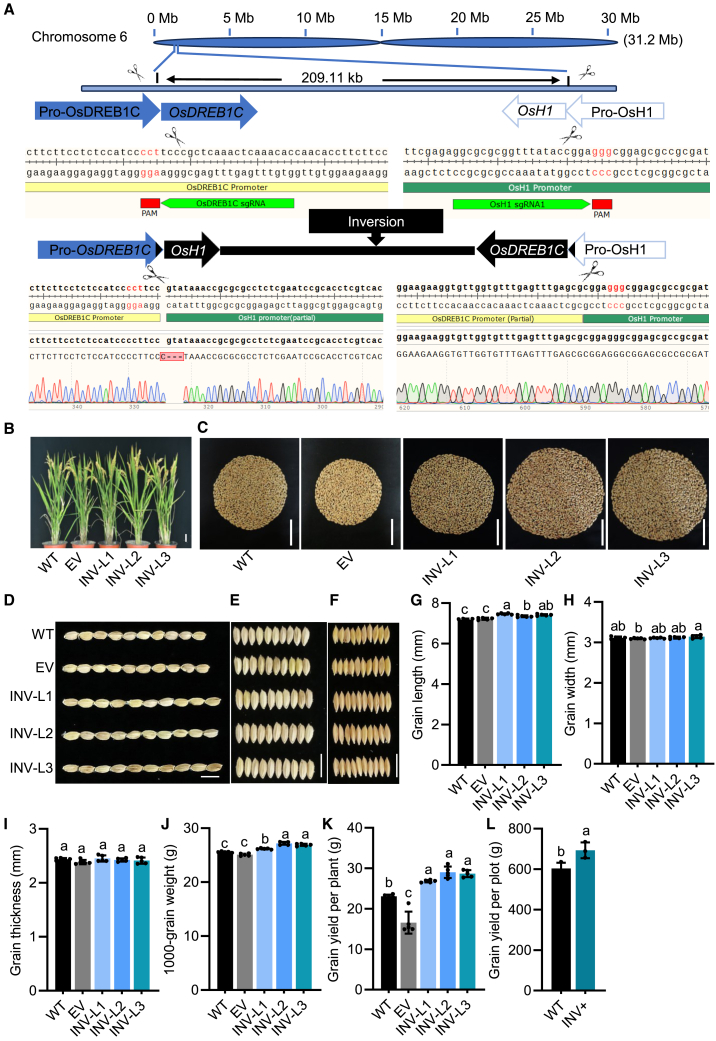
CRISPR-Cas9-mediated *OsDREB1C* promoter swap to enhance rice grain characteristics and yield. **(A)** Promoter swap between *OsDREB1C* and *OsH1*. Homozygous inversions were identified in T_1_ plants and confirmed by sequencing. Protospacer adjacent motif sequences are highlighted in red, while the adjacent sgRNAs are marked by green arrows. **(B)** Gross plant morphology of WT, empty vector control (EV), and 3 T_2_ homozygous inversion lines (INV-L1 to 3) at the mature stage. **(C)** Yield comparison for individual plants of WT, EV, and INV-L1 to 3. **(D–F)** Grain morphologies of WT, EV, and INV-L1 to 3, displaying grain length **(D)**, width **(E)**, and thickness **(F)**. **(G–I)** Grain characteristics, including length **(G)**, width **(H)**, and thickness **(I)**. **(J** and **K)** Thousand-grain weight **(J)** and yield per plant **(K)** for WT, EV, and INV-L1 to 3. **(L)** Grain yield of INV+, T_2_ homozygous inversion plants, compared to WT, with 30 plants per plot. Scale bars: 5 cm **(B–C)** and 1 cm **(D–F)**. Data are means ± SD of five biological replicates in **(G–K)** and three biological replicates in **(L)**. Statistical significance was determined by Duncan’s multiple range tests. Different letters above the bars indicate significant differences (*p* < 0.05).

From these 10 positive calli, 180 T_0_ plants were developed. Sanger sequencing of PCR amplicons revealed 78 inversion-positive T_0_ plants, representing 5 unique genotypes and yielding a positivity rate of 43.33% (78 of 180). Genotypes 1–3 showed seamless integration on the right side (Supplemental Figure 3B and 3C; Supplemental Table 4), and their T_1_ plants were selected for further evaluation. Transgene-free plants with homozygous inversion were identified using six primer pairs. Among these, the primers OsDREB1C-F2/OsH1-F1 and OsDREB1C-R2/OsH1-R1 were designed to detect the inversion sites, while OsH1-F1/OsH1-R1 and OsDREB1C-F2/OsDREB1C-R2 confirmed the presence of the original endogenous genes. To detect the potential introduction of the exogenous *Cas9* and *hygromycin-resistance* genes, the Cas9-F/R and Hyg-F/R primer pairs were utilized. Among the plants tested, only 4 from 3 independent lines (inversion lines 1–3 [INV-L1 to 3]) of genotype 1 showed successful PCR amplification for the inversion and no amplification for the other primers (Figure 1A and Supplemental Figure 4). These results confirmed that the four plants were indeed transgene-free and carried a homozygous inversion. Thus, our characterization efforts were focused on these homozygous T_2_ plants.

In the homozygous T_2_ plants from INV-L1 to 3, *OsDREB1C* expression in the flag leaves at the filling stage was enhanced by at least 10-fold compared to the wild type (WT) and the EV controls, while *OsH1* expression was decreased to approximately 0.21% (Supplemental Figure 5). Importantly, the expression levels of neighboring genes flanking *OsH1* and *OsDREB1C* remained largely unchanged (Supplemental Figure 5). A comprehensive phenotypic evaluation of the homozygous T_2_ population in Hangzhou’s paddy field showed improvements in grain length and weight, panicle number, number of grains per plant, setting percentage, and harvest index (Figure 1B–1J; Supplemental Table 5). Notably, compared to WT and EV controls, grain yield per plant increased by 21%–28.33% and yield per plot by 14.97%, with no observable fitness penalties (Figure 1K and 1L; Supplemental Table 5). More importantly, grain quality remained unaffected, indicating that our CRISPR-Cas9-mediated knockup of *OsDREB1C* improved rice yield without compromising grain quality (Supplemental Figure 6; Supplemental Table 6). These improvements were consistently inherited in the T_3_ generation during field trials in Lingshui, confirming the stability of our genetic modification (Supplemental Table 7).

Transgenic breeding is a well-established and efficient method for improving crop traits by introducing exogenous genes into plant genomes. However, this approach often results in random integration of foreign genes, which can cause unintended mutations and undesirable position effects on transgene expression (Liu et al., 2024). In contrast to conventional transgenic breeding, we took advantage of the precision of CRISPR-Cas9 technology to achieve a targeted genomic inversion, resulting in the knockup of *OsDREB1C*, a gene known to increase grain number and weight, boosting rice yield. Overall, our CRISPR-Cas9-based knockup strategy is effective for crop breeding, improving crucial agronomic traits while maintaining a transgene-free genomic background.

## Funding

This work was supported by the Projects of International Cooperation of the National Natural Science Foundation of China (10.13039/501100001809NSFC, 31961143016); the Innovation Project of the 10.13039/501100005196Chinese Academy of Agricultural Sciences, High-Quality and Resistant Hybrid Rice Germplasm Creation and New Variety Development with International Competitiveness (2022KJCX45 and YBXM2437); Zhejiang Provincial Science and Technology Projects (2022R51009); the Inner Mongolia Breeding Joint Research Project (YZ2023004); the Xing’an League Science and Technology Project (2023DXZD0001); the National Rice Industrial Technology System (CARS-01-11); and the Open Project Program of the State Key Laboratory of Rice Biology and Breeding (20240303).

## Acknowledgments

No conflict of interest is declared.

## Author contributions

Y.L., X.Z., G.P., and L.C. designed the experiments. Y.L., X.Z., Yingxin Zhang, B.W., G.W., Yue Zhang, G.L., Q.L., and W.W. performed the experiments. Y.L., X.Z., X.S., D.C., Y.H., G.P., and L.C. analyzed the data. G.Y. and S.C. supervised the project. Y.L., G.P., and L.C. wrote the manuscript. All authors reviewed and approved the final version for publication.

## Supplemental information

Document S1. Supplemental Figures 6, Supplemental Tables 1–8, and supplemental materials and methods

Document S2. Article plus supplemental information
