# Supplementary material for: CRISPR-Cas9-mediated knockup of OsDREB1C enhances rice yield without compromising grain quality
Source: Plant Commun. 2025 Jun 27;6(10):101433. doi: 10.1016/j.xplc.2025.101433 (PMC12545883; doi:10.1016/j.xplc.2025.101433)
Supplement: Document S2. Article plus supplemental information [file mmc2.pdf]

Plant Communications
Correspondence

CellPress
Partner Journal

CRISPR-Cas9-mediated knockup of *OsDREB1C* enhances rice yield without compromising grain quality

Dear Editor,

CRISPR-Cas9 genome editing has revolutionized plant breeding by enabling precise genetic modifications in staple crops such as rice and wheat (Zhu et al., 2020). Through targeted gene knockout or knockdown, this technology has demonstrated remarkable success in improving yield potential, stress resistance, and nutritional quality (Zhang et al., 2017, 2021; Li et al., 2024). In rice breeding, CRISPR-Cas9 has been successfully used to induce genomic structural variation (SV) and enhance pesticide resistance (Lu et al., 2021). However, inversions, an often-overlooked type of SV, remain rarely reported despite their potential for crop improvement (Hu et al., 2024). Inversions occur when CRISPR-Cas9 induces double-strand breaks at the promoter and coding regions of two oppositely oriented genes on the same chromosome. This inversion-style rearrangement can swap the genes' promoters, altering their expression (Lu et al., 2021), and potentially enhancing desirable phenotypes such as yield.

In this study, we report the CRISPR-Cas9 engineering of SVs in *OsDREB1C* (*Os06g0127100*), a rice yield-enhancing gene, by increasing grain number and weight (Wei et al., 2022). We hypothesized that *OsDREB1C* could be repositioned under a promoter with high transcript activity following CRISPR-Cas9-mediated inversion. Database analyses (The Rice Annotation Project Database, Rice Genome Annotation Project, and CRISPR- Applicable Functional Redundancy Inspector in Rice) identified the histone H1 gene *OsH1* (*Os06g0130800*) as an ideal candidate (Supplemental Figure 1A and 1B; Supplemental Table 1), located 209.11 kb from *OsDREB1C* (Figure 1A) with opposite transcriptional orientation. Functional redundancy of *OsH1* was confirmed by two knockout mutants (*osh1-1* and *osh1-2*) that showed no agronomic defects (Supplemental Figure 2; Supplemental Table 2), consistent with histone H1's conserved role in epigenetic regulation across species (Wierzbicki and Jerzmanowski, 2005; Rea et al., 2012). We then determined the expression patterns of both *OsH1* and *OsDREB1C* in the *japonica* rice cultivar Jingeng818 during the booting stage. Both genes were ubiquitously expressed, with *OsH1* exhibiting remarkably higher levels of gene expression than *OsDREB1C* (Supplemental Figure 1C). Given the opposite directions of *OsDREB1C* and *OsH1* on chromosome 6, we employed CRISPR-Cas9 to edit their 5′ untranslated regions (UTRs) using single guide RNAs (sgRNAs). Our goal was to invert the genomic segment between the two genes' cutting sites and swap their promoters. A vector containing two sgRNAs was first expressed in rice protoplasts, as verified by PCR using the specific primer pairs OsDREB1C-F2/OsH1-F1 and OsDREB1C-R2/OsH1-R1, respectively (Supplemental Table 8). As expected, inversion of the genomic segment containing *OsDREB1C-OsH1* was successfully achieved (Supplemental Figure 3A). The sgRNA-expressing vector and its empty vector (EV) were next introduced into rice calli of the Jingeng818 background via *Agrobacterium*-mediated transformation. From 8.85 g of transformed calli, we obtained 165 hygromycin-resistant calli. Inversion events were validated in ten calli, showing five distinct genotypes on the right side (Promoter$_{OsH1}$::OsDREB1C). This represented a positive callus percentage of 6.06% (10 of 165) (Supplemental Tables 3 and 4).

From these 10 positive calli, 180 T_0 plants were developed. Sanger sequencing of PCR amplicons revealed 78 inversion-positive T_0 plants, representing 5 unique genotypes and yielding a positivity rate of 43.33% (78 of 180). Genotypes 1–3 showed seamless integration on the right side (Supplemental Figure 3B and 3C; Supplemental Table 4), and their T_1 plants were selected for further evaluation. Transgene-free plants with homozygous inversion were identified using six primer pairs. Among these, the primers OsDREB1C-F2/OsH1-F1 and OsDREB1C-R2/OsH1-R1 were designed to detect the inversion sites, while OsH1-F1/OsH1-R1 and OsDREB1C-F2/OsDREB1C-R2 confirmed the presence of the original endogenous genes. To detect the potential introduction of the exogenous *Cas9* and *hygromycin-resistance* genes, the Cas9-F/R and Hyg-F/R primer pairs were utilized. Among the plants tested, only 4 from 3 independent lines (inversion lines 1–3 [INV-L1 to 3]) of genotype 1 showed successful PCR amplification for the inversion and no amplification for the other primers (Figure 1A and Supplemental Figure 4). These results confirmed that the four plants were indeed transgene-free and carried a homozygous inversion. Thus, our characterization efforts were focused on these homozygous T_2 plants.

In the homozygous T_2 plants from INV-L1 to 3, *OsDREB1C* expression in the flag leaves at the filling stage was enhanced by at least 10-fold compared to the wild type (WT) and the EV controls, while *OsH1* expression was decreased to approximately 0.21% (Supplemental Figure 5). Importantly, the expression levels of neighboring genes flanking *OsH1* and *OsDREB1C* remained largely unchanged (Supplemental Figure 5). A comprehensive phenotypic evaluation of the homozygous T_2 population in Hangzhou's paddy field showed improvements in grain length and weight, panicle number, number of grains per plant, setting percentage, and harvest index (Figure 1B–1J; Supplemental Table 5). Notably, compared to WT and EV controls, grain yield per plant increased by 21%–28.33% and yield per plot by 14.97%, with no observable fitness penalties (Figure 1K and 1L; Supplemental Table 5). More importantly, grain quality remained unaffected, indicating that our CRISPR-Cas9-mediated knockup of *OsDREB1C* improved rice yield

Plant Communications 6, 101433, October 13 2025 © 2025 The Authors. Published by Elsevier Inc. on behalf of CAS Center for Excellence in Molecular Plant Sciences, Chinese Academy of Sciences, and Chinese Society for Plant Biology.
This is an open access article under the CC BY license (http://creativecommons.org/licenses/by/4.0/).

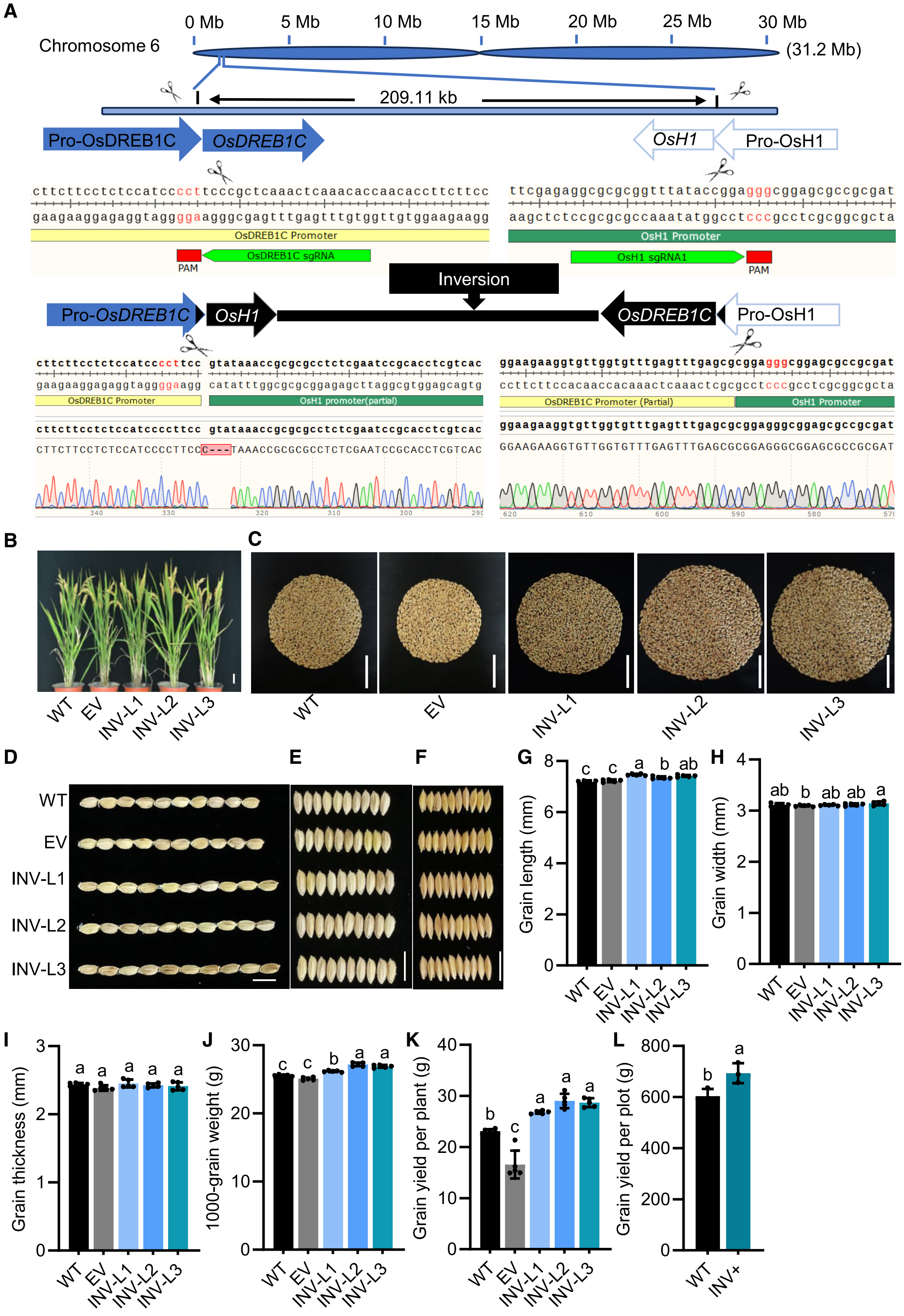

Figure 1. CRISPR-Cas9-mediated *OsDREB1C* promoter swap to enhance rice grain characteristics and yield.

(A) Promoter swap between *OsDREB1C* and *OsH1*. Homozygous inversions were identified in T_1 plants and confirmed by sequencing. Protospacer adjacent motif sequences are highlighted in red, while the adjacent sgRNAs are marked by green arrows.

(B) Gross plant morphology of WT, empty vector control (EV), and 3 T_2 homozygous inversion lines (INV-L1 to 3) at the mature stage.

(legend continued on next page)

without compromising grain quality (Supplemental Figure 6; Supplemental Table 6). These improvements were consistently inherited in the T_3 generation during field trials in Lingshui, confirming the stability of our genetic modification (Supplemental Table 7).

Transgenic breeding is a well-established and efficient method for improving crop traits by introducing exogenous genes into plant genomes. However, this approach often results in random integration of foreign genes, which can cause unintended mutations and undesirable position effects on transgene expression (Liu et al., 2024). In contrast to conventional transgenic breeding, we took advantage of the precision of CRISPR-Cas9 technology to achieve a targeted genomic inversion, resulting in the knockup of *OsDREB1C*, a gene known to increase grain number and weight, boosting rice yield. Overall, our CRISPR-Cas9-based knockup strategy is effective for crop breeding, improving crucial agronomic traits while maintaining a transgene-free genomic background.

FUNDING
This work was supported by the Projects of International Cooperation of the National Natural Science Foundation of China (NSFC, 31961143016); the Innovation Project of the Chinese Academy of Agricultural Sciences, High-Quality and Resistant Hybrid Rice Germplasm Creation and New Variety Development with International Competitiveness (2022KJCX45 and YBXM2437); Zhejiang Provincial Science and Technology Projects (2022R51009); the Inner Mongolia Breeding Joint Research Project (YZ2023004); the Xing'an League Science and Technology Project (2023DXZD0001); the National Rice Industrial Technology System (CARS-01-11); and the Open Project Program of the State Key Laboratory of Rice Biology and Breeding (20240303).

ACKNOWLEDGMENTS
No conflict of interest is declared.

AUTHOR CONTRIBUTIONS
Y.L., X.Z., G.P., and L.C. designed the experiments. Y.L., X.Z., Yingxin Zhang, B.W., G.W., Yue Zhang, G.L., Q.L., and W.W. performed the experiments. Y.L., X.Z., X.S., D.C., Y.H., G.P., and L.C. analyzed the data. G.Y. and S.C. supervised the project. Y.L., G.P., and L.C. wrote the manuscript. All authors reviewed and approved the final version for publication.

SUPPLEMENTAL INFORMATION
Supplemental information is available at *Plant Communications Online*.

Received: January 6, 2025
Revised: March 24, 2025
Accepted: June 25, 2025
Published: June 27, 2025

Yanmin Luo[1,2,4,7], *Xiaodeng Zhan*[1,7], *Yingxin Zhang*[1], *Beifang Wang*[1], *Guanqi Wang*[1], *Yue Zhang*[1], *Guzi Li*[1], *Qunen Liu*[1], *Xihong Shen*[1], *Daibo Chen*[1], *Yongbo Hong*[1,3], *Weixun Wu*[1], *Guoyou Ye*[5], *Shihua Cheng*[1], *Gang Pan*[6,*] and *Liyong Cao*[1,2,3,4,*]

[1]State Key Laboratory of Rice Biology and Breeding, China National Rice Research Institute, Hangzhou 311400, China
[2]Northern Rice Research Center of Baoqing, Shuangyashan 155600, China
[3]National Nanfan Research Institute (Sanya), Chinese Academy of Agricultural Sciences, Sanya 572025, China
[4]Key Laboratory of Northern Japonica Rice Research in Heilongjiang Province, Baoqing Northern Rice Research Center, Baoqing, Heilongjiang 155600, China
[5]International Rice Research Institute, Los Banos, Laguna 4031, Philippines
[6]Department of Agronomy, Zhejiang University, Hangzhou 310058, China
[7]These authors contributed equally to this article.
***Correspondence: Gang Pan (pangang12@zju.edu.cn), Liyong Cao (caoliyong@caas.cn)**

https://doi.org/10.1016/j.xplc.2025.101433

REFERENCES
Hu, H., Scheben, A., Wang, J., Li, F., Li, C., Edwards, D., and Zhao, J. (2024). Unravelling inversions: Technological advances, challenges, and potential impact on crop breeding. Plant Biotechnol. J. **22**:544–554.

Li, B., Sun, C., Li, J., and Gao, C. (2024). Targeted genome-modification tools and their advanced applications in crop breeding. Nat. Rev. Genet. **25**:603–622.

Liu, P., Panda, K., Edwards, S.A., Swanson, R., Yi, H., Pandesha, P., Hung, Y.H., Klaas, G., Ye, X., Collins, M.V., et al. (2024). Transposase-assisted target-site integration for efficient plant genome engineering. Nature **631**:593–600.

Lu, Y., Wang, J., Chen, B., Mo, S., Lian, L., Luo, Y., Ding, D., Ding, Y., Cao, Q., Li, Y., et al. (2021). A donor-DNA-free CRISPR-Cas-based approach to gene knock-up in rice. Nat. Plants **7**:1445–1452.

Rea, M., Zheng, W., Chen, M., Braud, C., Bhangu, D., Rognan, T.N., and Xiao, W. (2012). Histone H1 affects gene imprinting and DNA methylation in Arabidopsis. Plant J. **71**:776–786.

Wei, S., Li, X., Lu, Z., Zhang, H., Ye, X., Zhou, Y., Li, J., Yan, Y., Pei, H., Duan, F., et al. (2022). A transcriptional regulator that boosts grain yields and shortens the growth duration of rice. Science **377**:eabi8455.

Wierzbicki, A.T., and Jerzmanowski, A. (2005). Suppression of histone *H1* genes in Arabidopsis results in heritable developmental defects and stochastic changes in DNA methylation. Genetics **169**:997–1008.

Zhang, H., Zhang, J., Lang, Z., Botella, J.R., and Zhu, J.K. (2017). Genome editing-principles and applications for functional genomics research and crop improvement. Crit. Rev. Plant Sci. **36**:291–309.

Zhang, S., Zhang, R., Gao, J., Song, G., Li, J., Li, W., Qi, Y., Li, Y., and Li, G. (2021). CRISPR/Cas9-mediated genome editing for wheat grain quality improvement. Plant Biotechnol. J. **19**:1684–1686.

Zhu, H., Li, C., and Gao, C. (2020). Applications of CRISPR-Cas in agriculture and plant biotechnology. Nat. Rev. Mol. Cell Biol. **21**:661–677.

(C) Yield comparison for individual plants of WT, EV, and INV-L1 to 3.
(D–F) Grain morphologies of WT, EV, and INV-L1 to 3, displaying grain length **(D)**, width **(E)**, and thickness **(F)**.
(G–I) Grain characteristics, including length **(G)**, width **(H)**, and thickness **(I)**.
(J and K) Thousand-grain weight **(J)** and yield per plant **(K)** for WT, EV, and INV-L1 to 3.
(L) Grain yield of INV+, T_2 homozygous inversion plants, compared to WT, with 30 plants per plot.
Scale bars: 5 cm **(B–C)** and 1 cm **(D–F)**. Data are means ± SD of five biological replicates in **(G–K)** and three biological replicates in **(L)**. Statistical significance was determined by Duncan's multiple range tests. Different letters above the bars indicate significant differences ($p < 0.05$).

Plant Communications, Volume *6*

Supplemental information

CRISPR-Cas9-mediated knockup of *OsDREB1C* enhances rice yield without compromising grain quality

Yanmin Luo, Xiaodeng Zhan, Yingxin Zhang, Beifang Wang, Guanqi Wang, Yue Zhang, Guzi Li, Qunen Liu, Xihong Shen, Daibo Chen, Yongbo Hong, Weixun Wu, Guoyou Ye, Shihua Cheng, Gang Pan, and Liyong Cao

1. **Materials and Methods**
2. **Supplementary Figures**

Figure S1: Structural details of full cage (**1**)
Figure S2: Structural details of half cage (**2**)
Figure S3: ^{1}H NMR spectra of full cage (**1**)
Figure S4: Synthesis and characterisation of m-TPT
Figure S5: ^{1}H NMR spectra of half cage (**2**)
Figure S6: 2D COSY NMR spectra of half cage (**2**)
Figure S7: ^{13}C NMR spectra of half cage (**2**)
Figure S8: Schematics of 1-ENap ⊂ Full Cage (**1)** H-G complexation
Figure S9: UV-vis absorption spectra of 1-ENap, full cage and 1-ENap ⊂ **1**
Figure S10: Schematics of 1-ENap ⊂ Half Cage ⊂ **2)** H-G complexation
Figure S11: UV-vis absorption spectra of 1-ENap, half cage and 1-ENap ⊂ **2**
Figure S12: ^{1}H NMR spectra of 1-ENap, full cage and 1-ENap ⊂ **1** H-G complex
Figure S13: ^{1}H NMR spectra of 1-ENap, half cage and 1-ENap ⊂ **2** H-G complex
Figure S14: ^{1}H NMR spectra of 2-ENap, half cage and 2-ENap ⊂ **2** H-G complex
Figure S15: ^{1}H NMR spectra of 2-ENap, full cage and 2-ENap ⊂ **1** H-G complex
Figure S16: ^{1}H NMR spectra of 6-Ome-2-ENap ⊂ Half Cage (**2)** H-G complex
Figure S17: UV-vis absorption spectra of 2-ENap ⊂ **1** / **2** and 6-Ome-2-ENap ⊂ **2**
Figure S18: ^{1}H NMR spectra of 2-ENap ⊂ **1** complex with increasing amount of 2-ENap
Figure S19: ^{1}H NMR spectra of 2-ENap ⊂ **2** complex with increasing amount of 2-ENap
Figure S20: ^{1}H NMR-based binding curves for 2-ENap ⊂ **1** and 2-ENap ⊂ **2** complexes
Figure S21: Photoreaction scheme of 1-ENap ⊂ **1** H-G complex with visible light
Figure S22: Photoproduct identification in photosystem 1-ENap ⊂ **1**: GC-MS data
Figure S23: ^{1}H NMR spectra of oxidative product 1-acetonaphthone
Figure S24: HR-MS chromatogram of [4+2] cycloaddition product 1-Phe-1-Nap
Figure S25: Photoreaction scheme of 1-ENap ⊂ **2** H-G complex with visible light
Figure S26: Photoproducts identification in photosystem 1-EN ⊂ **2**: GC-MS data
Figure S27: Photoproduct identification in photosystem 2-ENap ⊂ **1** / **2**: GC-MS data
Figure S28: HR-MS chromatogram of [4+2] cycloaddition product 4-Phe-2-Nap
Figure S29: ^{1}H NMR spectra of oxidative product 2-acetonaphthone
Figure S30: ^{1}H NMR spectra of oxidative product 6-methoxy-2-acetonaphthone
Figure S31: Structure of substrates attempted for [4+2] cycloaddition inside half cage (**2**)
Figure S32: ^{1}H NMR spectra of [4+2] cycloaddition product 1-Phe-1-Nap
Figure S33: 2D HSQC spectra of [4+2] cycloaddition product 1-Phe-1-Nap
Figure S34: 2D COSY spectra of [4+2] cycloaddition product 1-Phe-1-Nap
Figure S35: ^{1}H NMR spectra of 1-Phe-1-Nap with multiplicities and coupling pattern
Figure S36: ^{1}H NMR spectra of [4+2] cycloaddition product 4-Phe-2-Nap
Figure S37: ^{1}H NMR spectra of [4+2] cycloaddition product 2,6'-di-Ome-5-Phe-2-Nap
Figure S38: 2D HSQC spectra of [4+2] cycloaddition product 2,6'-di-Ome-5-Phe-2-Nap
Figure S39: 2D COSY spectra of [4+2] cycloaddition product 2,6'-di-Ome-5-Phe-2-Nap
Figure S40: ^{1}H NMR of 2,6‘-di-Ome-5-Phe-2-Nap with multiplicities & coupling pattern
Figure S41: ^{13}C NMR spectra of [4+2] cycloaddition product 1-Phe-1-Nap
Figure S42: ^{13}C NMR spectra of [4+2] cycloaddition product 4-Phe-2-Nap
Figure S43: ^{13}C NMR spectra of [4+2] cycloaddition product 2,6'-di-Ome-5-Phe-2-Nap
Figure S44: Optimized conformations of 1-ENap molecules within half cage.
Figure S45: Optimized structure of reactants, proposed intermediates and products
Figure S46: Theoretical and experimental ^{13}C NMR spectra of reactants and products
Figure S47: Theoretical and experimental ^{1}H NMR spectra of reactants and products
Figure S48: Theoretical and experimental FT-IR spectra of 1-ENap and 1-Phe-1-Nap
Figure S49: The possible coupling products in reaction
Figure S50: ^{1}H NMR spectra of alkyne hydration (oxidation) products 1-acetonaphthone-H_3/D_3

Figure S51: ^{1}H NMR spectra of alkyne hydration(oxidation) products 2-acetonaphthone-H_3/D_3
Figure S52: ^{1}H NMR spectra of oxidation products 2-acetyl-6-methoxynaphthalene-H_3/D_3
Figure S53: Proposed reaction mechanism of alkyne hydration reaction inside full cage
Figure S54: Transient absorption spectra of 1-ENap ⊂ **1** H-G complex
Figure S55: Photocatalytic cycles of 1-ENap ⊂ **2** H-G complex system
Figure S56: Control experiment: Product displacement by reactant molecules
Figure S57: Control experiments in photoreaction with 1-ENap system
Figure S58: Control experiments in photoreaction with 1-ENap + Pd(en)$(NO_3)_2$ system
Figure S59: Control experiment: Displacement of 1-ENap by 1-naphthaldehyde molecule
Figure S60: Control experiment: Reaction in presence of 1-naphthaldehyde- GCMS data
Figure S61: Reaction scheme for photoredox reactions with Ru(II)- / Ir(III) photocatalysts
Figure S62: GC-MS chromatograms for reactions with Ru(II)- / Ir(III) photocatalysts
Figure S63: Transient absorption spectra of 1-ENap ⊂ **2** H-G complex
Figure S64: Transient absorption spectra of 2-ENap ⊂ **2** and 6-Ome-2-ENap ⊂ **2**
Figure S65: Comparative study of decay kinetics of the radical cation state in H_2O & D_2O
Figure S66: HR-MS chromatogram of cycloaddition product after photoreaction in D_2O
Figure S67: ^{1}H NMR spectra of cycloaddition product after photoreaction in D_2O
Figure S68: HR-MS data of radical intermediates trapped with 4-hydroxy-TEMPO

1. Materials and methods

1.1Materials

All syntheses were carried out using commercial reagents unless otherwise specified. Following are the sources for the respective chemicals: 3-cyano-pyridine, activated charcoal and silver nitrate, ethylene diamine, palladium chloride, sodium hydroxide, 1-naphthaldehyde, 1-acetonaphthone, 1-ethynylnaphthalene, 6-methoxy-2-ethynylnaphthalene, 2,4,6-tri(4-pyridyl)-1,3,5-triazine, 2-ethynnylnaphthalene were purchased from Sigma-Aldrich and TCI Chemicals Pvt. Ltd., respectively. Solvents and other reagents were purchased from SD Fine Chemicals and Sigma-Aldrich. Heavy water (D_2O) d_3-chloroform ($CDCl_3$) were procured from Sigma-Aldrich and used as supplied for the chemical reactions as well as for the NMR measurement.

1.2Experimental Methods

Synthesis of [Pd(en)Cl_2] complex: To the brown suspension of $PdCl_2$ (1.0 g, 5.64 mmol) in water (20 mL), concentrated hydrochloric acid was added dropwise to dissolve the $PdCl_2$. 1.5 mL of ethylenediamine (= en) was diluted with 10 mL of water and then added dropwise, yielding a pink colour precipitate. On continuous addition of ethylene diamine, the solid dissolved. The pH of solution was adjusted to 2 with addition of half diluted–concentrated hydrochloric acid. The reaction mixture was stored in fridge (0-4°C) for overnight. The yellow colour solid was filtered and washed with water and then with methanol for several times. Solid was dried in vacuum (1.2 g, 90% yield).

Synthesis of [Pd(en)$(ONO_2)_2$] complex: [Pd(en)Cl_2] complex (1.112 g, 4.68 mmol, 1 equiv.) was suspended in water (50 mL) and $AgNO_3$ (1.591g, 9.36 mmol, 2 equiv.) was added. The suspension was stirred at room temperature with exclusion from light for 12 h.[1; 2] The white solid of AgCl formed was filtered off and the filtrate was evaporated in rotary evaporator. The yellow solid was collected (0.970 g, 87%).

Synthesis of 2,4,6-tris(3-pyridyl)-1,3,5-triazine: 3-cyanopyridine (15 g, 144 mmol, 10 equiv.) was taken in a round bottom flask and heated to 150 °C under reflux condition. Powdered NaOH (576 mg, 14.4 mmol, 1 equiv.) was added to the resulting liquid of 3-cyanopyridine. Precipitation started within 5 minutes of stirring and heating was continued for 1.5 h at 150 °C to complete the precipitation. The solid

was washed with acetone 4–5 times and dissolved in hydrochloric acid (125 mL, 2 M). Activated charcoal was added and sonicated for 30 min. The suspension was filtered using Whatman filter paper and neutralized with NaOH (75 mL, 5M). The white precipitate was filtered and washed with water and acetone for several times then dried in vacuum.[3] The solid product was dissolved in chloroform and filtered then recollected using rotary evaporator under vacuum. After drying the yield was 54 % (8.1 g).

Synthesis of $[Pd_6L'_4].12NO_3$ half cage: The 2,4,6-tris(3-pyridyl)-1,3,5-triazine ligand (31.2 mg, 0.1 mmol, 2 equivalent) was suspended in aqueous solution (1.5 mL) of $[Pd(en)(ONO_2)_2]$ (43.57 mg ,0.15 mmol, 3 equivalent) as per reported procedure. The reaction mixture was heated at 70 °C for 30 min gave a clear colourless solution, from which a yellowish coloured crystalline material was obtained by evaporation.[4] Final product was characterized by ^1H NMR, ^{13}C NMR, 2D COSY spectroscopy.

Synthesis of $[Pd_6L_4].12NO_3$ full Cavity: The 2,4,6-tri(4-pyridyl)-1,3,5-triazine (0.86 g, 2.75 mmol, 2 equivalent) was suspended in aqueous solution (80 mL H_2O) of $Pd(en)(ONO_2)_2$ [en = ethylenediamine] (1.2 g, 4.13 mmol, 3 equivalent), 3:2 stoichiometric ratio as per procedure reported.[5; 6] The obtained suspension was stirred at room temperature (RT) for 24 h and subsequently heated at 80°C with stirred for another 2 h. A trace amount of insoluble material was filtered out, and the clear solution was rotary evaporated to give 1.66 (80%) of cage as pale-yellow material.

NMR Characterization: All ^1H and ^{13}C NMR spectra were recorded on Varian-600 (600 MHz) and Bruker-800 (800 MHz) spectrometers. 2-D COSY and HSQC NMR measurements were performed on Bruker-800 (800 MHz) spectrometers.

Gas Chromatography-Mass Spectrometry (GCMS): GC analyses were performed on Agilent 7890A GC system with an FID detector using J & W DB–1 column (10 m, 0.1 mm I.D.). The system was connected with 5975C inert XL EI/CI MSD (with triple axis detector).

Steady state absorption measurements: All the steady state absorption measurements were carried out in a JASCO V-670 spectrophotometer.

FT-IR measurements: Fourier Transform InfraRed spectra of the samples were recorded with a Bruker FTIR Invenio-S spectrophotometer equipped with thermal detector DLaTGS (Deuterated L-alanine doped triglycine sulphate). All measurements were done in transmission mode at room temperature after drop casting the sample at KBr cell. The instrument has spectral resolution of 2 cm^{-1}.

Transient absorption (pump-probe) measurements: All pump-probe measurements had been carried out using an ultrafast transient absorption spectrometer. An oscillator (Coherent Micra-5 Mode-locked Ti:sapphire Laser) is used to generate output of 810 nm pulse laser at repetition rate of 80 MHz with bandwidth of ~100 nm. The output from the oscillator was amplified around 10^6 times using a commercial regenerative amplifier (Coherent Legend Elite Ultrafast Amplifier Laser system). The output from the amplifier is 30 fs/4 mJ per pulse with a repetition rate of 1 kHz and bandwidth of ~65 nm. The 400 nm pump pulse is generated by frequency doubling process using a beta barium borate (BBO) crystal. A portion of the 810 nm output from the amplifier is used to get 400 nm pump pulse. For measurements, a pump beam was attenuated to ~100−300 nJ per pulse depending on the signal obtained for the host-guest complexes. The white-light broadband probe continuum (440−1400 nm) was generated by focusing a portion of the amplified 810 nm output on a 2 mm thick sapphire, and then directed to a multichannel detector procured from Ultrafast Systems. The pump and probe pulses were focused within the sample cuvette and overlapped spatially as well as temporally to get the signal from H-G complex. A motorized translation stage having a quadra-pass mirror assembly is used to vary the time delay between pump and probe pulses. To minimize the photodegradation of the samples all the measurements were performed in flow cuvette. A peristaltic pump is attached to a commercially procured flow cuvette of 1- or 2-mm path length for continuous flow of solution. The instrument response function (IRF) was determined ~150 fs for 400 nm pump pulses, from an optical Kerr effect (OKE) experiment on a 1 mm glass. Kinetic fitting of the raw data traces had been performed using a mathematical program for deconvoluting the time constants from the recorded IRF in IGOR pro 5 software. Surface Xplorer (from Ultrafast Systems) and Glotaran software were used to do the global and target analyses of transient absorption data respectively.

Kinetics data fitting procedure: The kinetic analyse of transient absorption data was carried out using IGOR pro 5 wavemetrics software with written program to deconvolve the lifetime from the measured

IRF. The procedure produced decay time constants and their respective amplitudes. The equation employed for fitting is given below

$$y(t) = c \sum_{i=1}^{4} a_i \exp^{-\frac{t}{\tau_i}} * Gassian\ IRF$$

Where, c = scaling factor.
a_i = amplitudes of the exponentials.
τ_i = time constants.
Gaussian IRF = instrument response function

We have used maximum three exponential fit for 1-ENap ⊂ Half Cage and 1-ENap ⊂ Full Cage to analyse excited state CT absorption, both in visible and near IR (NIR) wavelength window. The transient absorption data analysis involved time zero correction to account for the chirp of the probe pulse before proceeding to kinetic analysis.

Global and target analysis using Glotaran: The Glotaran software[7] is used to perform global target analysis of the transient absorption data which consisted of a three-dimensional matrix of wavelength, difference absorption intensity and time. Initial number of components is estimated from singular value decomposition (SVD) of the raw data matrix through global target analysis (GTA). This number was further optimized with the help of a sequential global model having increasing lifetimes. This produced principal decay/rise time constants of the populations and evolution associated difference spectra (EADS). In this model, the white-light dispersion was described by a second-order polynomial and the Gaussian instrument response function was 0.15 ps for 400 nm pump. Singular vectors of the matrix of residuals have to be structureless to have the best fit. The evolution associated difference spectra was interpreted as a weighted sum of species-associated difference spectra (SADS) and thus might not represent pure species. Therefore, to resolve the of species-associated difference spectra from the evolution associated difference spectra, a target model was set up by introducing branching from specific states which mainly influences the amplitude of the species associated difference spectra without altering their shape or the decay/rise time constants. We have used a three-compartment model A → B → C where B and C are going to the ground state again, for excited CT state dynamics of 1-ENap ⊂ Half cage, 2-ENap ⊂ Half cage and 6-Ome-2-ENap ⊂ Half cage H-G complexes. This resulted into the species-associated difference spectra of the different species and microscopic rate constants. Singular value decomposition is used to judge the quality of the residual matrices and for a satisfactorily kinetic model, the first left- and right singular vectors have to be structureless.

Incarceration protocol for 1-ethynynaphthalene in full cage: Liquid 1-ethynylnaphthalene (1-ENap) (50.32 μmol), was suspended in 2 mL D_2O solution of cage (10.06 μmol). The solution was stirred at room temperature for 1 h in the dark condition which resulted a brownish-yellow coloured solution of 1-ENap ⊂ Full Cage complex. The solution was filtered (0.45 μm Millex nonsterile filter) and cleared solution was taken for proton NMR and other experiments. ^1H NMR analysis of the brownish-yellow colour solution shown the quantitative formation of 1-ENap ⊂ Full Cage complex and relative peak area integration of cage protons with 1-ENap protons provided a quantitative estimate of the loading which was on an average 4 molecules of 1-ENap per full cage (1:4 H-G complexation).

Incarceration protocol for 2-ethynynaphthalene in full cage: Solid 2-ethynylnaphthalene (2-ENap) (50.32 μmol), was suspended in 2 mL D_2O solution of cage (10.06 μmol). The solution was stirred at room temperature for 1.5 h in the dark condition which resulted a brownish-yellow coloured solution of 2-ENap ⊂ Full Cage complex. The solution was filtered (0.45 μm Millex nonsterile filter) and cleared solution was taken for proton NMR and other experiments. ^1H NMR analysis of the brownish-yellow colour solution shown the quantitative formation of 2-ENap ⊂ Full Cage complex and relative peak area integration of cage protons with 2-ENap protons provided a quantitative estimate of the loading which was on an average 4 molecules of 2-ENap per full cage (1:4 H-G complexation).

Incarceration protocol for 1-ethynynaphthalene in half cage: Liquid 1-ethynylnaphthalene (1-ENap) (50.32 µmol), was suspended in 2 mL D_2O solution of cage (10.06 µmol). The solution was stirred at room temperature for 1 h in the dark condition which resulted a brownish-yellow coloured solution of 1-ENap ⊂ Half Cage complex. The solution was filtered (0.45 µm Millex nonsterile filter) and cleared solution was taken for proton NMR and other experiments. ^1H NMR analysis of the brownish-yellow colour solution shown the quantitative formation of 1-ENap ⊂ Half Cage complex and relative peak area integration of cage protons with 1-ENap protons provided a quantitative estimate of the loading which was on an average two molecules of 1-ENap per half cage (1:2 H-G complexation).

Incarceration protocol for 2-ethynynaphthalene in half cage: Solid 2-ethynylnaphthalene (2-ENap) (50.32 µmol), was suspended in 2 mL D_2O solution of cage (10.06 µmol). The solution was stirred at room temperature for 1.5 h in the dark condition which resulted a brownish-yellow coloured solution of 2-ENap ⊂ Half cage complex. The solution was filtered (0.45 µm Millex nonsterile filter) and cleared solution was taken for proton NMR and other experiments. ^1H NMR analysis of the brownish-yellow colour solution shown the quantitative formation of 2-ENap ⊂ Half Cage complex and relative peak area integration of cage protons with 2-ENap protons provided a quantitative estimate of the loading which was on an average two molecules of 2-ENap per half cage (1:2 H-G complexation).
Similar incarceration protocol was used for 6-methoxy-2-ethynynaphthalene in full cage and half cage.

1.3Computational Details

All ab initio electronic structure calculations were conducted using version 5.0.4 of the ORCA package.[8] For each potential product structure, conformer sampling was performed using version 2.12 of the CREST package, employing the GFN2-XTB method and the ALPB implicit solvation model for $CHCl_3$.[9; 10] Conformers within an energy range of up to 6 kcal/mol were then extracted and used as input for the CENSO package[11] to compute NMR spectra. In CENSO, initial pre-screening was done using the B97 functional[12] with D3 dispersion correction[13; 14], followed by geometry optimization and NMR calculations with the PBE0 functional[15] and D4 dispersion correction[16]. For all electronic structure calculations in CENSO, we used the def2-TZVPP basis set[17] and the SMD implicit solvation model[18] for $CHCl_3$. Structures within 2.5 kcal/mol were considered for NMR calculations, and chemical shifts were averaged over conformers using TMS as the reference compound, with its NMR spectra computed at the same theoretical level.

Since IR experiments were performed in film, the conformers obtained from CENSO were reoptimized in vacuum (without solvation) using the PBE0-D4 functional and the def2-TZVPP basis set. The default convergence criteria of 10^{-6} au for energy and 10^{-4} au for gradients were applied for geometry optimization. IR spectra were computed for the most stable conformer using the same functional and basis set.
For the IR spectra, the 1-ENap were computed using PBE0-D4 with the def2-TZVPP basis set and then scaled by 0.94516 so that the high-frequency vibrational mode (corresponding to the CH stretch of sp carbon) obtained in theory matched the experimental spectra. After obtaining the scaling factor, the IR spectra of the 1-Phe-1-Nap is computed and scaled by the same factor. Molecular vibrations are visualized using Avogadro version 1.2.[19]

2. Supplementary figures

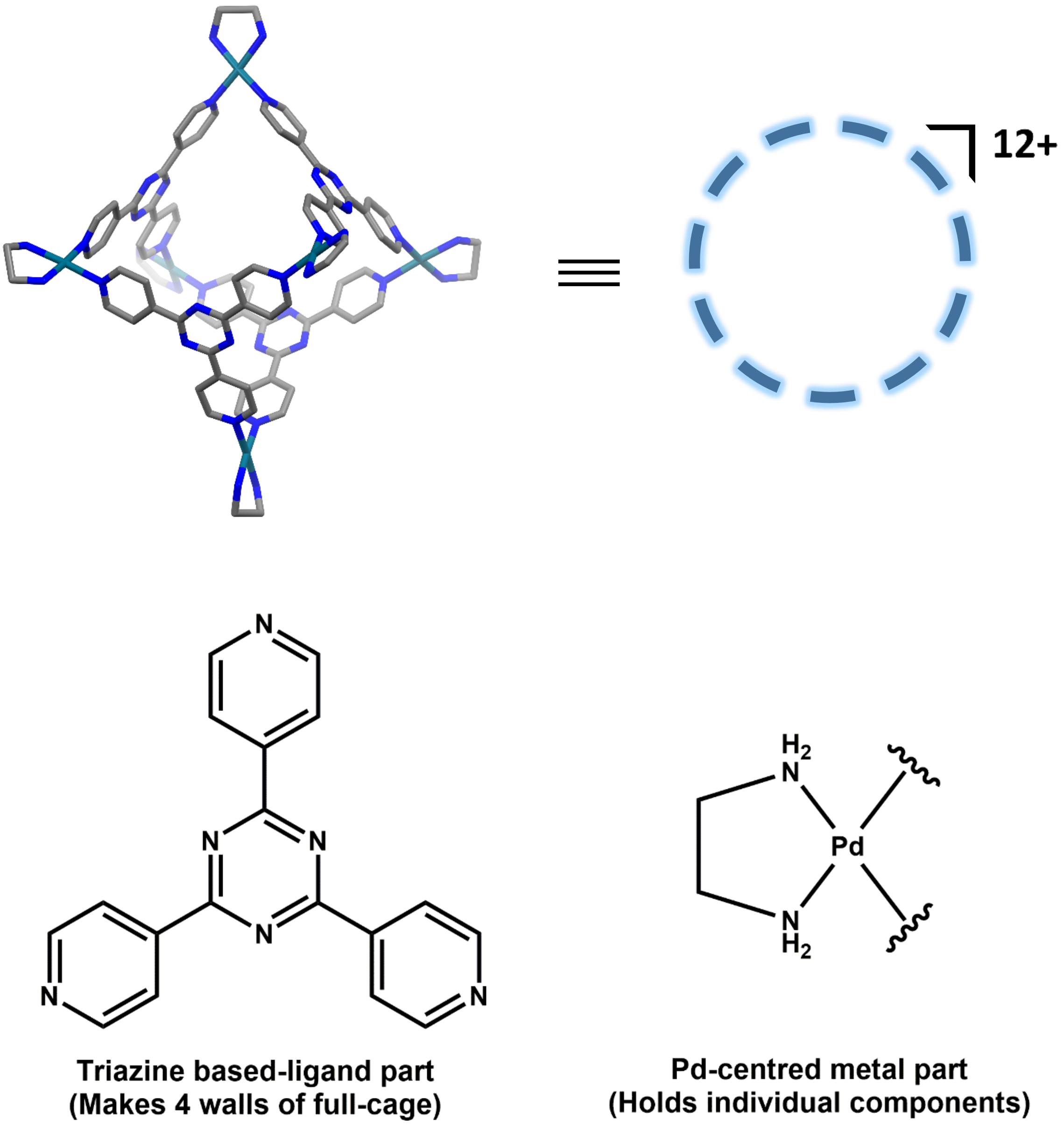

Figure S1. Structural details of full cage: Octahedral-shaped nanocavity (full cage: **1**). Crystal structure of $[Pd_6L_4]^{12+}$ nanocage (left) obtained from CCDC (277006) and its schematic representation is shown in right (top panel). The structure is visualized using mercury.[20] The structure of triazine-based (p-TPT) ligand and metal-unit (shown in bottom panel) used in the assembly of full cage.

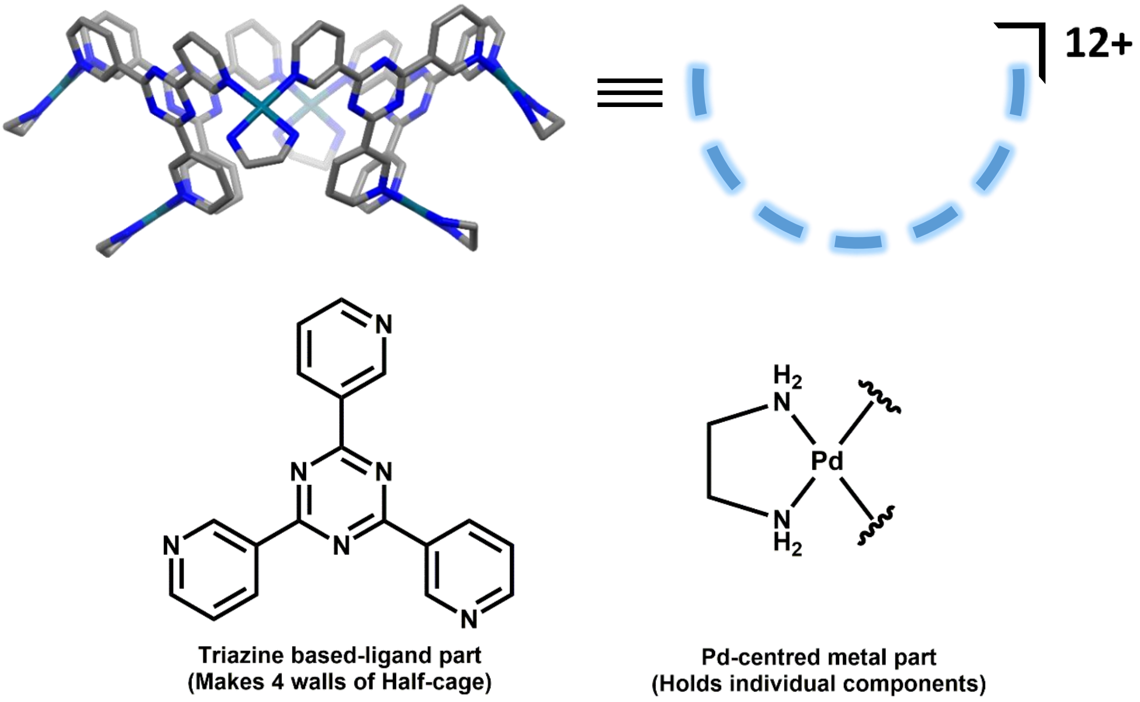

Figure S2. Structural details of half cage: Square-pyramidal-shaped cavity (half cage: **2**). Crystal structure of $[Pd_6L'_4]^{12+}$ nanocage (left) obtained from CCDC (112237) and its schematic representation is shown in right (top panel). The structure is visualized using mercury.[20] The structure of triazine-based (m-TPT) ligand and metal-unit (shown in bottom panel) used in the assembly of half cage.

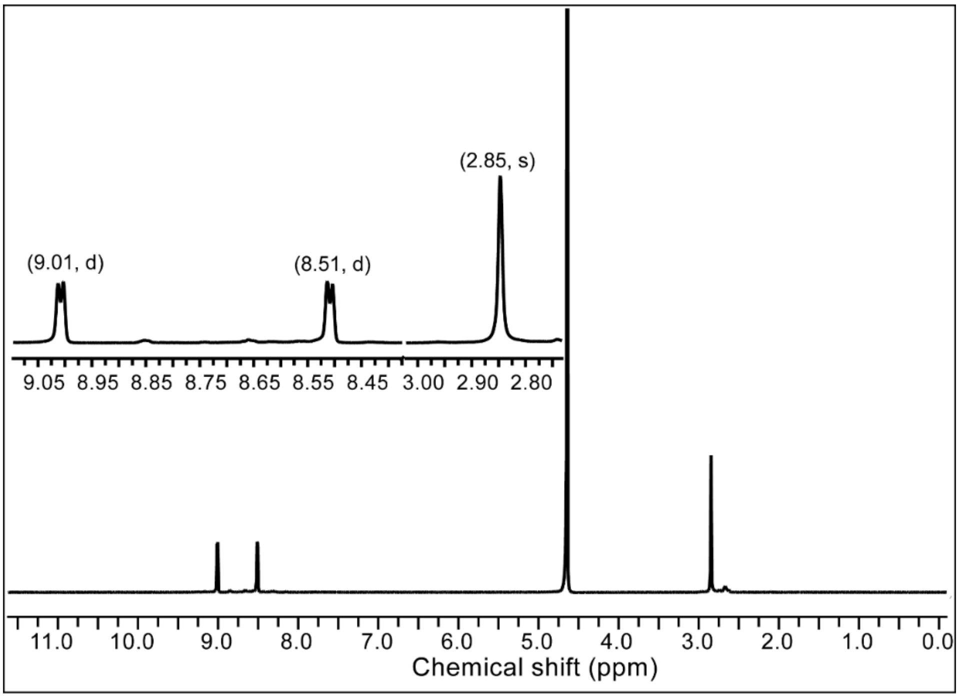

Figure S3. ^{1}H NMR spectrum of synthesized $[Pd_6L_4]^{12+}$ Full cage: (600 MHz, D_2O solvent, RT). Two doublet peaks at δ 9.01 ppm (24 H) and 8.51 ppm (24 H) are from PyH_α and PyH_β protons respectively, singlet peak at δ 2.85 ppm (24 H) corresponds to en-CH2 protons. Water protons present in D_2O give rise to a peak at δ 4.65 ppm.

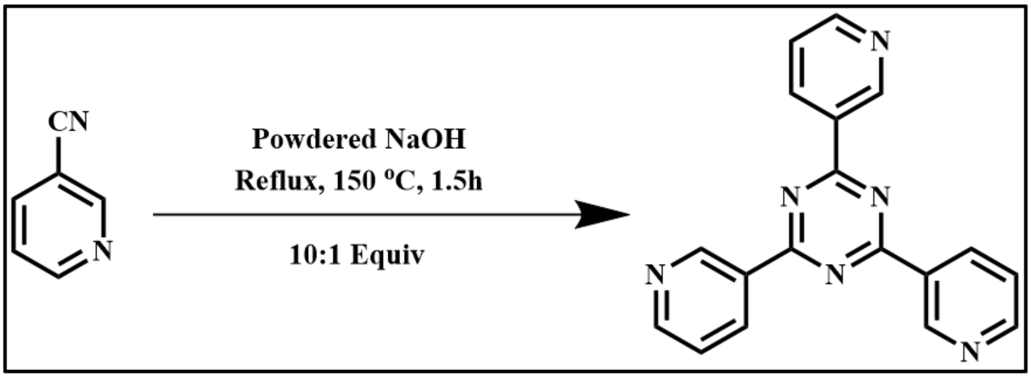

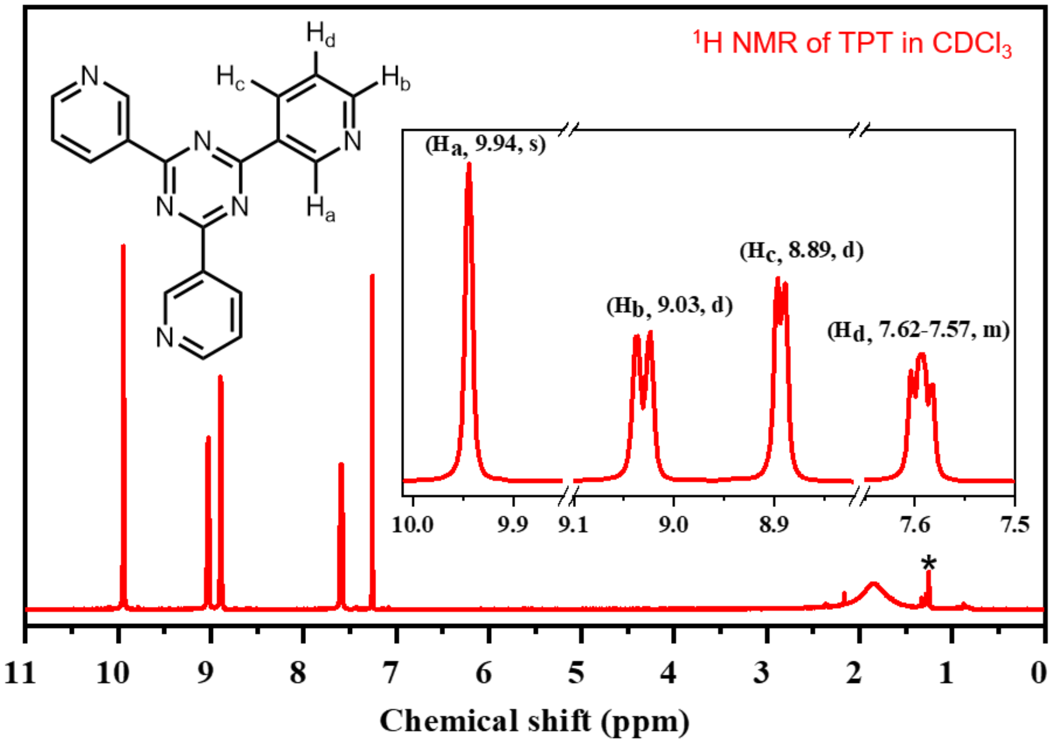

Figure S4. Synthesis and characterisation of m-TPT: The Synthetic scheme for 2,4,6-tris(3-pyridyl)-1,3,5-triazine (m-TPT) and ^{1}H NMR spectra (600 MHz, $CDCl_3$, RT) are shown in top and bottom panel respectively. Due the symmetry of TPT molecule there are only four sets of proton peaks are expected from the structure which are assigned using notations like H_a (9.94 ppm, 1H, s), H_b (9.03 ppm, 1H, d), H_c (8.89 ppm, 1H, d) and H_d (7.62-7.57 ppm, 1H, m). [The asterisk marked is the impurity in solvent].

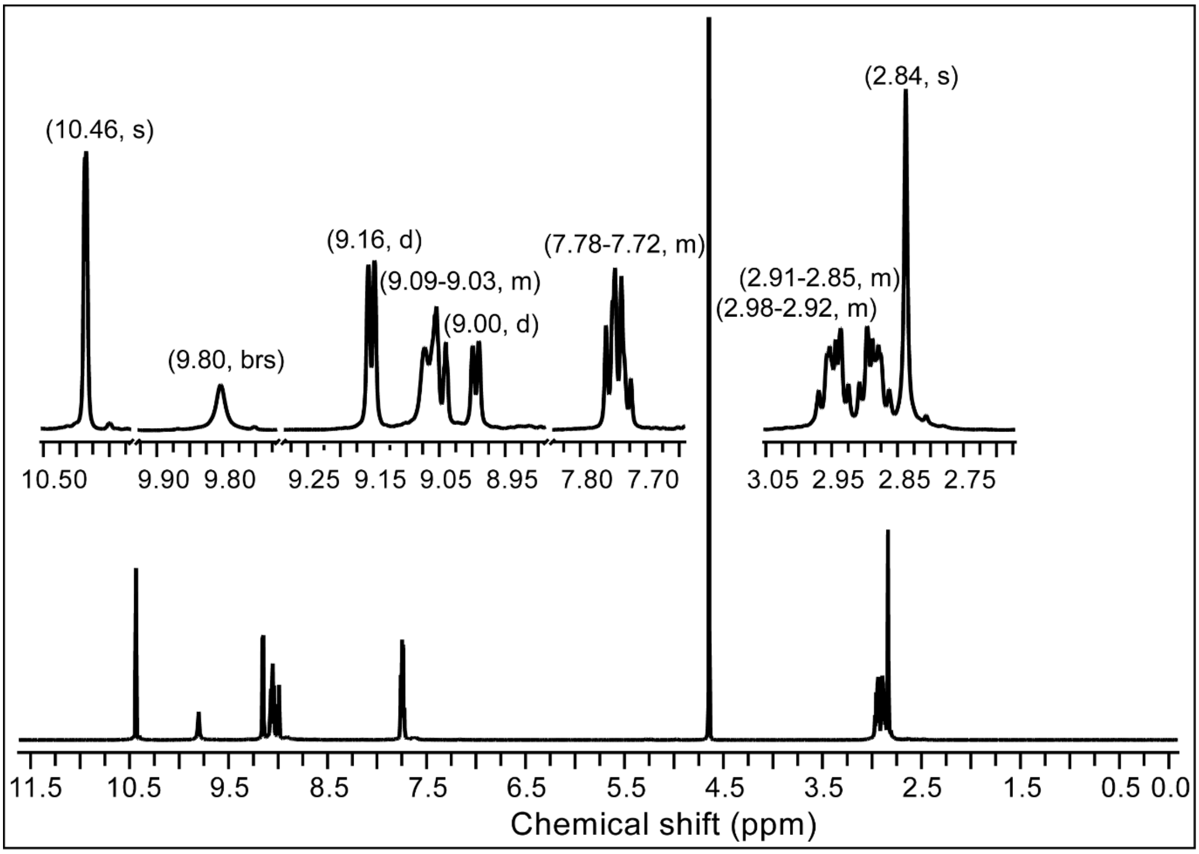

Figure S5. ^{1}H NMR spectrum of synthesized $[Pd_6L'_4]^{12+}$ half cage: (800 MHz, D_2O, RT). The peak position and their multiplicity are as follow: (10.46, s, 8H), (9.80, brs, 4H), (9.16, d, 8H), (9.09-9.03, m, 12H), (9.00, d, 4H), (7.78-7.72, m, 12H), (2.98-2.92, m, 8H), (2.91-2.85, m, 8H), (2.84, s, 8H).

This ^{1}H NMR data is explained in following way: The cavity structure has two different kinds of electronic environment for protons in the aromatic region, one from the top and other from the bottom surface of cavity. The relative peak area integration should be 2:1 because the number of pyridyl rings are in similar ratios and hence same for the number of protons. The structure suggests that there should be 2 singlet, 4 doublets and 2 triplet peaks in intensity ratio of 2:1. The protons of capping ligands ethylenediamine are present in three different electronic environments so there should be three set of proton peaks in intensity ratios of 1:1:1.

The recorded ^{1}H NMR data: In aromatic region there are 2 singlets, 2 doubles peaks with intensity ratio 2:1 and 2 multiplets peaks with intensity ratio 1:1. This ^{1}H NMR data was further analysed with the help of 2D correlation spectroscopy (COSY) data (Figure S6). The 2D COSY data shows the through bond coupling pattern between the protons. The recorded 2D COSY data showed the coupling pattern and peak multiplicity of respective proton signals. Correlation diagram clearly showed the presence of 2 singlet, 4 doublets and 2 triplet peaks. The multiplets present in ^{1}H NMR data in aromatic region is actually result of the overlap of 2 doublets (9.09 - 9.03 ppm, m) and 2 triplets (7.78 - 7.72 ppm, m).

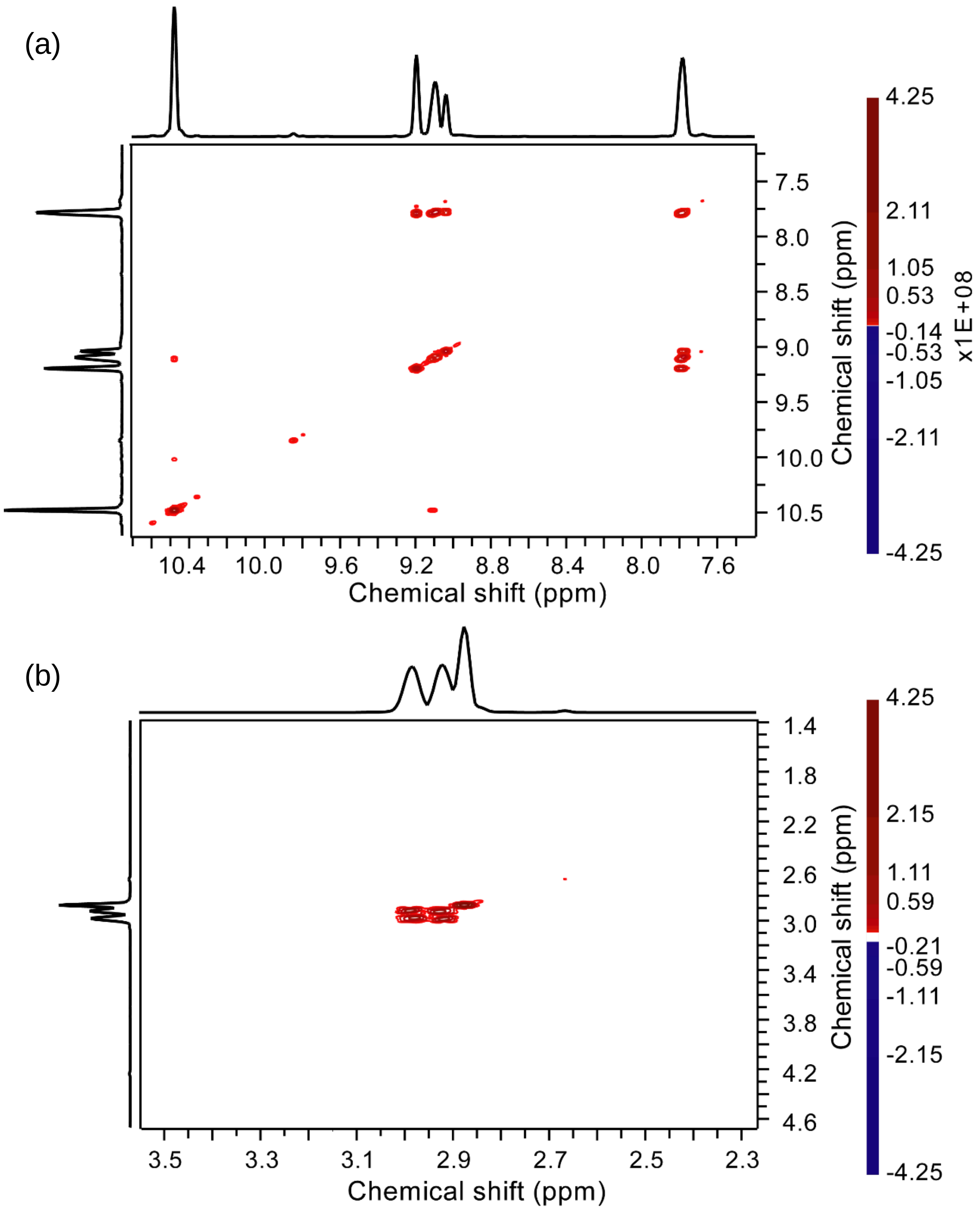

Figure S6. 2D COSY NMR spectra of half cage: The two-dimensional cross correlation NMR spectra of half cage is recorded using 800 MHz instrument in D_2O solvent. The aromatic region of the spectra (a) shows the coupling pattern of protons present in triazene based ligand unit and (b) shows the coupling pattern of protons present in capping ligand which is ethylenediamine. The 2D COSY NMR spectra supports in the analysis of the ^1H NMR spectra of half cage.

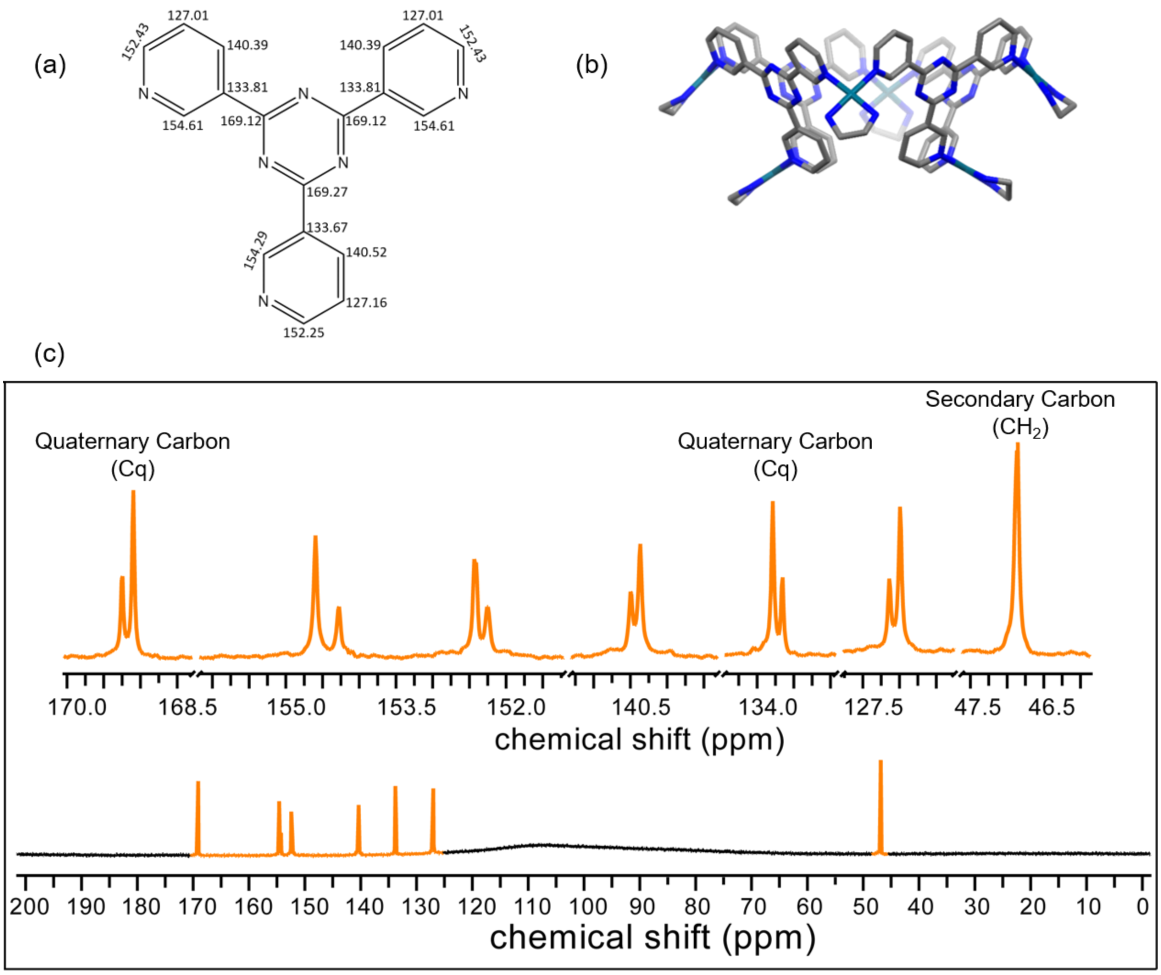

Figure S7. ^{13}C NMR spectrum of synthesized $[Pd_6L'_4]^{12+}$ half cage: (800 MHz, D_2O, RT). The peak positions of carbon atoms present in half cage is shown in figure (a) labelled on TPT structure (representing the actual position of carbon atoms in half cage) and the values are as follow: 169.27 (C_q), 169.12 (C_q), 154.61 (CH), 154.29 (CH), 152.43 (CH), 152.25 (CH), 140.52 (CH), 140.39 (CH), 133.81 (C_q), 133.67 (C_q), 127.16 (CH), 127.01 (CH), 46.87 (CH_2). The electronic environment in upper part of the cage is different from the lower part (Figure b) that's why two sets of peaks are present from pyridine rings. (c) ^{13}C NMR spectra of half cage with zoomed in version in inset which clearly shows two sets of peaks from upper and bottom part of the cavity.

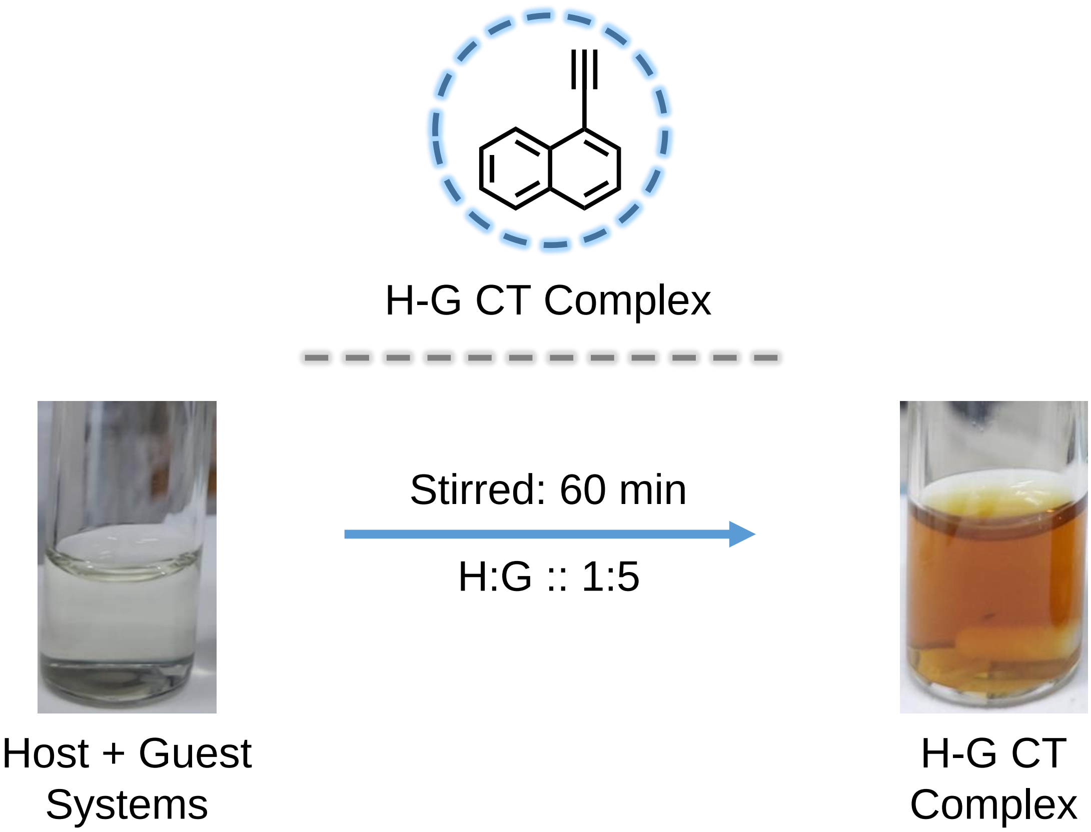

Figure S8. Schematics of 1-ENap ⊂ full cage (1) H-G complexation: The H-G CT complex formation was done by incarcerating guest molecule inside full cage. To make H-G CT complex first the full cage was dissolved in aqueous medium by heating at 80 ºC and then kept it to get back to room temperature. The guest molecule was added in excess amount over the cavity solution. The solution was stirred at room temperature for a fix time which finally resulted a brownish-yellow coloured solution.

For 1-ethynylnaphthalene ⊂ full cage host-guest charge transfer complex, host and guest were taken in 1:5 equivalent ratio [5 mM, 2 mL cage solution (10.06 μmol) + 50.32 μmol of 1-ethynylnaphthalene]. Stirring at room temperature for 1h resulted coloured solution shown in figure.

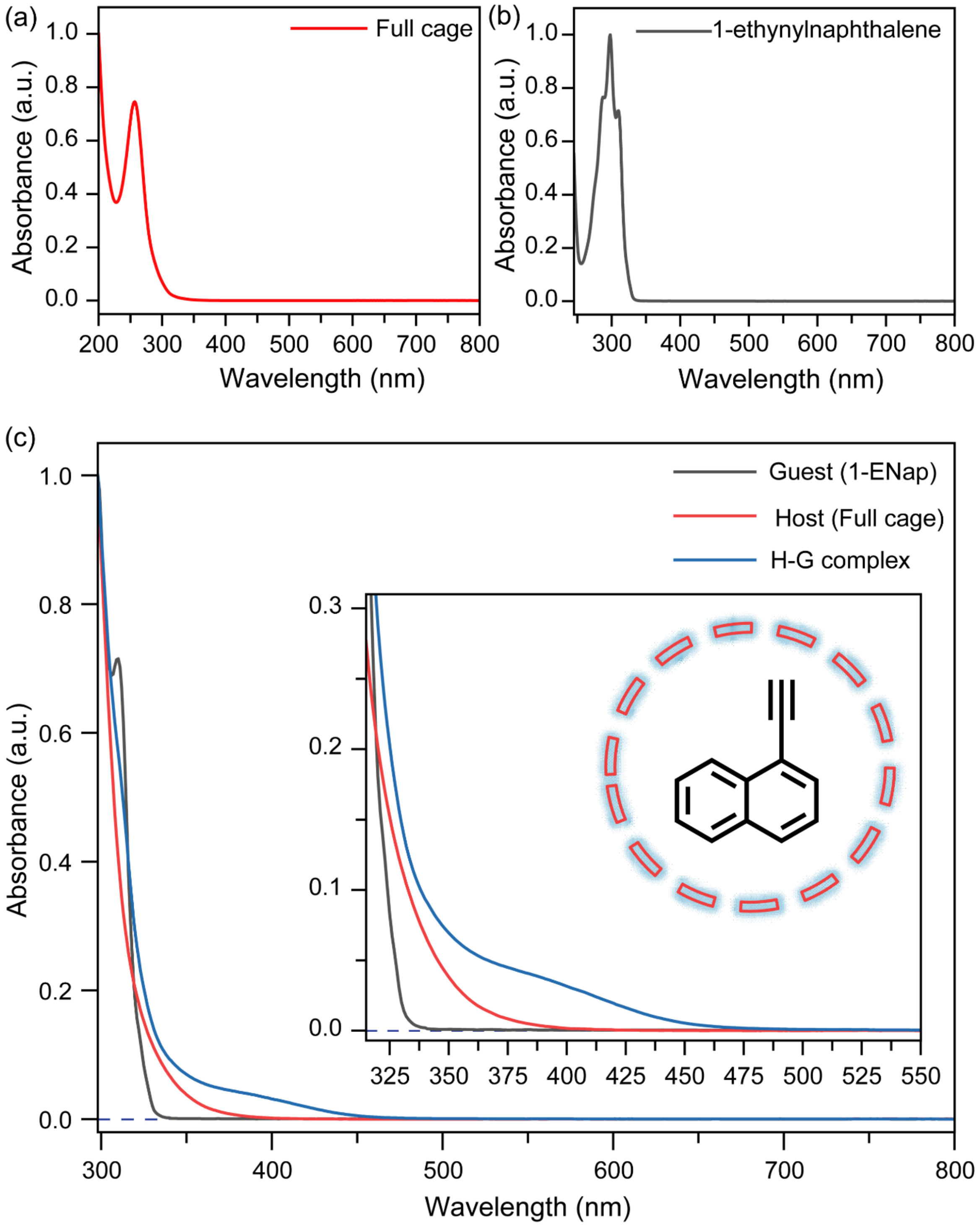

Figure S9. UV-vis absorption spectra of 1-ENap, full cage and 1-ENap ⊂ 1: (a) UV-visible absorption spectra of full cage in H_2O, (b) 1-ethynylnaphthalene (1-ENap) in chloroform. (c) combined absorption spectra of host (red colour), guest (black colour) and H-G complex (1-ENap ⊂ **1**, blue colour). Full cage and 1-ENap has absorption only in ultraviolet region (a, b). The H-G complex has an extra absorption band in wavelength region 365 - 475 nm which is assigned as charge transfer (CT) band (inset). The charge transfer band is broad due to orientational heterogeneity in host-guest complex system.

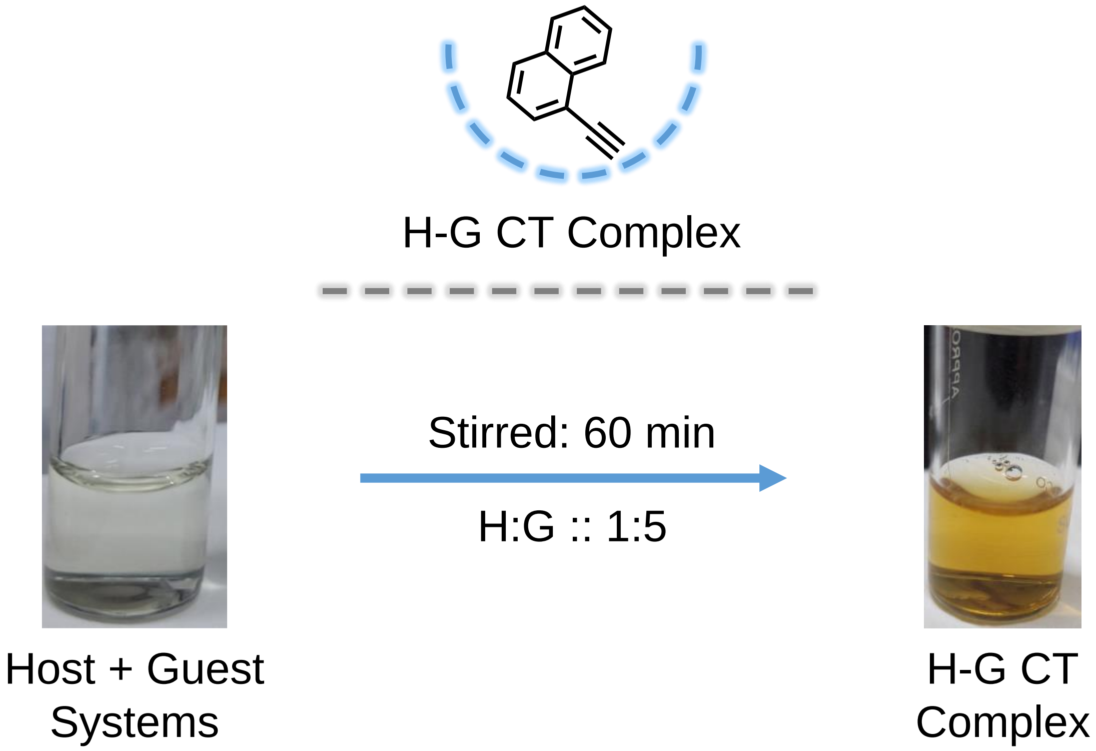

Figure S10. Schematics of 1-ENap ⊂ half cage (2) H-G complexation: The H-G CT complex formation was done by incarcerating guest molecule inside half cage. To make H-G CT complex half cage was dissolved in aqueous medium and the guest molecule was added in excess amount over the cavity solution. The solution was stirred at room temperature for a fix time which finally resulted a coloured solution.

For 1-ethynylnaphthalene ⊂ Half cage host-guest charge transfer complex, host and guest were taken in 1:5 equivalent ratio [5 mM, 2 mL cage solution (10.06 μmol) + 50.32 μmol of 1-ethynylnaphthalene]. Stirring at room temperature for 1 h resulted brownish-yellow coloured solution shown in figure.

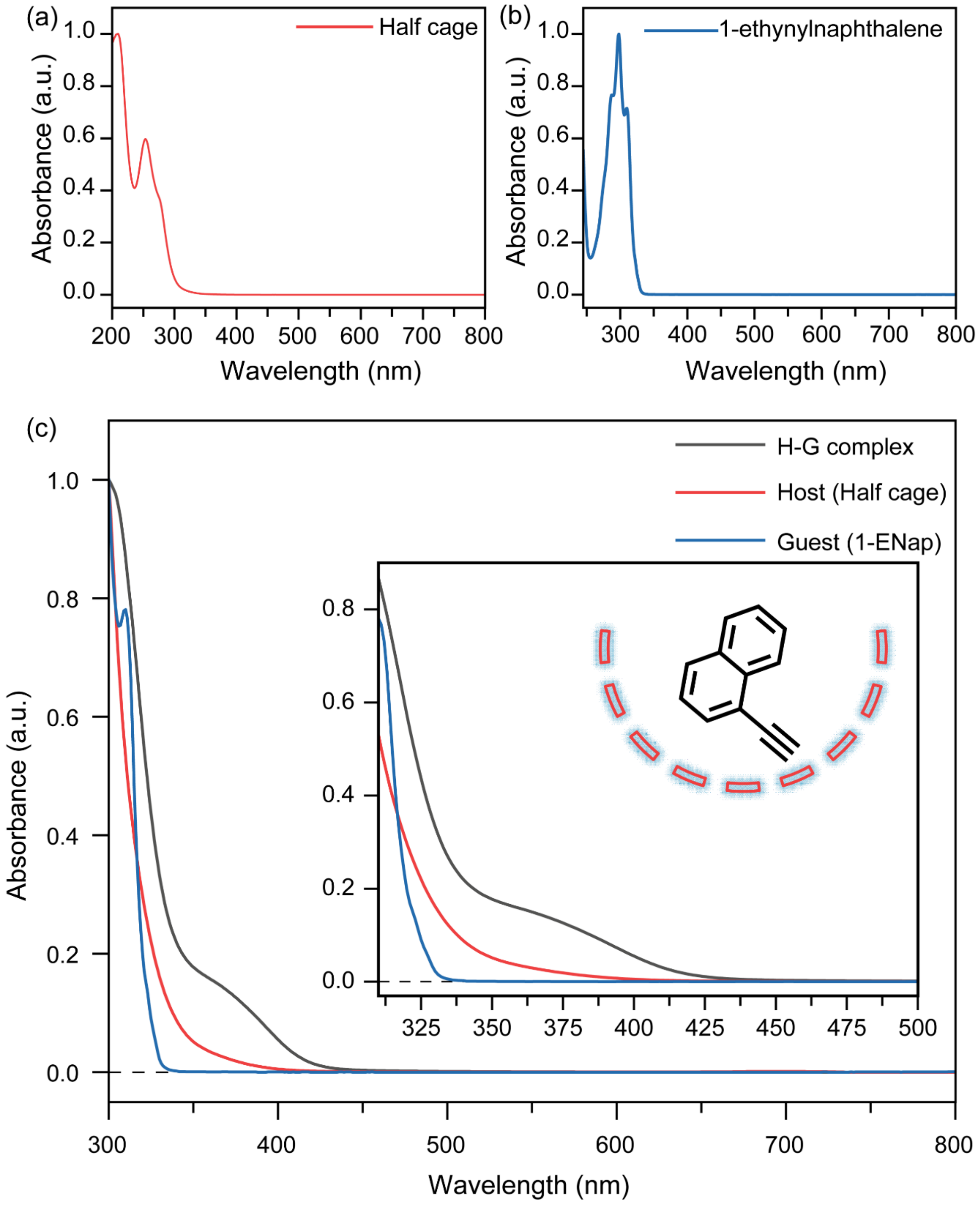

Figure S11. UV-vis absorption spectra of 1-ENap, half cage and 1-ENap ⊂ 2: (a) UV-visible absorption spectra of half cage in H_2O, (b) 1-ethynylnaphthalene (1-ENap) in chloroform. (c) combined absorption spectra of host (red colour), guest (blue colour) and H-G complex (1-ENap ⊂ **2**, black colour). Half cage and 1-ENap has absorption only in ultraviolet region (a, b). The H-G complex has an extra absorption band in wavelength region 350-445 nm which is assigned as charge transfer (CT) band (inset). The charge transfer band is broad due to orientational heterogeneity in host-guest complex system.

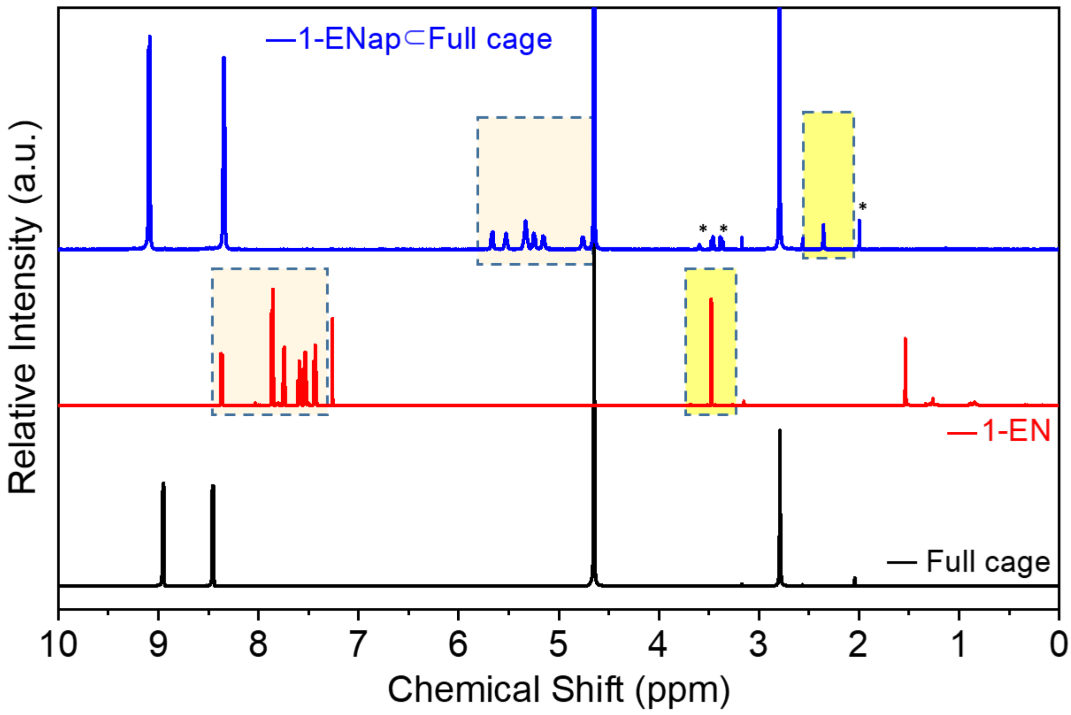

Figure S12. ^{1}H NMR spectra of 1-ENap, full cage and 1-ENap ⊂ Full Cage (1) H-G complex: ^{1}H NMR spectra of full cage in D_2O (black), 1-Ethynylnaphthalene in $CDCl_3$ (red) and 1-ENap ⊂ **1** in D_2O (blue) recorded using 600 MHz NMR instrument. Dotted regions show that guest proton peaks have been upfield shifted after incarceration inside full cage. The protons of 1-Ethynylnaphthalene which were in aromatic region (in between 8.36 ppm – 7.43 ppm) have moved in upfield direction (in between 5.56 ppm – 4.76 ppm) after incarceration in cage (in pale-yellow dotted region). In similar way the alkynyl proton (in yellow dotted region) in aliphatic region at 3.47 ppm also moved to 2.35 ppm after incarceration. The packing of guest molecules inside the cage induces a change in the electronic environment around the protons of cage as well. This change in the electronic environment led the change in the peak positions (chemical shift) of cage protons that's why there is shift in the peak positions of two aromatic protons (from triazine core) of full cage after guest (1-ENap) encapsulation with respect to their peak positions in free full cage. Relative peak area integration between protons of 1-ENap and full cage suggests that there is on an average four guest molecules per cage i.e., 1:4 H-G complexation between 1-ENap and full cavity. [Asterisk (*) marked peaks are some impurities from solvents]

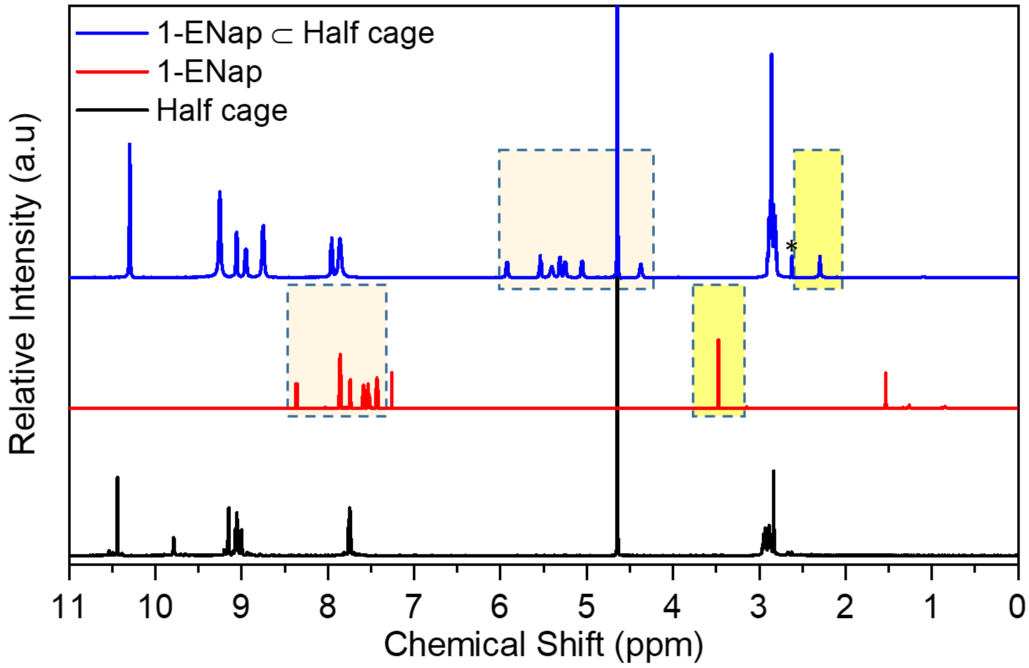

Figure S13. ^{1}H NMR spectra of 1-ENap, half cage and 1-ENap ⊂ Half Cage (2) H-G complex: ^{1}H NMR spectra of half cage (**2**) in D_2O (black), 1-ethynylnaphthalene in $CDCl_3$ (red) and 1-ENap ⊂ **2** in D_2O (blue) recorded using 600 MHz NMR instrument. Dotted regions show that guest proton peaks have up field shifted after incarceration inside half cage. The protons of 1-Ethynylnaphthalene which were in aromatic region (8.36, d, 1H), (7.86, d, 2H), (7.74, d, 1H), (7.58, t, 1H), (7.53, t, 1H), (7.43, t, 1H) have moved in up field direction (5.92, d, 1H), (5.53, t, 1H), (5.40, t, 1H), (5.31, d, 1H), (5.24, d, 1H), (5.05, d, 1H), (4.37, t, 1H) after incarceration in cage (in pale-yellow dotted region). In similar way the alkynyl proton in aliphatic region (3.47, s, 1H) also moved to (2.29, s, 1H) ppm after incarceration (in yellow dotted region).

The packing of guest molecules inside the cage induces a change in the electronic environment around the protons of cage as well. This change in the electronic environment led the change in the peak positions (chemical shift) of cage protons. So, the proton peaks which were merged in the ^{1}H NMR of free cage are separated with each in ^{1}H NMR spectra of cage after incarceration of guest molecule. There is sight change in the peak position of protons of ethylenediamine as well.

Relative peak area integration between protons of 1-ENap and half cage suggests that there is on an average two guest molecules per cage i.e., 1:2 H-G complexation between 1-ENap and half cavity.

[Asterisk (*) marked peak is some impurity from solvent]

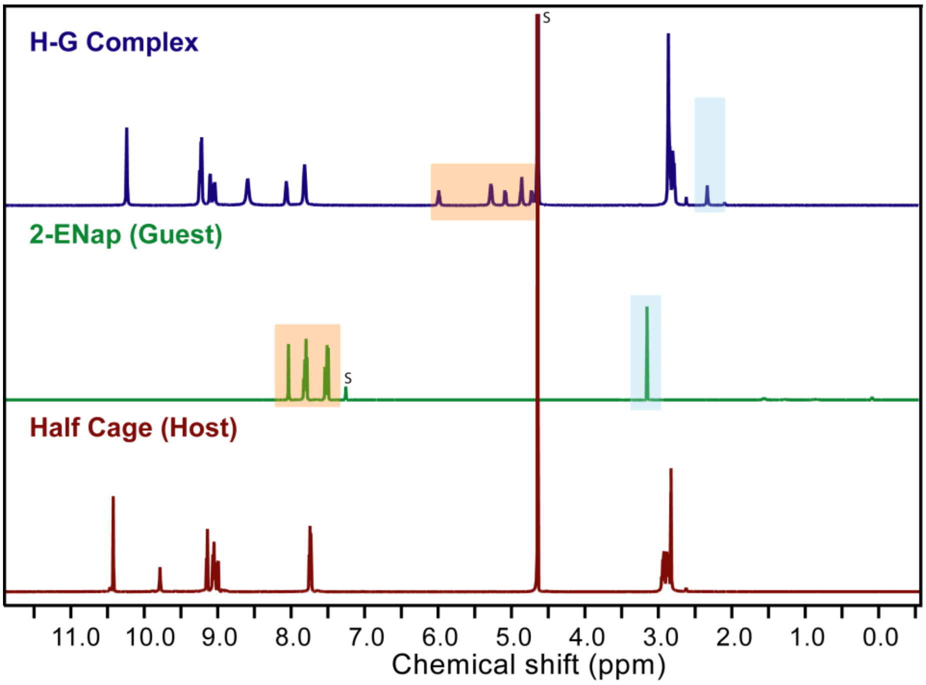

Figure S14. ^{1}H NMR spectra of 2-ENap, half cage and 2-ENap ⊂ 2 H-G complex: ^{1}H NMR spectra of 2-ENap ⊂ Half Cage H-G complex solution in navy blue colour (D_2O, 600 MHz). The upfield shifted proton peaks from guest molecule after incarceration inside half cage are highlighted in global orange colour (aromatic protons) and global blue (alkyne proton). Relative peak area integration between host and guest protons suggests that there are on an average two guest molecule per cavity (1:2 H-G complexation). ('s' marked peaks are from solvents).

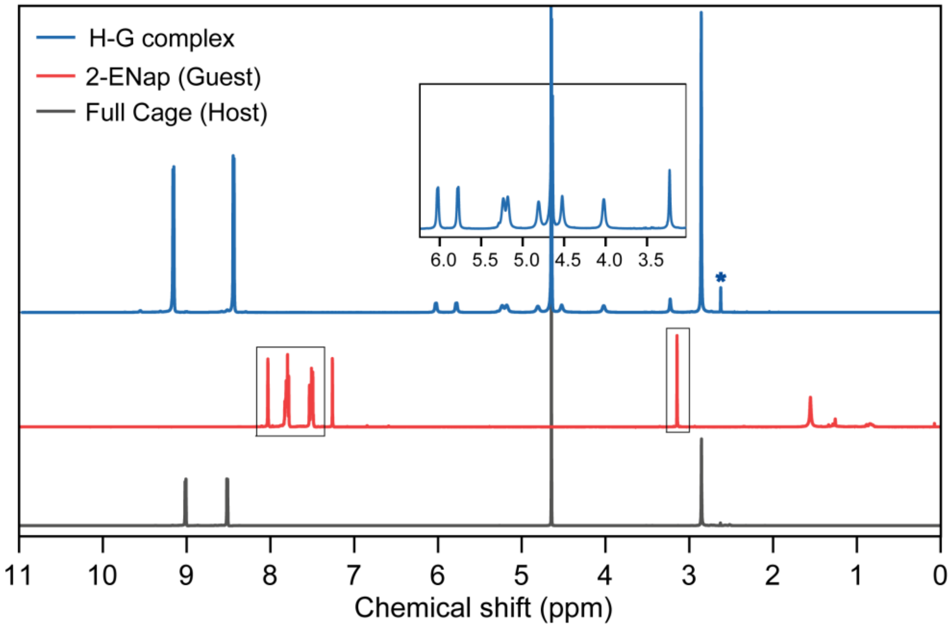

Figure S15. ^{1}H NMR spectra of 2-ENap, full cage and 2-ENap ⊂ 1 H-G complex: full cage (black, D_2O solvent), 2-ethynylnaphthalene (red, $CDCl_3$ solvent) and 2-ethynylnaphthalene ⊂ Full Cage H-G complex solution (blue spectra, D_2O solvent) using 600 MHz instrument. The upfield shifted proton peaks of guest molecule after incarceration inside full cage are shown in the inset (blue colour). Relative peak area integration between host and guest protons suggests that there are on an average four guest molecule per cavity (1:4 H-G complexation).

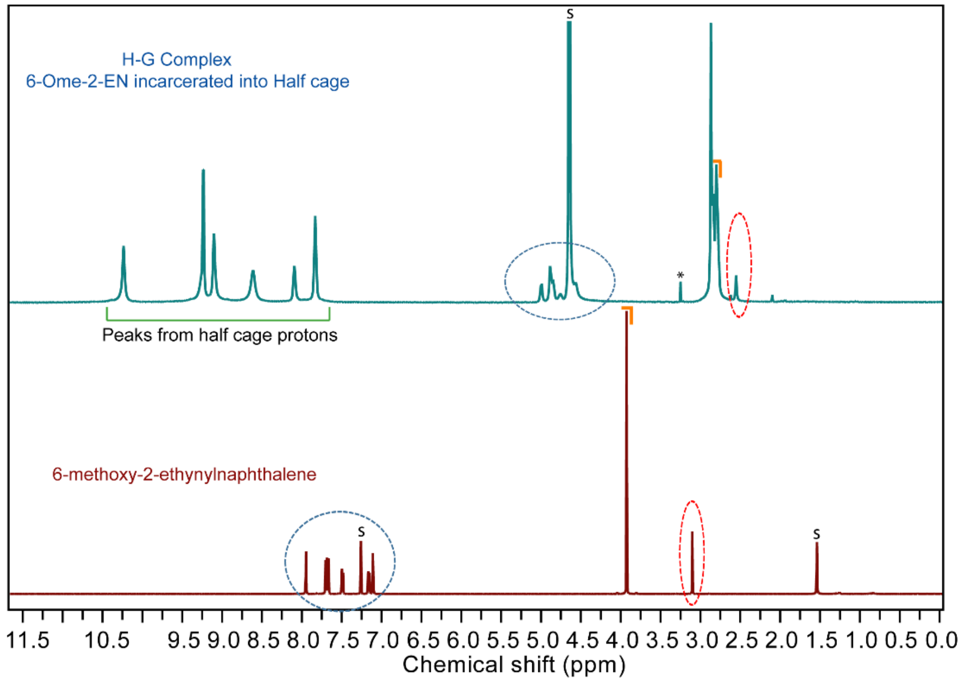

Figure S16. ^{1}H NMR spectra of 6-Ome-2-ENap ⊂ half cage (2) H-G complex: ^{1}H NMR spectra of 6-Ome-2-ENap (red, $CDCl_3$ solvent) and 6-Ome-2-ENap ⊂ **2** (green, D_2O solvent). Relative peak area integration between host and guest protons suggests that there are on an average two guest molecule per cavity. The upfield shifted proton peaks of guest molecule after incarceration inside half cage are marked with dash line (aromatic and alkyne proton) and orange colour solid line (methoxy protons).

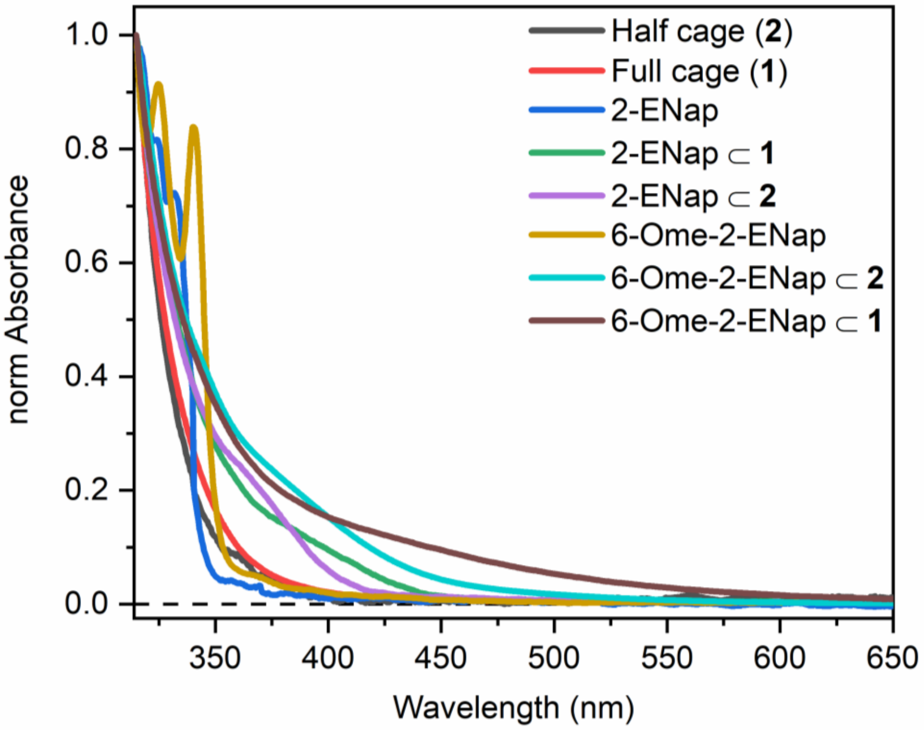

Figure S17. UV-vis absorption spectra of 2-ENap ⊂ 1/2 and 6-Ome-2-ENap ⊂ 2: UV-vis absorption spectra of host (full and half cages), guest (2-ENap and 6-Ome-2-ENap) and Host-Guest systems (2-ENap ⊂ **1**, 2-ENap ⊂ **2**, 6-Ome-2-ENap ⊂**1** & 6-Ome-2-ENap ⊂ **2**). The absorption spectra of full cage, half cage and all the H-G complex solution were taken in water. The absorption spectra of 2-ENap and 6-Ome-2ENap were taken in chloroform. All the H-G complex solutions have extra band (CT band) which was neither present in free host nor in guest molecules.

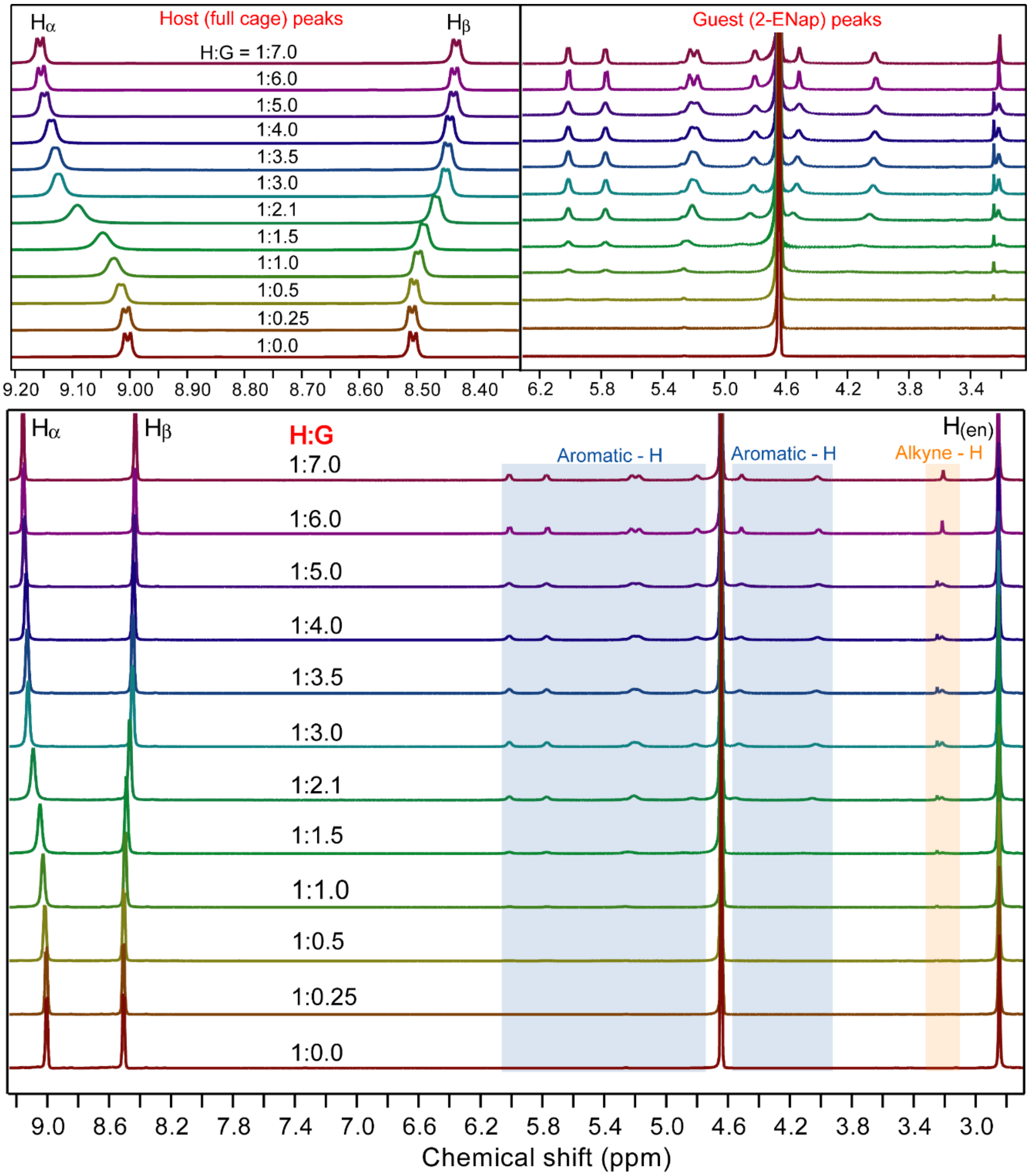

Figure S18. ^{1}H NMR spectra of 2-ENap ⊂ 1 complex with increasing amount of 2-ENap: Complete ^{1}H NMR spectra of 2-ENap ⊂ Full Cage H-G complex in the presence of increasing amounts of 2-ENap (in bottom). The zoomed in spectra in aromatic region (Top left section) clearly shows the shift in the peak position (δ_{ppm}) of H_{α} and H_{β} protons triazine core present in full cage. The zoomed in spectra (top right section) shows the upfield shifted proton peaks of guest molecules (δ 6.2 – 3.0 ppm) and their change in peak positions with increasing amount of guest loading (shown with shaded region). (*The spectra in top left and right section have different amount of zoomed in values; just for clear visualization)

This data show that initially there is shift in peak positions but after certain amount of guest concentration (4 equivalent) there is almost no shift. This indicates that almost all the cages are now filled and the maximum occupancy of full cage for 2-ENap molecule is 4, means there is on an average 4 molecules of 2-ENap per full cage.

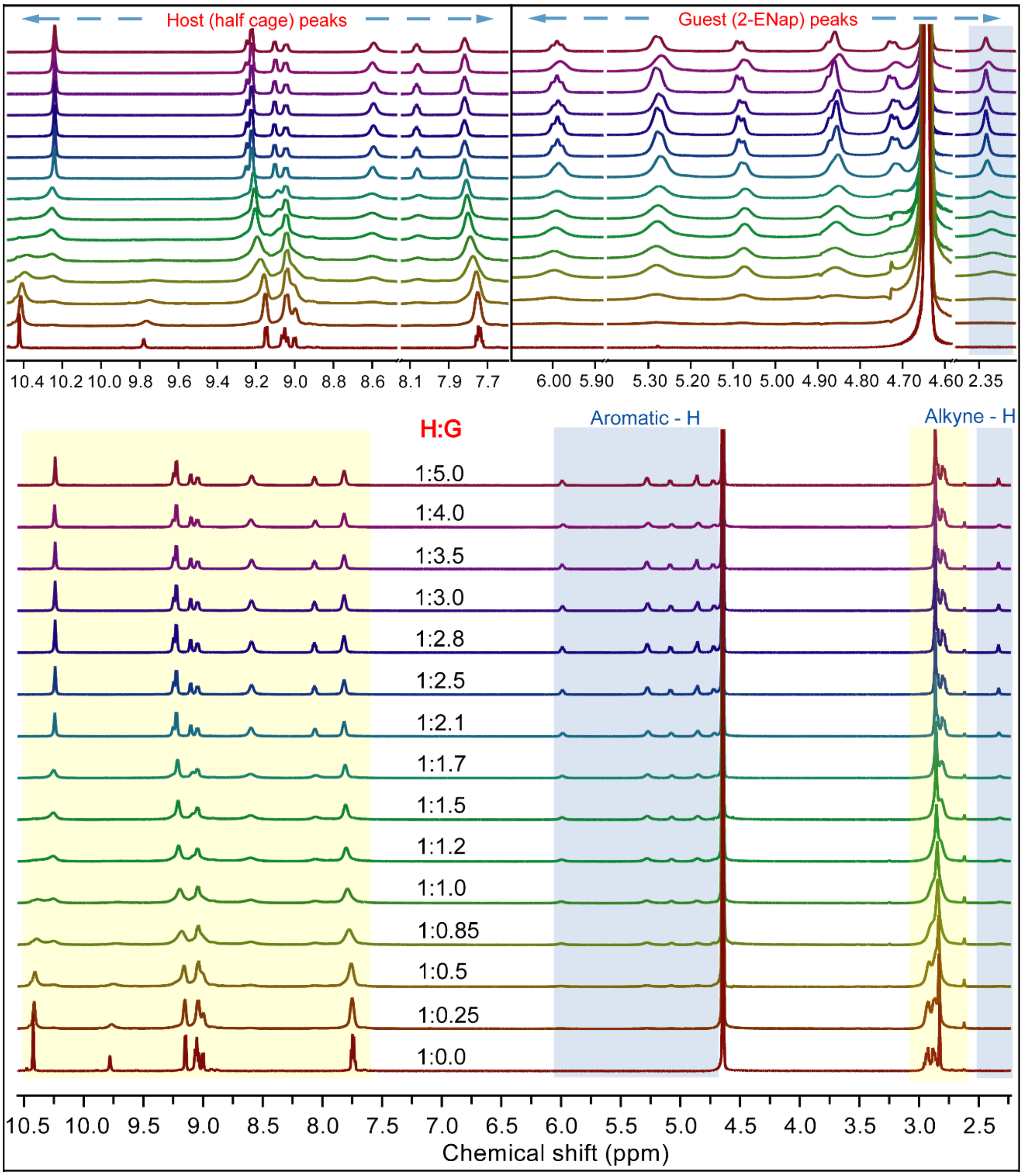

Figure S19. ^{1}H NMR spectra of 2-ENap ⊂ 2 complex with increasing amount of 2-ENap: Complete ^{1}H NMR spectra of 2-ENap ⊂ Half cage H-G complex in the presence of increasing amounts of 2-ENap (in bottom, half cage peaks in yellow shaded region and upfield shifted 2-ENap peaks in blued region). The zoomed in spectra in aromatic region (Top left section) clearly shows the shift in the peak position (δ_{ppm}) of protons from triazine core present in half cage ((δ 10.5 – 7.5 ppm). The zoomed in spectra (top right section) shows the upfield shifted proton peaks of guest molecules and their change in peak positions with increasing amount of guest loading. (*The spectra in top left and right section have different amount of zoomed in values; just for clear visualization).

This data show that initially there is shift in peak positions but after certain amount of guest concentration (2 equivalent) there is almost no shift. This indicates that almost all the cages are now filled and the maximum occupancy of half cage for 2-ENap molecule is 2, means there is on an average 2 molecules of 2-ENap per half cage.

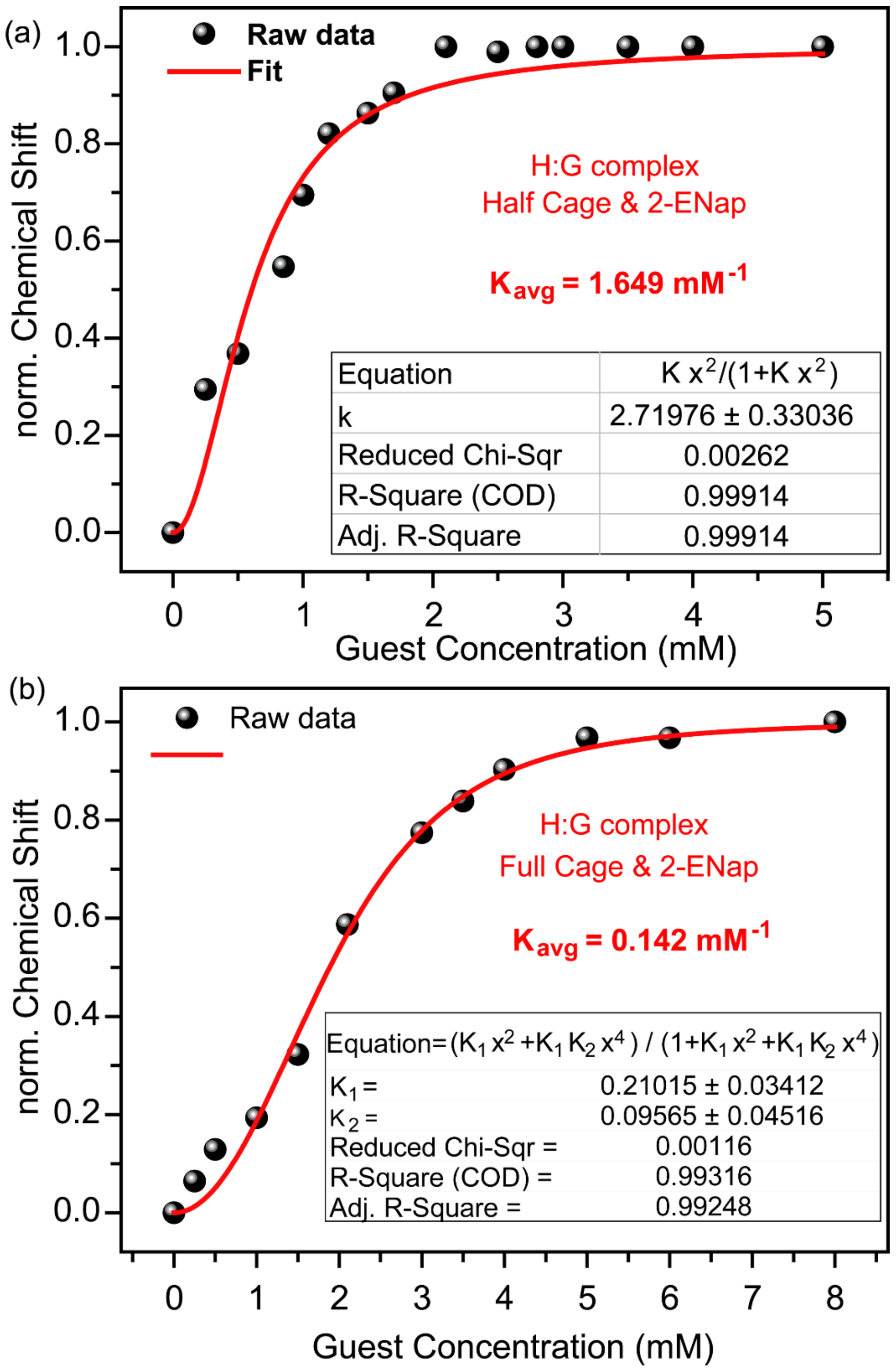

Figure S20. ^{1}H NMR-based binding curves for 2-ENap ⊂ 1 and 2-ENap ⊂ 2 complexes: ^{1}H NMR-based Binding titration curve: evolution of normalized chemical shift with increasing amount of guest concentration, (a) 2-ENap ⊂ Half Cage and (b) 2-ENap ⊂ Full Cage with their binding constants, fitting equations (models) and average binding constants (K_{avg}) in mM^{-1}. In both the cases cooperative binding process is happening and this data also show that half cage can accommodate maximum 2 and full cage can 4 molecules in their pockets. The half cage has around 10 times higher binding strength than full cage for 2-ENap molecule.

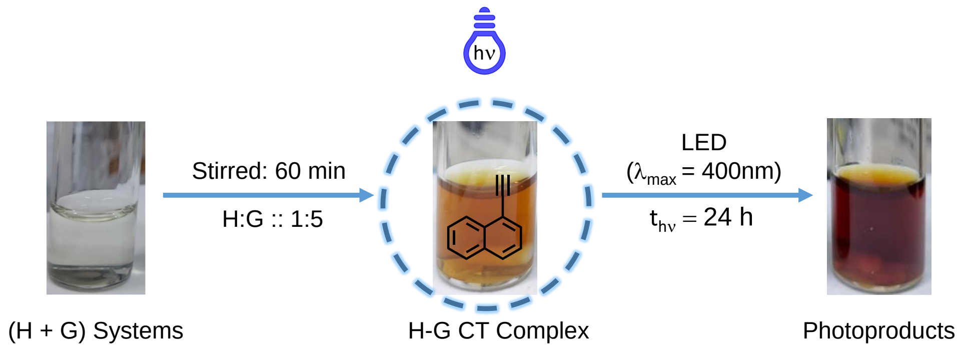

Figure S21. Photoreaction scheme of 1-ENap ⊂ 1 H-G complex with visible light: Photoreaction scheme inside full cage: from the formation of H-G CT complex to photoreaction under visible light. There is colour change in the solution after light illumination which talk about conversion of reactant to product.

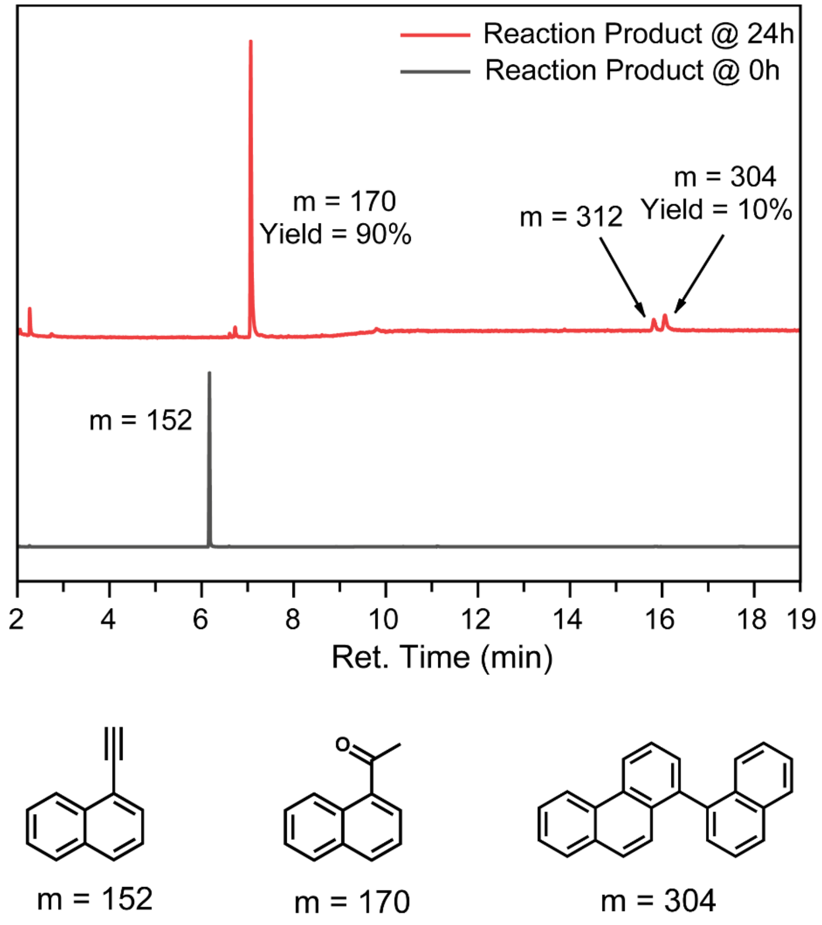

Figure S22. Photoproduct identification in photosystem 1-ENap ⊂ 1: GC-MS chromatograms of the samples before (black) and after 24 h photoreaction (red). The structure corresponding to these masses are shown below. The 1-ethynylnaphthalene (mass = 152 Da) is converted to products in 24 h of photoreaction and gives 1-acetonaphthone (m = 170 Da) as major product along with [4+2] cycloaddition product (1-(naphthalen-1-yl)phenanthrene; named as 1-Phe-1-Nap) as minor product (m = 304 Da). The relative percentage of major and minor products based on GC-MS data are 90% and 10% respectively. The mass = 312 Da is corresponds to TPT (Triazene-based ligand used in cavity).

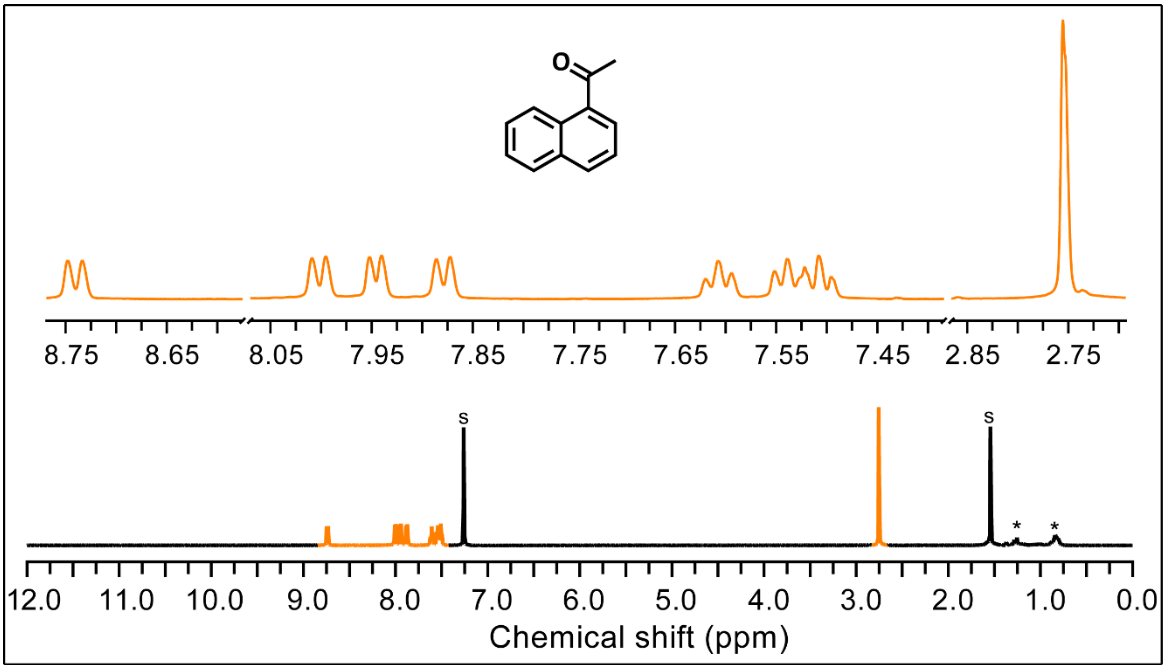

Figure S23. ^{1}H NMR spectra of oxidative product 1-acetonaphthone: ^{1}H NMR spectra of alkyne hydration (oxidation) product, 1-acetonaphthone: 600 MHz, $CDCl_3$, Room Temperature, Chemical shift(ppm), (8.74, d, 1H), (8.00, d, 1H), (7.94, d, 1H), (7.88, d, 1H), (7.61, t, 1H), (7.54, t, 1H), (7.51, t, 1H), (2.76, s, 3H). The asterisk marked peaks are coming from the impurities (like grease) present in chloroform, n-hexane and ethyl acetate solvents which were used in compound isolation and purification. Solvent peak is marked with ‘s’.

Formula	m/z	Observed m/z
$C_{24}H_{16}$	304.1244	304.1244

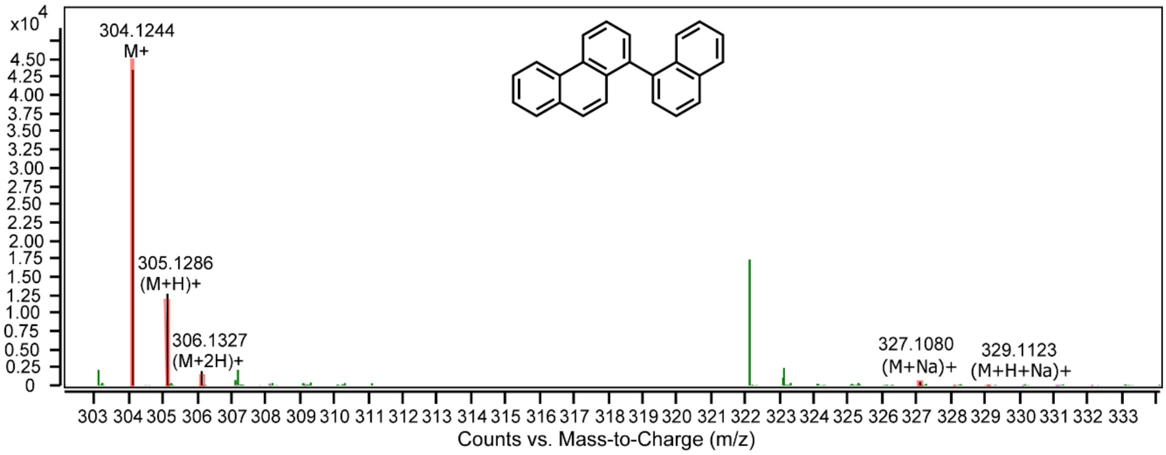

Figure S24. HR-MS chromatogram of [4+2] cycloaddition product 1-Phe-1-Nap: The chromatogram has parent mass (M+) with highest intensity along with coupling adducts with hydrogen and Sodium atoms. The Calculated m/z (304.1244 Da) and observed m/z (304.1244 Da) have excellent match up to four decimal places. This HR-MS data highly supports the formation of 1-Phe-1-Nap product.

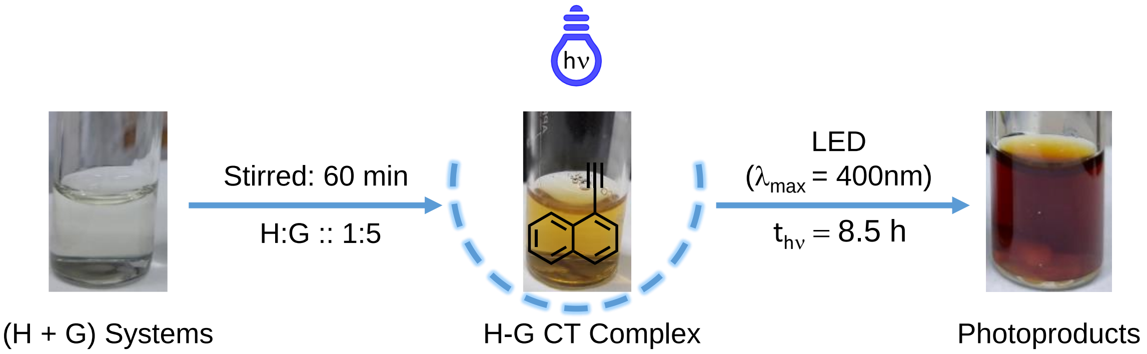

Figure S25. Photoreaction scheme of 1-ENap ⊂ 2 H-G complex with visible light: Photoreaction scheme inside half cage: from the formation of H-G CT complex to photoreaction under visible light. There is colour change in the solution after light illumination which talk about conversion of reactant to product.

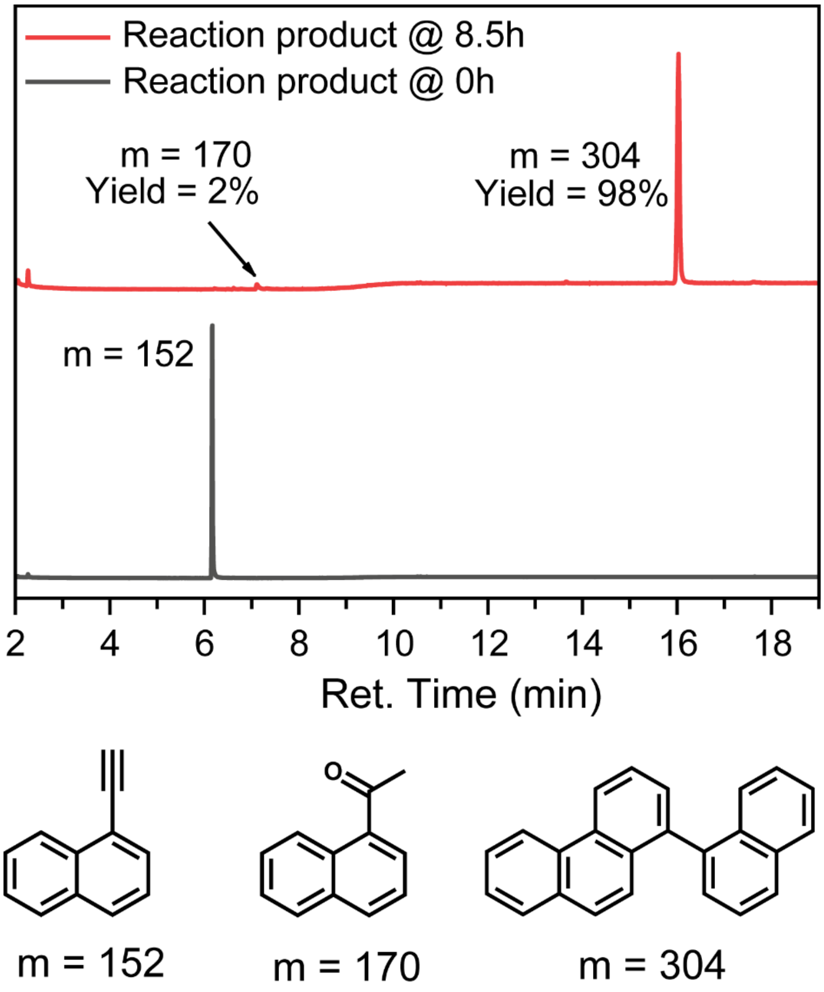

Figure S26. Photoproducts identification in photosystem 1-EN ⊂ 2: GC-MS chromatograms of the samples before (black) and after 8.5 h photoreaction (red). The structure corresponding to these masses are shown below. The 1-ethynylnaphthalene (mass = 152 Da) is converted to products in 8.5 h of photoreaction and gives [4+2] cycloaddition product (1-(naphthalen-1-yl)phenanthrene; named as 1-Phe-1-Nap) as major product (m = 304 Da) along with 1-acetonaphthone (m = 170 Da) as minor product. The relative percentage of major and minor products based on GC-MS data are 98% and 2% respectively.

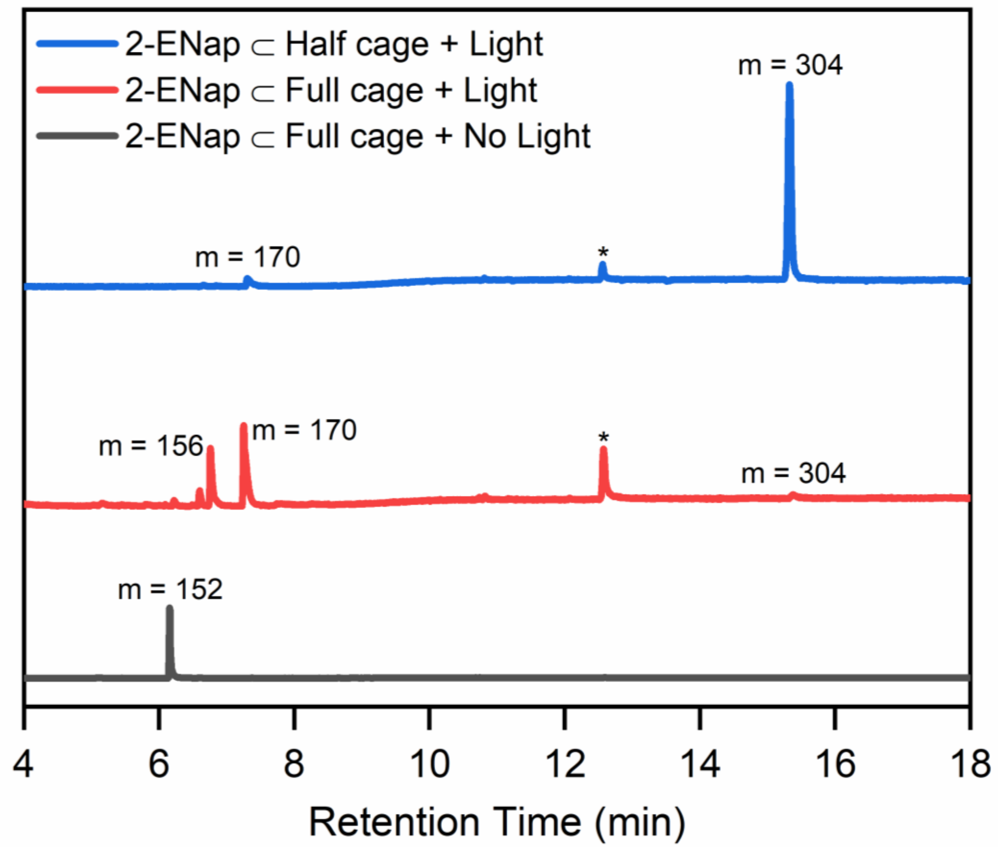

Figure S27: Photoproduct identification in photosystem 2-ENap ⊂ 1/2: GC-MS chromatograms of reaction mixture before and after photoreaction. There is only one peak corresponding to 2-ethynylanaphthalene (m = 152 Da, RT = 6.2 min.) in reaction mixture before light irradiation (reaction at 0 h, black chromatogram). From the GC-MS chromatogram it is clear that after light irradiation, [4+2] cycloaddition reaction is more selective in half cage (blue chromatogram) and oxidative reaction is more selective in full cage (red chromatogram).

The 2-ENap ⊂ Half Cage solution after 10 h of light irradiation (blue coloured chromatogram) has two peaks at RT = 15.33 min (m = 304 Da) and RT 7.30 min (m = 170 Da) with their relative percentages as 94 % and 6 % respectively.

The 2-ENap ⊂ Full Cage solution after 24 h of light irradiation (red coloured chromatogram) has four peaks at RT = 15.33 min (m = 304 Da, % = 3.5), RT 7.30 min (m = 170 Da, % = 60), RT 6.76 min (m = 156 Da, % = 33.5) and RT 6.21 min (m = 152 Da, % = 3). The structure corresponding to these masses are shown below.

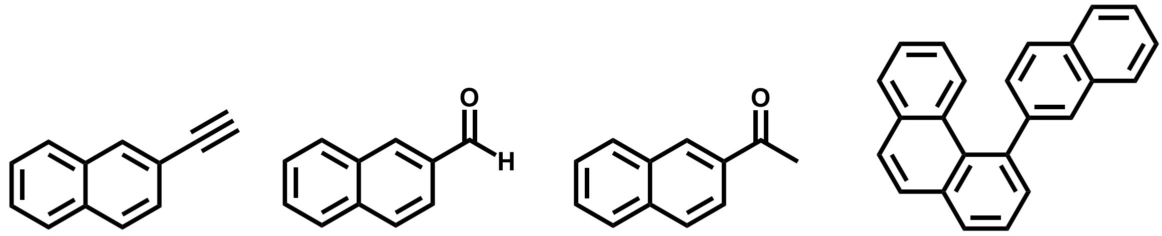

m = 152 **m = 156** **m = 170** **m = 304**

The asterisk (*) marked peaks are coming from some impurity present in GC-MS column.

Formula	m/z	Observed m/z
$C_{24}H_{16}$	304.1244	304.1244

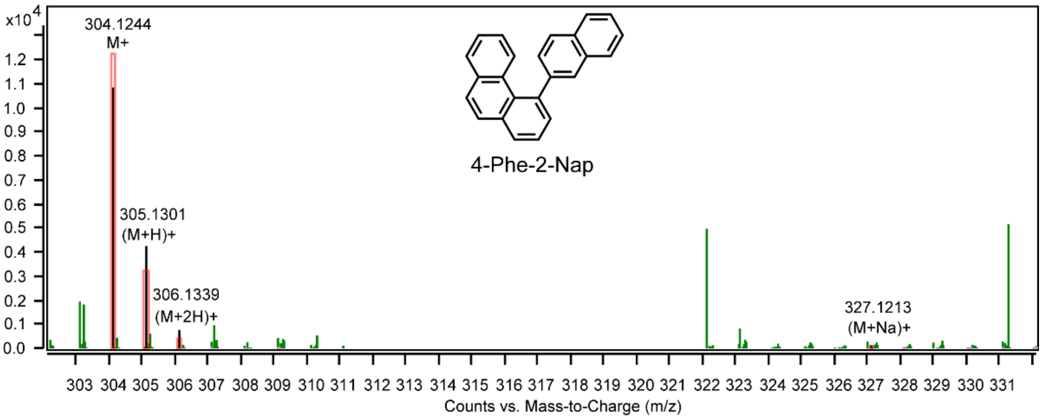

Figure S28. HR-MS chromatogram of [4+2] cycloaddition product 4-Phe-2-Nap: The HR-MS data of 4-(naphthalen-1-yl)phenanthrene; named as 4-Phe-2-Nap. The chromatogram has parent mass (M+) with highest intensity along with coupling adducts with hydrogen and Sodium atoms. The Calculated m/z (304.1244 Da) and observed m/z (304.1244 Da) have excellent match up to four decimal places. The HR-MS data highly supports to the formation of 4-Phe-2-Nap product.

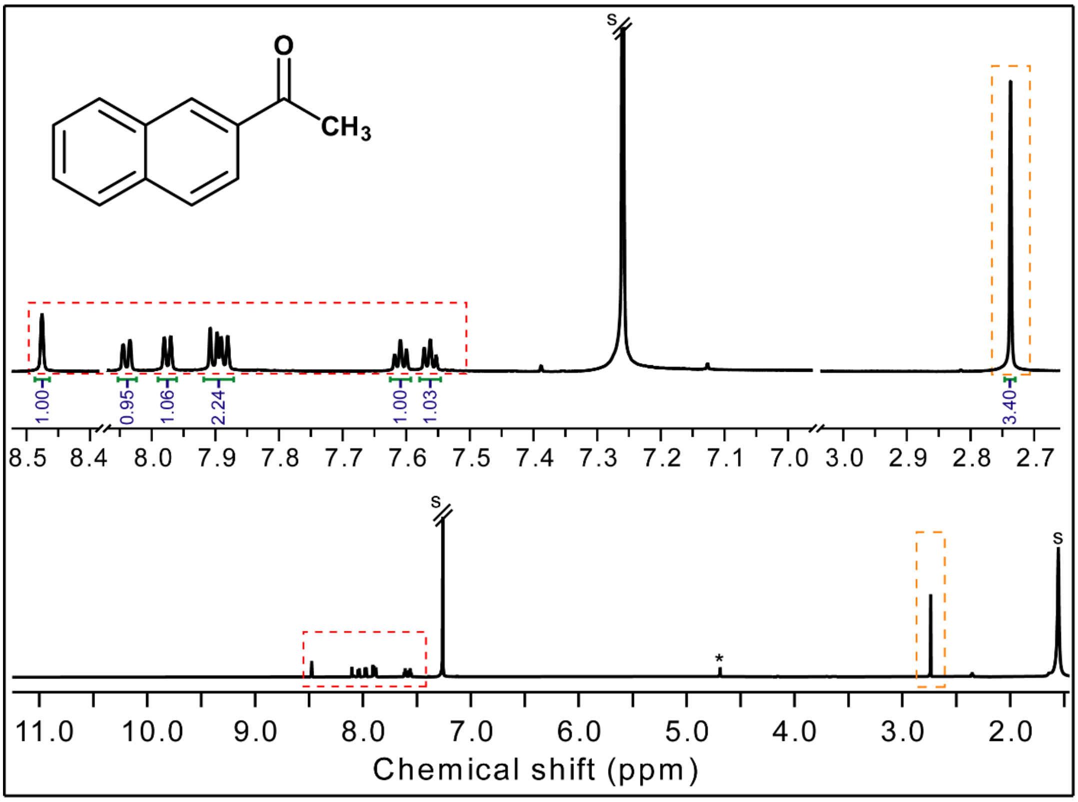

Figure S29. ¹H NMR spectra of oxidative product 2-acetonaphthone: ¹H NMR spectra of alkyne hydration (oxidation) product, 2-Acetonaphthone from 2-ENap: 800 MHz, $CDCl_3$, Room Temperature, Chemical shift(ppm), (8.48, s, 1H), (8.04, d, 1H), (7.98, d, 1H), (7.90, d, 1H), (7.89, d, 1H), (7.61, t, 1H), (7.56, t, 1H), (2.74, s, 3H). The asterisk marked peaks are coming from the impurities (like grease) present in chloroform, n-hexane and ethyl acetate solvents which were used in compound isolation and purification. Solvent peak is marked with ‘s’. All the peaks corresponding to 2-acetonaphones are present in recorded proton NMR spectra along with their multiplicities which confirms the formation of 2-acetonaphthone during the reaction.

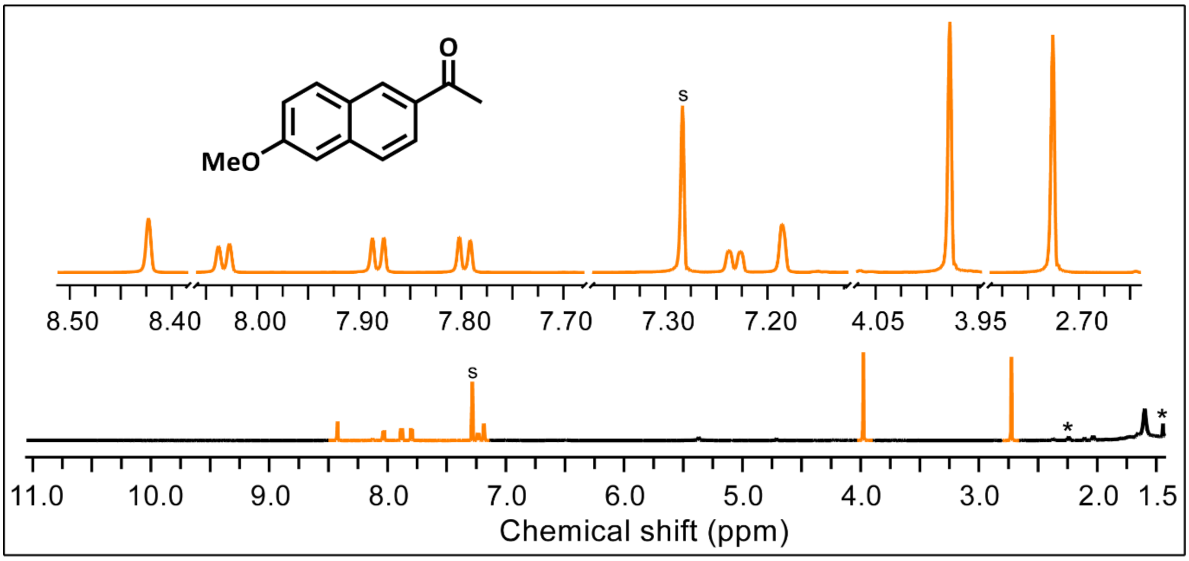

Figure S30. ^{1}H NMR spectra of oxidative product 6-methoxy-2-acetonaphthone: ^{1}H NMR spectra of alkyne hydration (oxidation) product, 6-methoxy-2-acetonaphthone from 6-Ome-2-ENap: 800 MHz, $CDCl_3$, Room Temperature, Chemical shift(ppm), (8.42, s, 1H), (8.03, d, 1H), (7.88, d, 1H), (7.79, d, 1H), (7.23, d, 1H), (7.19, s, 1H), (3.98, s, 3H), (2.73, s, 3H). The asterisk marked peaks are coming from the impurities (like grease) present in chloroform, n-hexane and ethyl acetate solvents which were used compound isolation and purification. Solvent peak is marked with ‘s’. All the peaks corresponding to 6-methoxy-2-acetonaphones are present in recorded proton NMR spectra along with their multiplicities which confirms the formation of 6-methoxy-2-acetonaphthone during the reaction.

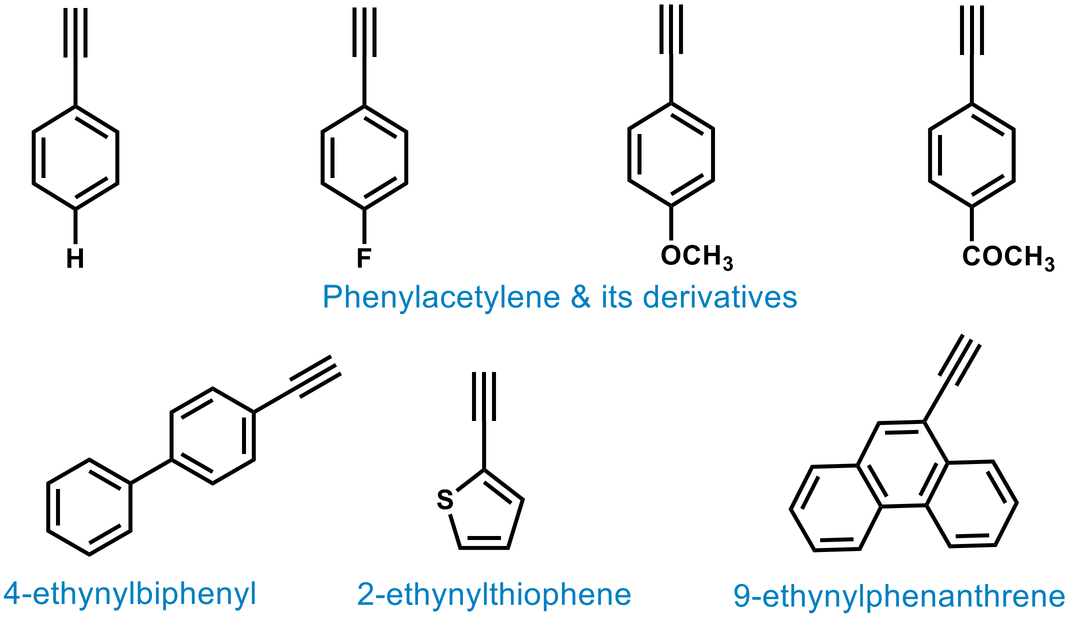

Figure S31. Structure of substrates attempted for [4+2] cycloaddition inside half cage (2): The structures of aromatic ring containing molecules with mixed ene-yne frameworks that were tested for the [4+2] cycloaddition reaction inside half cage (**2**). In these cases, the alkyne hydration product was observed as the major product in photoreactions. These molecules failed to undergo the [4+2] cycloaddition reaction inside the half cage either due to unoptimized packing of the substrate molecules within the cavity pocket which is possible in cases of phenylacetylene and its derivatives or due to the larger size of the substrate molecules, such as 9-ethynylphenanthrene, which does not allow the packing of two molecules in the close-proximity inside cavity pocket.

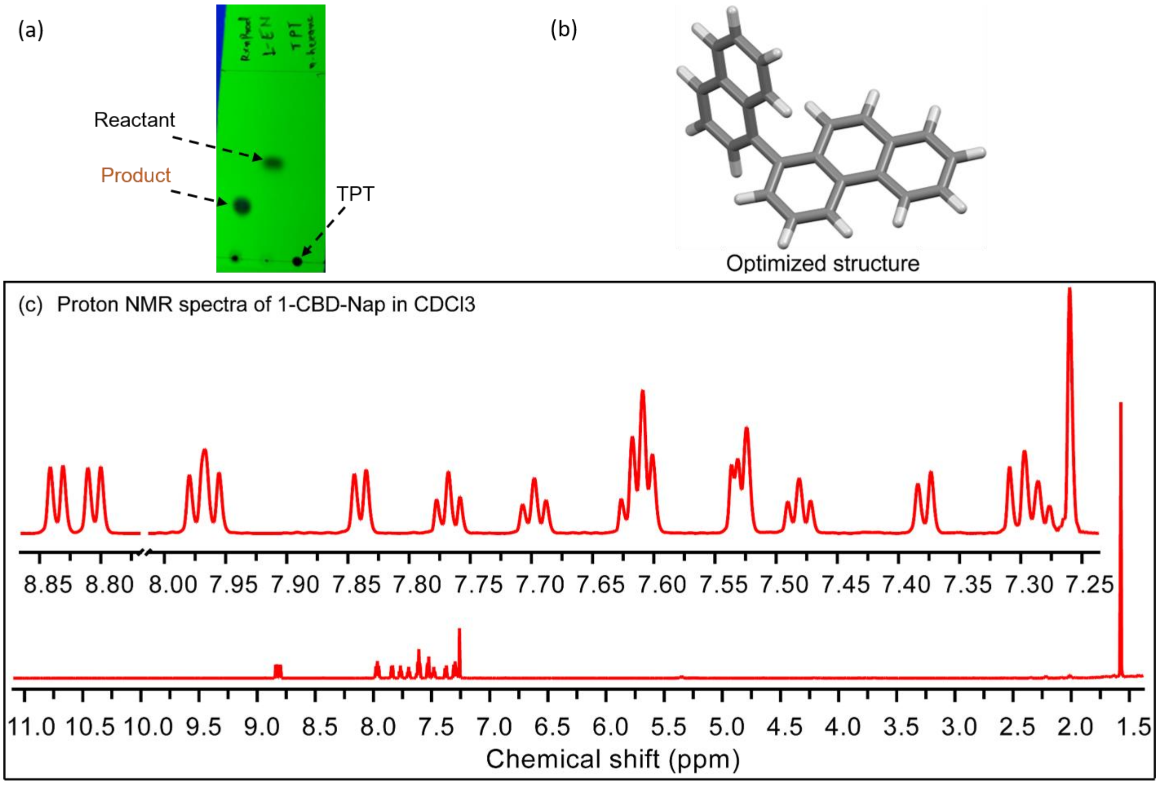

Figure S32. ^{1}H NMR spectra of [4+2] cycloaddition product 1-Phe-1-Nap: The image of TLC plate (a); where the [4+2] cycloaddition product (1-(naphthalen-1-yl)phenanthrene; named as 1-Phe-1-Nap), reactant (1-ENap) and TPT ligand molecule spots are mentioned. The optimized structure of the cycloaddition product molecule (b); shows that there is asymmetry in the structure. ^{1}H NMR spectra of cycloaddition product 1-Phe-1-Nap recorded in $CDCl_3$ solvent using 800 MHz NMR instrument (c); due to the asymmetry in structure it has NMR signal corresponding to each proton present in it. Aromatic region of spectra is zoomed in and shown in the inset. Spectra have few multiplets in between the chemical shift values of 7.925 – 8.00 ppm, 7.575 – 7.65 ppm, 7.50 – 7.55 ppm and 7.275 - 7.325 ppm where the exact multiplicity of individual protons present in those multiplets are not clear. To find the exact number of proton nuclei and their multiplicity we took the help of HSQC and COSY NMR techniques.

^{1}H NMR Data: 800 MHz, $CDCl_3$, Room Temperature, Chemical shift(ppm), (8.84, d, 1H), (8.80, d, 1H), (7.925 – 8.00, m, 2H), (7.84, d, 1H), (7.77, t, 1H), (7.70, t, 1H), (7.575 – 7.65, m, 3H), (7.50 – 7.55, m, 2H), (7.44, t, 1H), (7.38, d, 1H), (7.275 - 7.325, m, 2H)

The obtained ^{1}H NMR spectra of reaction product (1-Phe-1-Nap) is similar to the reported spectra in following reports:

Tagliatesta and co-workers[21] (*New J. Chem*. **2008**, *32*, 1847–1849).

Liu and co-workers[22] (*Helv. Chim. Acta* **2021**, *104*, e2100056)

For better understanding and clear visualization of obtained ^{1}H NMR spectra it is resolved with the help of HSQC and COSY NMR techniques.

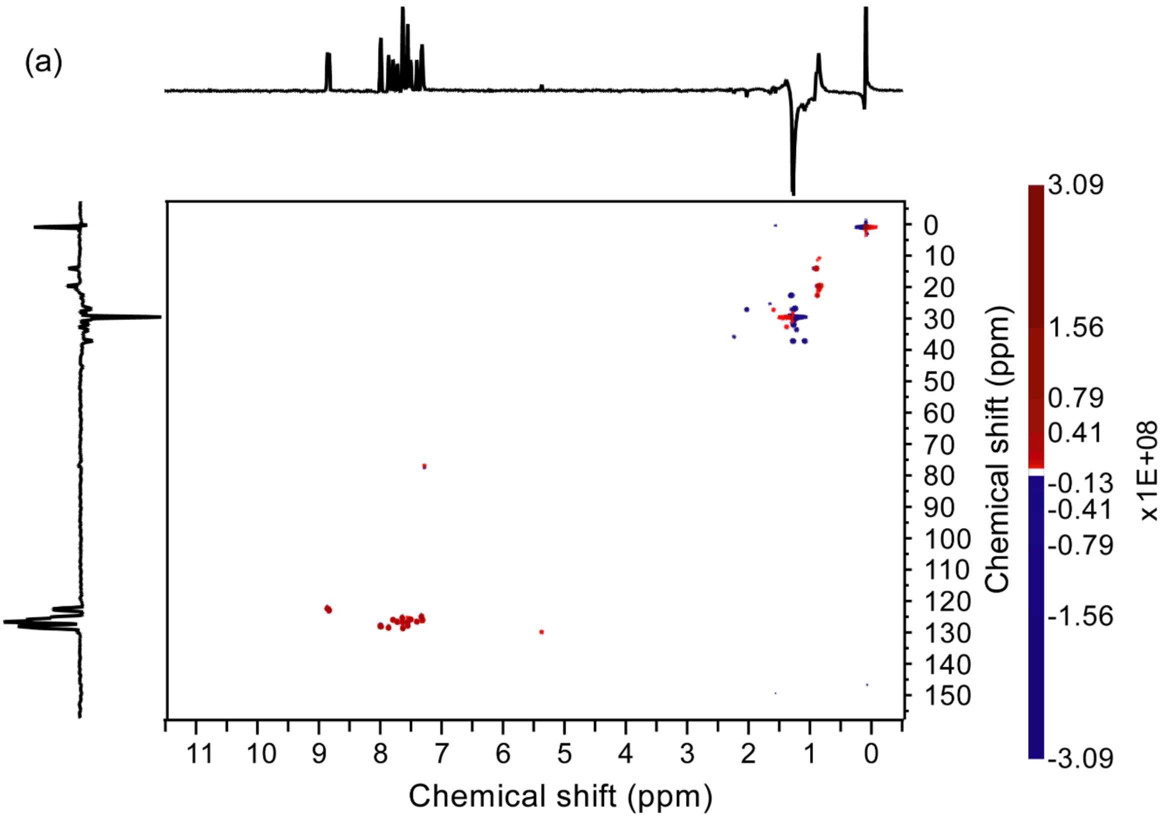

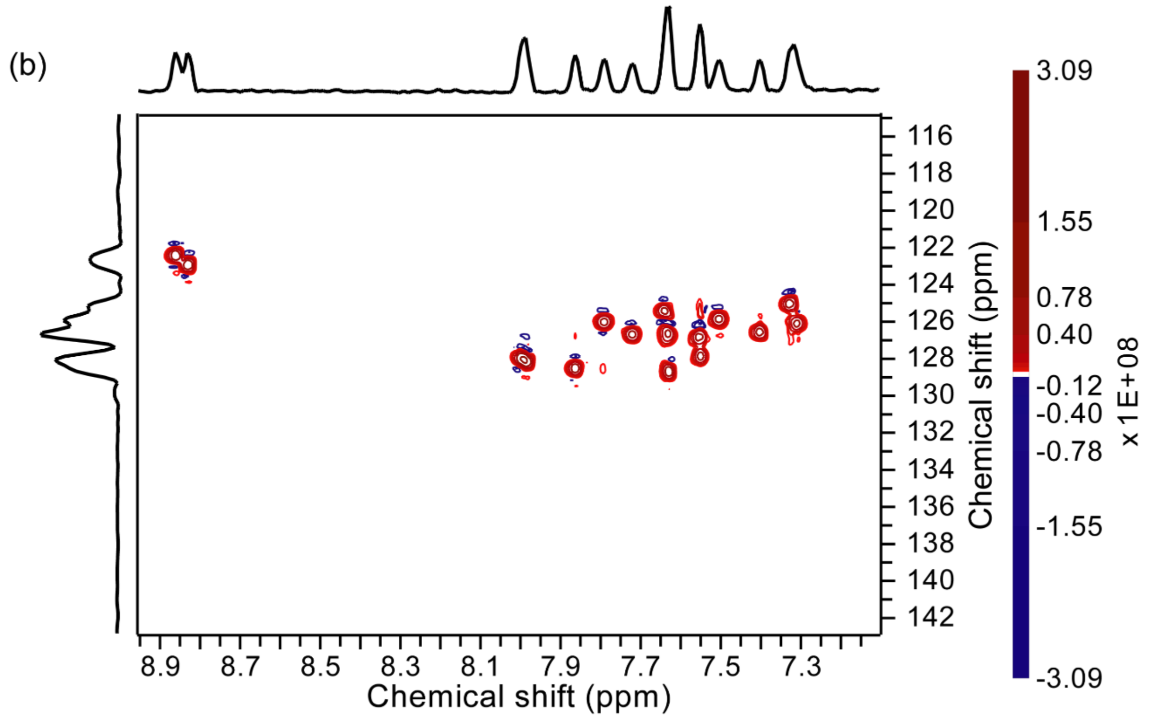

Figure S33. 2D HSQC spectra of [4+2] cycloaddition product 1-Phe-1-Nap: 2-Dimentional heteronuclear single quantum correlation (HSQC) spectra of [4+2] cycloaddition product 1-Phe-1-Nap recorded in $CDCl_3$ solvent using 800 MHz NMR instrument. Full HSQC spectra (a) whereas its zoomed in version in the aromatic region (b); which shows the correlation between chemical shift of a carbon atom with attached proton. The hetero-correlation gives the number of distinct carbon hydrogen interaction present in the system.

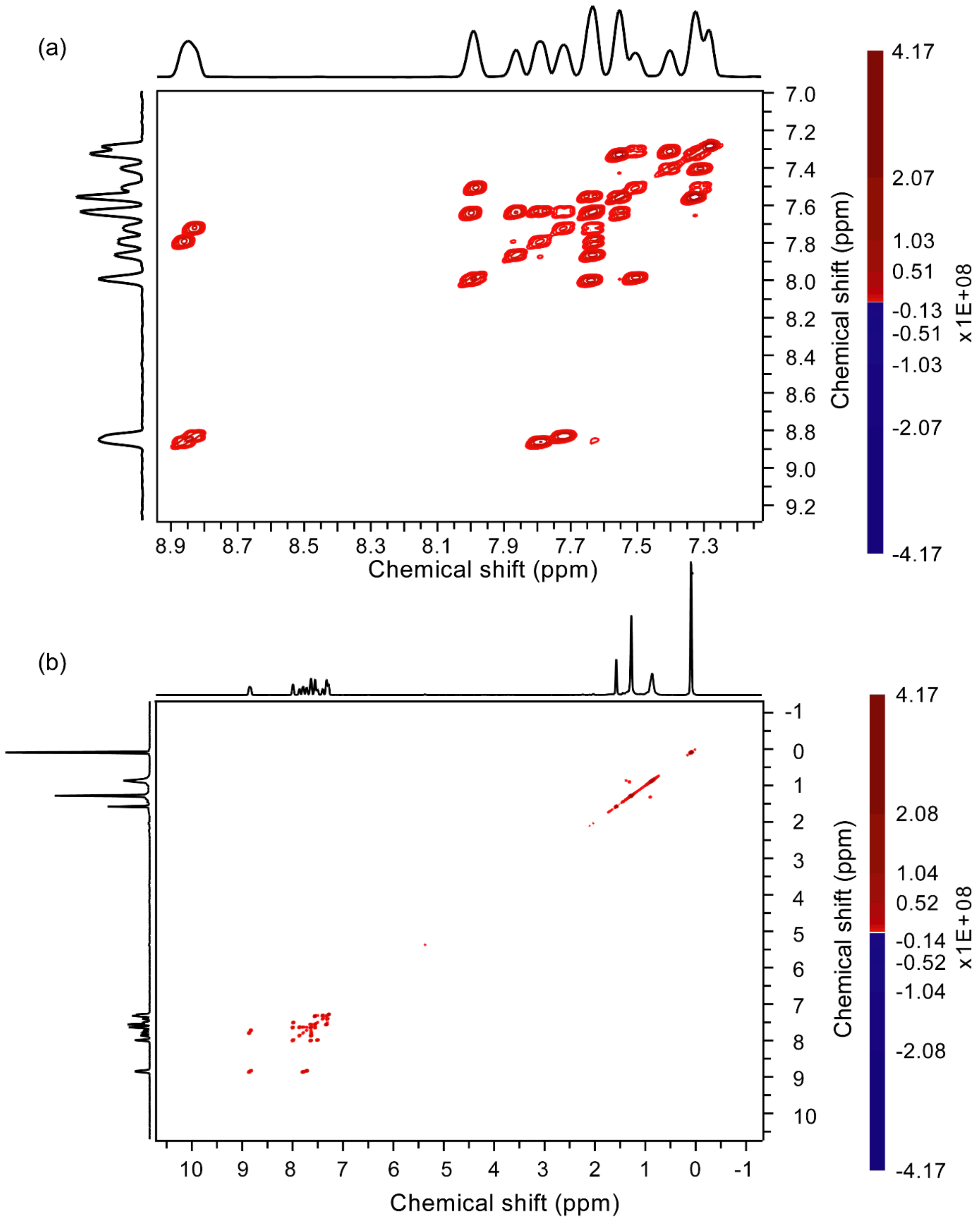

Figure S34. 2D COSY spectra of [4+2] cycloaddition product 1-Phe-1-Nap: 2-Dimentional cross correlation spectra of [4+2] cycloaddition product 1-Phe-1-Nap recorded in $CDCl_3$ solvent using 800 MHz NMR instrument. The 2D COSY data shows the through bond coupling between different protons present at adjacent carbon centres (coupling up to three bonds distance). The zoomed in version of spectra in aromatic region (a) shows the coupling between different protons present in the system. The coupling data gives the idea about the multiplicity of a particular proton; a proton is coupled with how many other distinct protons. The final correlation diagram with coupling pattern and peak multiplicity for this system based on 2D COSY and HSQC data is shown in Figure S35**.**

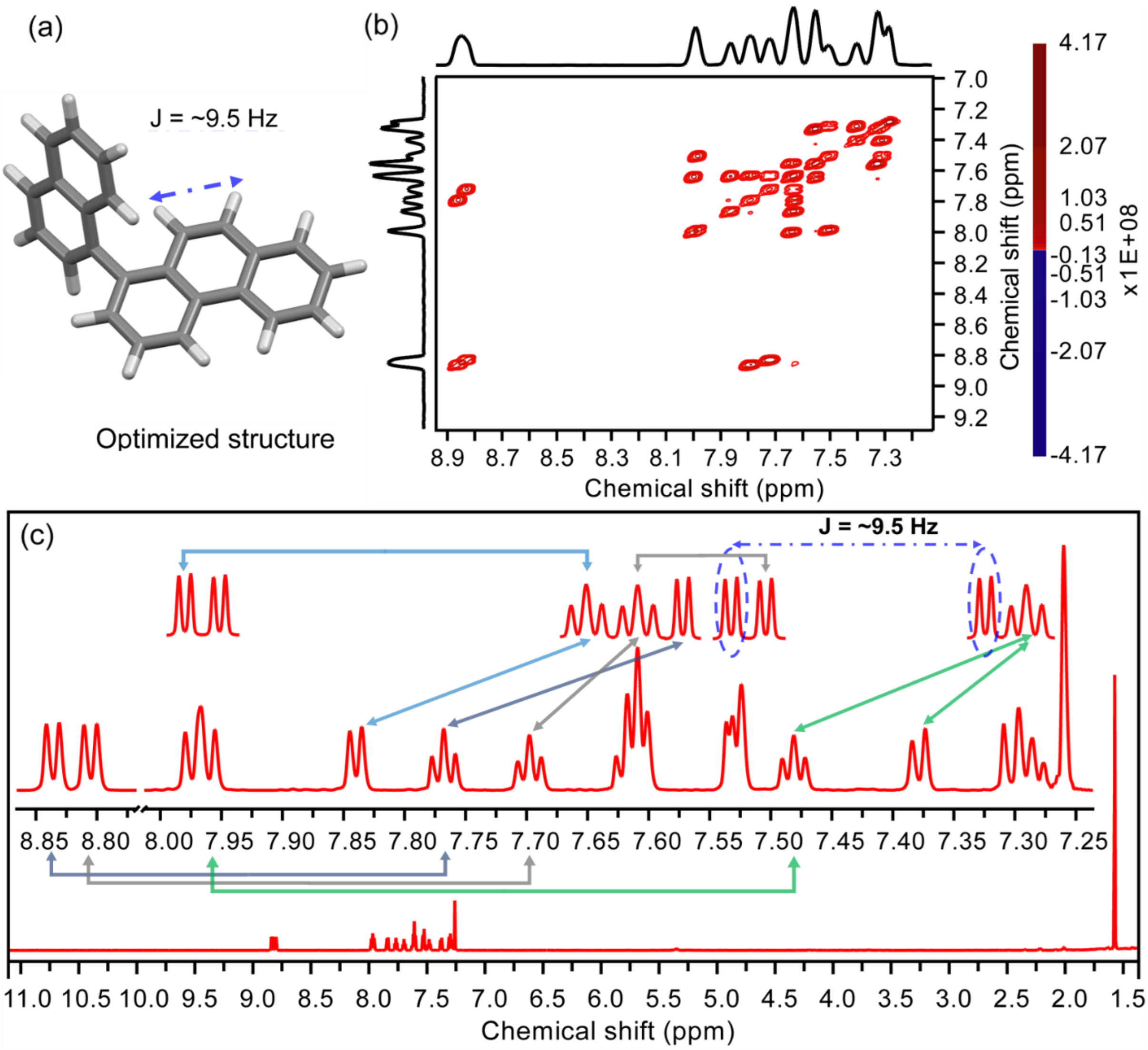

Figure S35. ^{1}H NMR spectra of 1-Phe-1-Nap with multiplicities and coupling pattern: (a) The optimized structure of the cycloaddition product molecule, (b) 2D COSY correlation spectra in aromatic region and (c) ^{1}H NMR spectra of [4+2] cycloaddition product 1-Phe-1-Nap ($CDCl_3$ solvent, 800 MHz instrument). There are some protons which have very similar chemical shift value so their NMR peaks are merged with each. These peaks are identified with the help of HSQC and COSY data. The HSQC data provided the idea about the number of proton nuclei present in a particular NMR peak and the COSY data helped in finding peak multiplicity and their coupling pattern. The final correlation diagram is drawn with the help of HSQC and COSY data which shows resolved-individual peaks with their multiplicity above their respective multiplet signals (for simple visualization and representation) and coupling patterns are shown using coloured double-headed arrows (as schematic representation of coupling). The coupling between two protons present in central ring of phenanthrene moiety is shown in blue dotted region. After finding peak multiplicity corresponding to each proton present in the structure here is the ^{1}H NMR data with its protons and their individual multiplicity.

^{1}H NMR Data: 800 MHz, $CDCl_3$, Room Temperature, Chemical shift(ppm), (8.84, d, 1H), (8.80, d, 1H), (7.925 - 8.00, 2d, 2H), (7.84, d, 1H), (7.77, t, 1H), (7.70, t, 1H), (7.575 - 7.65, 2t, 1d, 3H), (7.50 - 7.55, 2d, 2H), (7.44, t, 1H), (7.38, d, 1H), (7.275 - 7.325, 1d, 1t, 2H).

The multi-plate which is present from 7.925 – 8.00 ppm actually consist of 2 doublets similarly the multi-plate from 7.575 – 7.65 ppm consist of 2 triplets and 1 doublet, multi-plate from 7.50 – 7.55 ppm consist of 2 doublets, and multi-plate from 7.275 - 7.325 ppm consist of 1 triplet and 1 doublet.

As we go with asymmetry of structure there would be following type of coupling patterns: 2 sets of d-t-d and 2 sets of d-t-t-d coupling sequences from structure along with 1 set of d-d from central ring of phenanthrene moiety. After resolving ^{1}H NMR spectra with the help of HSQC and COSY NMR spectra we could see similar pattern in the recorded NMR which fully supports the presence of 1-Phe-1-Nap structure.

The resolved ^{1}H NMR spectra of reaction product has clear coupling patterns as expected from the 1-Phe-1-Nap structure and its similarity with Tagliatesta and co-workres report[21] (*New J. Chem*. **2008**, *32*, 1847–1849) and Liu and co-workers report[22] (*Helv. Chim. Acta* **2021**, *104*, e2100056) confirms the formation of 1-Phe-1-Nap cycloaddition product.

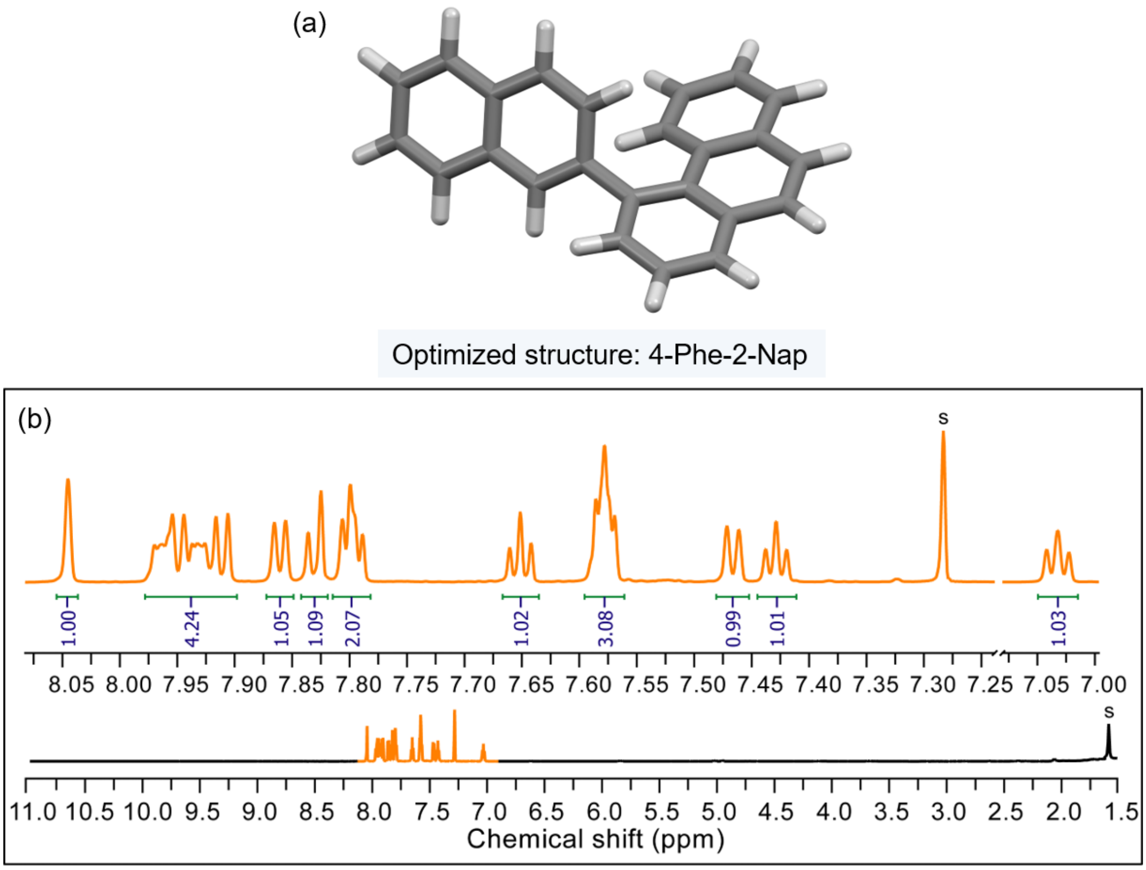

Figure S36. ^{1}H NMR spectra of [4+2] cycloaddition product 4-Phe-2-Nap: (a) Optimized structure of 4-Phe-2-Nap [4-(naphthalen-1-yl)phenanthrene]: shows that the structure is asymmetric. (b) ^{1}H NMR spectra of cycloaddition product 4-Phe-2-Nap recorded in $CDCl_3$ solvent using 800 MHz NMR instrument; due to the asymmetry in structure it has NMR signal corresponding to each proton present in it. Aromatic region of spectra is zoomed in and shown in the inset (in orange colour). The spectra have few multiplets in between the chemical shift values of 7.95 – 7.88 ppm, 7.79 – 7.76 ppm and 7.57 – 7.54 ppm where the exact multiplicity of individual protons present in those multiplets are not clear.

^{1}H NMR Data: 800 MHz, $CDCl_3$, Room Temperature, Chemical shift(ppm), (8.02, s, 1H), (7.95 - 7.88, m, 4H), (7.84, d, 1H), (7.81, d, 1H), (7.79 - 7.76, m, 2H), (7.63, t, 1H), (7.57 - 7.54, m, 3H), (7.44, d, 1H), (7.40, t, 1H), (7.01, t, 1H). Solvent peaks are marked with ’s’.

The obtained ^{1}H NMR spectra of reaction product (4-Phe-2-Nap) is similar to the spectra reported in following reports: Tagliatesta and co-workers[21] (*New J. Chem*. **2008**, *32*, 1847–1849), Liu and co-workers[22] (*Helv. Chim. Acta* **2021**, *104*, e2100056) and Fleming and co-workers[23] (*Angew. Chem. Int. Ed.* **2016**, *55*, 14770 –14773).

This confirms the formation of 4-Phe-2-Nap cycloaddition product during photoreaction.

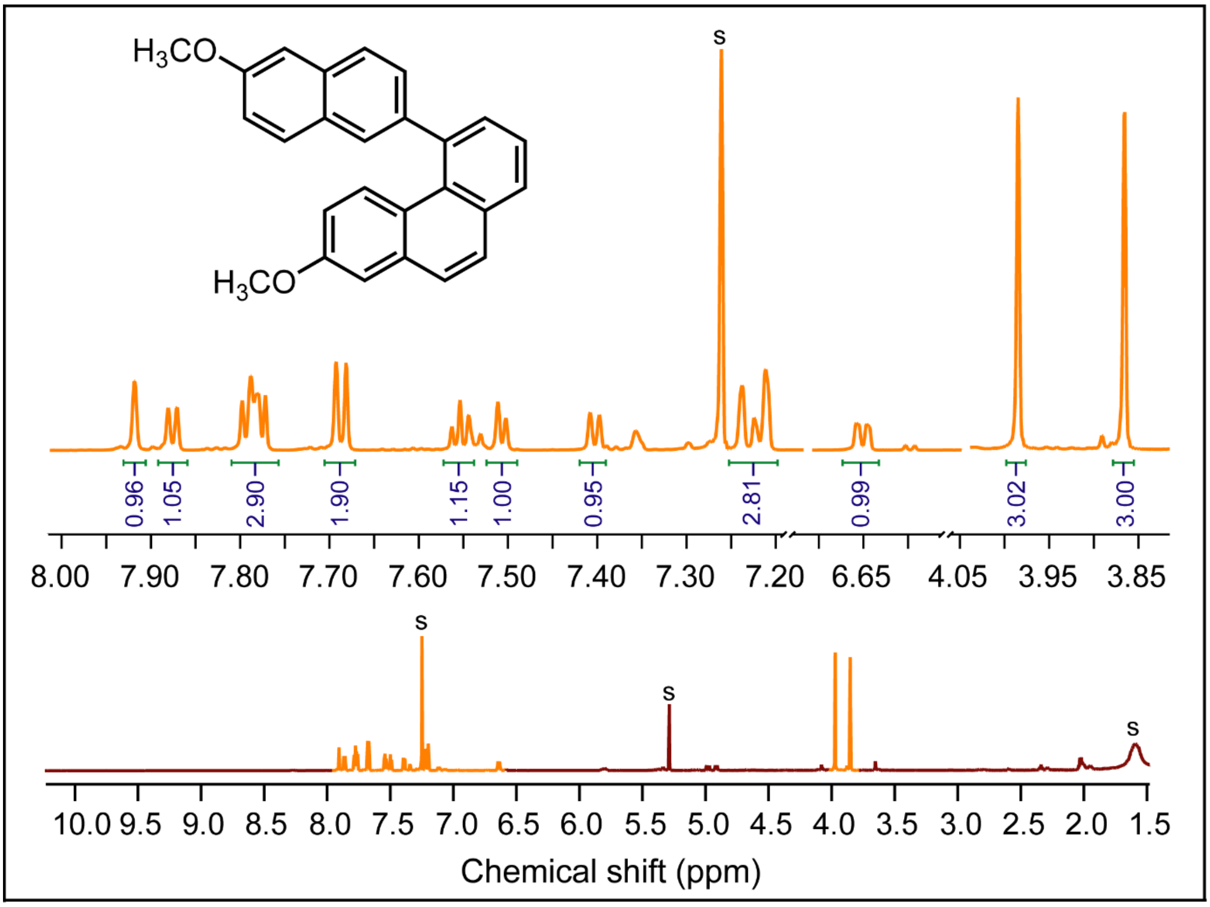

Figure S37. ^{1}H NMR spectra of [4+2] cycloaddition product 2,6'-di-Ome-5-Phe-2-Nap: ^{1}H NMR spectra of [4+2] cycloaddition product 2-methoxy-5-(6-methoxynaphthalen-2-yl)phenanthrene [Named as: 2,6'-di-Ome-5-Phe-2-Nap].

^{1}H NMR Data: 800 MHz, $CDCl_3$, Room Temperature, Chemical shift (ppm), (7.92, s, 1H), (7.87, d, 1H), (7.81 - 7.76, m, 3H), (7.70 - 7.67, m, 2H), (7.55, t, 1H), (7.51, d, 1H), (7.40, d, 1H), (7.25 - 7.20, m, 3H), (6.65, d, 1H).

Relative peak area integration shows that the required number of proton peaks are present with few multiplets. To resolve the multiplet peak and know the coupling pattern 2D COSY and HSQC data was recorded which is shown in next figures.

‘s’ marked peaks are from solvent impurities; Around 7.26 ppm and 1.55 ppm are from chloroform and peak around 5.32 ppm is from dichloromethane.

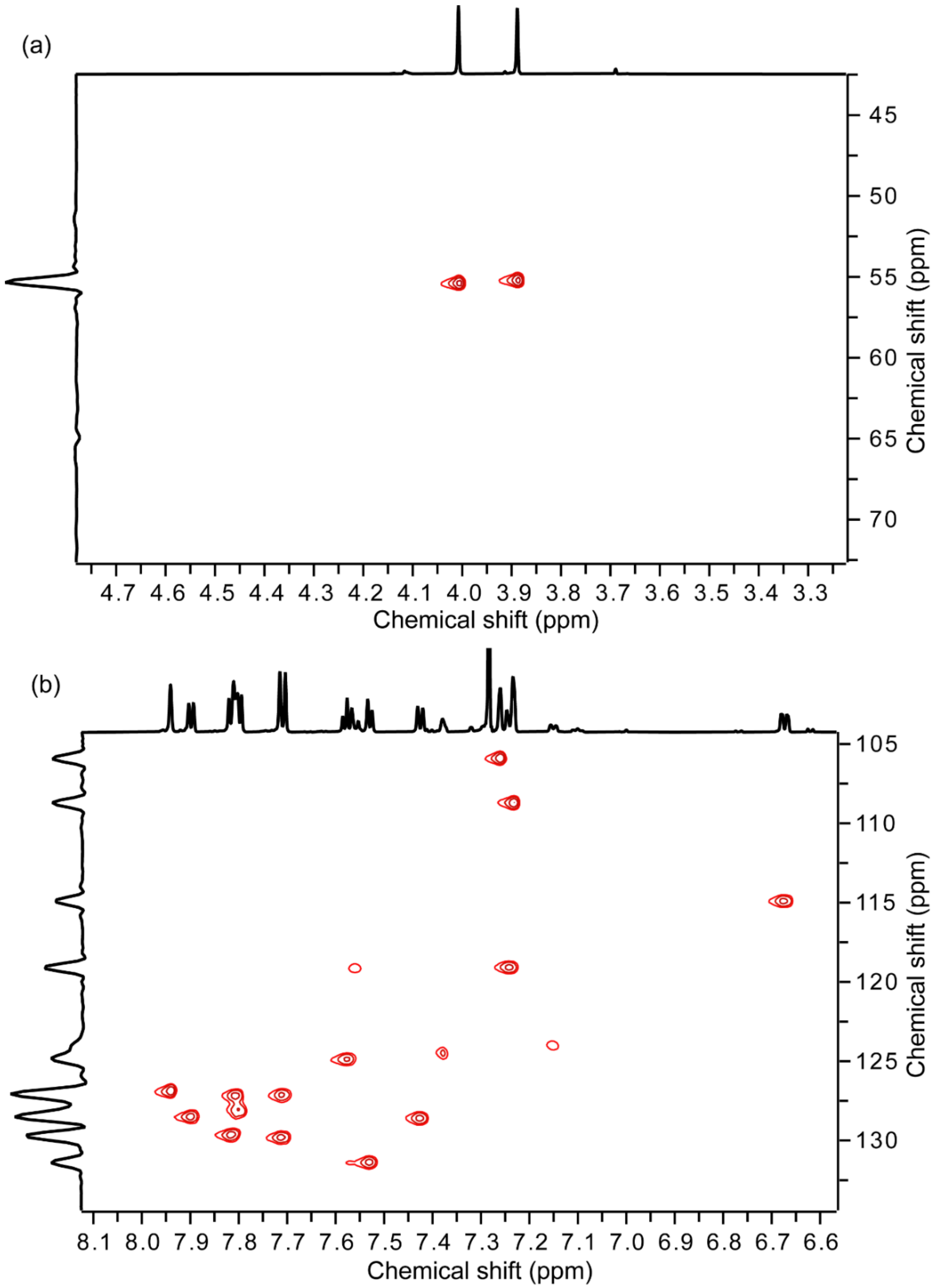

Figure S38. 2D HSQC spectra of [4+2] cycloaddition product 2,6'-di-Ome-5-Phe-2-Nap: The HSQC spectra (800 MHz instrument, Room temperature, $CDCl_3$ solvent) of [4+2] cycloaddition product 2-methoxy-5-(6-methoxynaphthalen-2-yl)phenanthrene [Named as: 2,6'-di-Ome-5-Phe-2-Nap] in aliphatic region (a) and aromatic region (b). The 14 different C-H interaction can be seen in aromatic region and 2 different CH_3 interaction in aliphatic regions which is satisfying the structural criteria for 2,6'-di-Ome-5-Phe-2-Nap product.

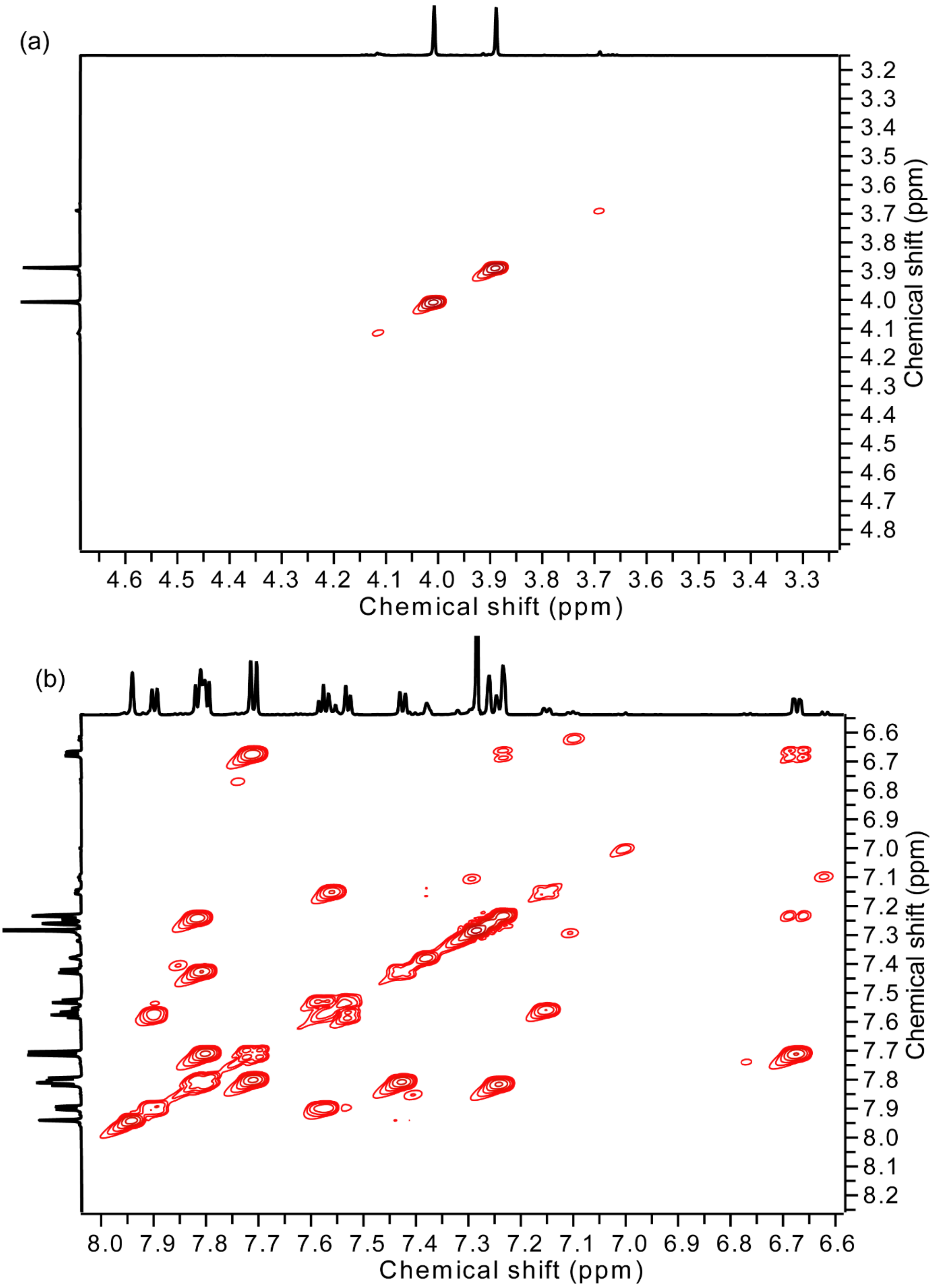

Figure S39. 2D COSY spectra of [4+2] cycloaddition product 2,6'-di-Ome-5-Phe-2-Nap: The 2D COSY spectra (800 MHz instrument, Room temperature, $CDCl_3$ solvent) of [4+2] cycloaddition product 2-methoxy-5-(6-methoxynaphthalen-2-yl)phenanthrene [Named as: 2,6'-di-Ome-5-Phe-2-Nap] in aliphatic region(a) and aromatic region (b). The cross-correlations of peaks are satisfying the required structural criteria for 2,6'-di-Ome-5-Phe-2-Nap product.

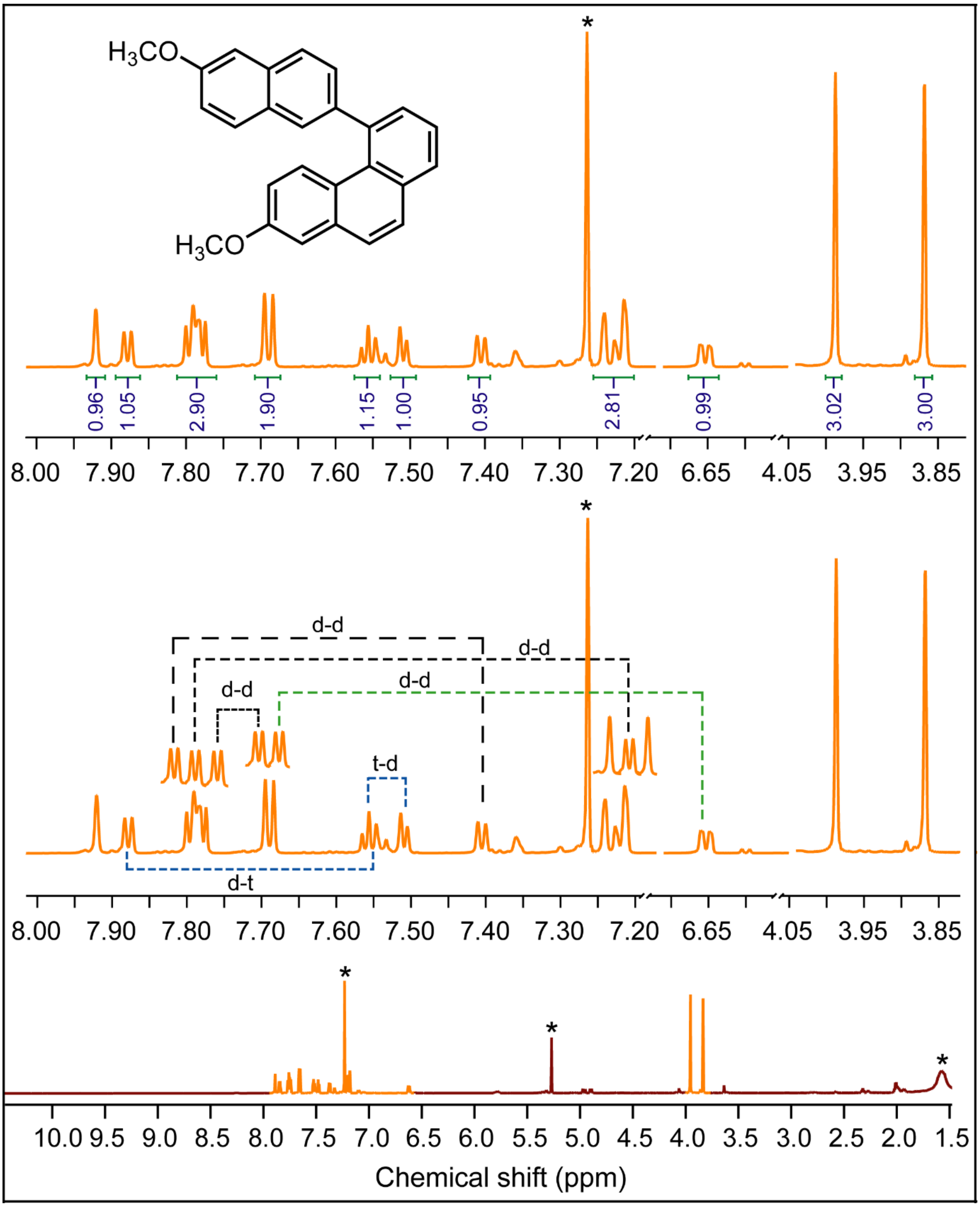

Figure S40. ^{1}H NMR of 2,6'-di-Ome-5-Phe-2-Nap with multiplicities & coupling pattern: ^{1}H NMR spectra of 2,6'-di-Ome-5-Phe-2-Nap molecule with final correlation diagram based on COSY and HSQC data is shown in middle figure with dotted lines. The resolved peaks with their respective multiplicities are shown above the spectra and their coupling patterns are drawn with dotted lines for simple visualisation. After finding peak multiplicity corresponding to each proton present in the structure here is the ^{1}H NMR data with its protons and their individual multiplicity. ^{1}H NMR Data: 800 MHz, $CDCl_3$, Room temperature, Chemical shift(ppm), (7.92, s, 1H), (7.87, d, 1H), (7.81 - 7.76, 3d, 3H), (7.70 - 7.67, 2d, 2H), (7.55, t, 1H), (7.51, d, 1H), (7.40, d, 1H), (7.25 - 7.20, 2s, 1d, 3H), (6.65, d, 1H). '*' marked peaks are from solvent impurities; Around 7.26 ppm and 1.55 ppm are from chloroform and peak around 5.32 ppm is from dichloromethane.

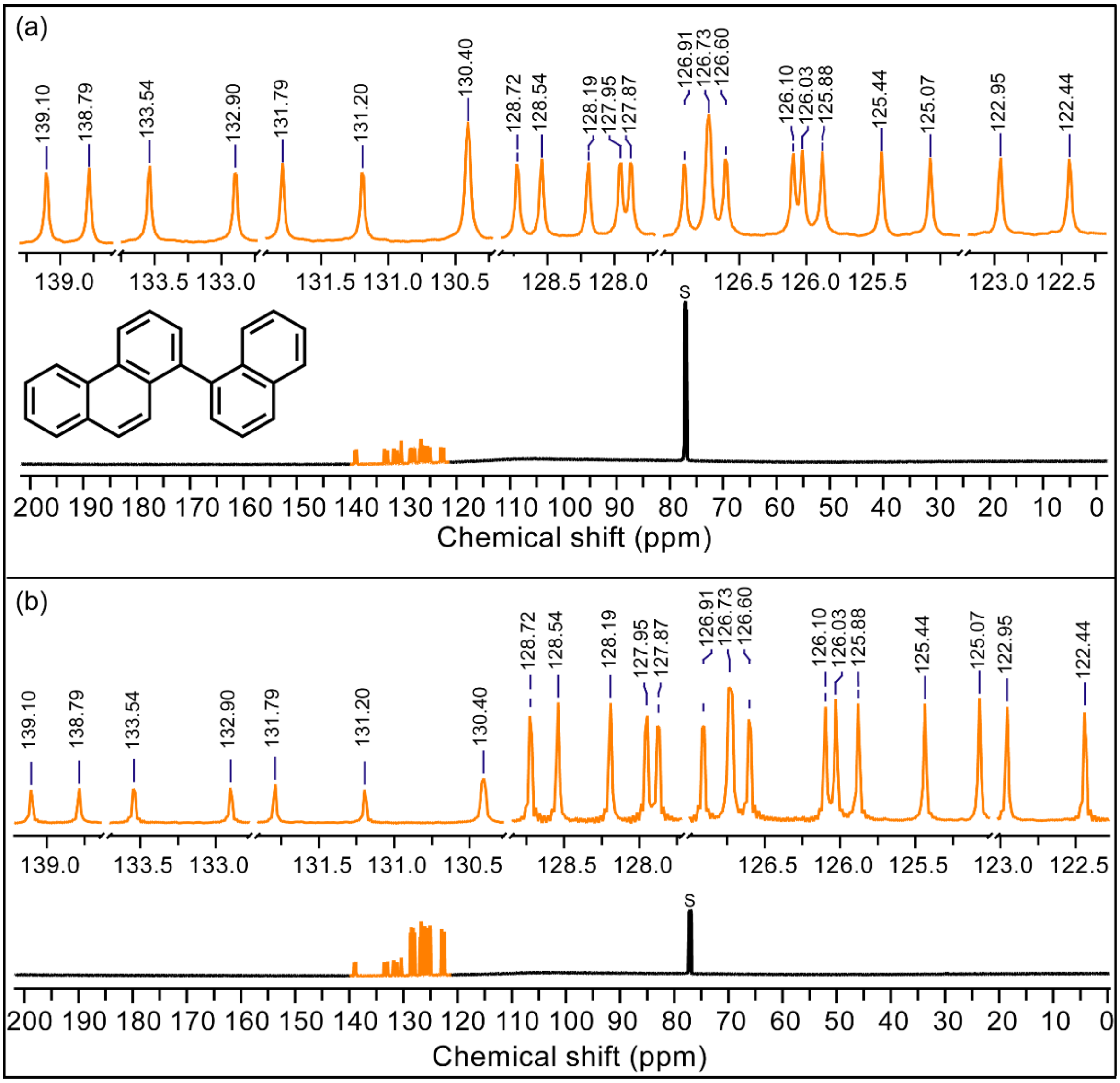

Figure S41. ^{13}C NMR spectra of [4+2] cycloaddition product 1-Phe-1-Nap: ^{13}C NMR spectra of [4+2] cycloaddition product 1-(naphthalen-1-yl)phenanthrene [named as: 1-Phe-1-Nap] recorded in $CDCl_3$ solvent using 800 MHz NMR instrument. From the structure it is clear that there are 16 carbon centres which have 1 proton attached with them and 8 carbon centres with no proton. In ^{13}C spectra (Figure b) there are 16 peaks with high intensity (from carbon having one proton) and 8 peaks with low intensity (from carbon having no proton). The peaks at 126.73 and 130.40 ppm are broader and more intense as compared to the other peaks coming from carbons having one proton and no protons respectively. This hints that these peaks have contributions from 2 carbon nuclei means two peaks are merged in one peak that's why only 15 and 7 peaks are visible. To resolve this and see the clear picture, ^{13}C NMR spectra was recorded in inverse-gated proton decoupling mode, where we do not decouple during the relaxation delay to stop any enhancement of carbon signal due to proton nuclear Overhauser effect (NOE). The ^{13}C spectra was recorded with expectation to see all the signals of same intensity except from two at 126.73 and 130.40 ppm. The Fig (a) clearly shows that 20 peaks have similar intensity and only 2 peaks (at 126.73 and 130.40 ppm) have higher intensity as compared to others. From here we could clearly say that the peaks at 126.73 and 130.40 ppm have contributions from 2 carbon nuclei. The recorded spectra have same number of ^{13}C signals as expected from asymmetric structure confirms the formation of 1-Phe-1-Nap.

The obtained ^{13}C NMR spectra of coupling product (1-Phe-1-Nap) has good match with reported spectra in Liu and co-workers report[22] (*Helv. Chim. Acta* **2021**, *104*, e2100056).

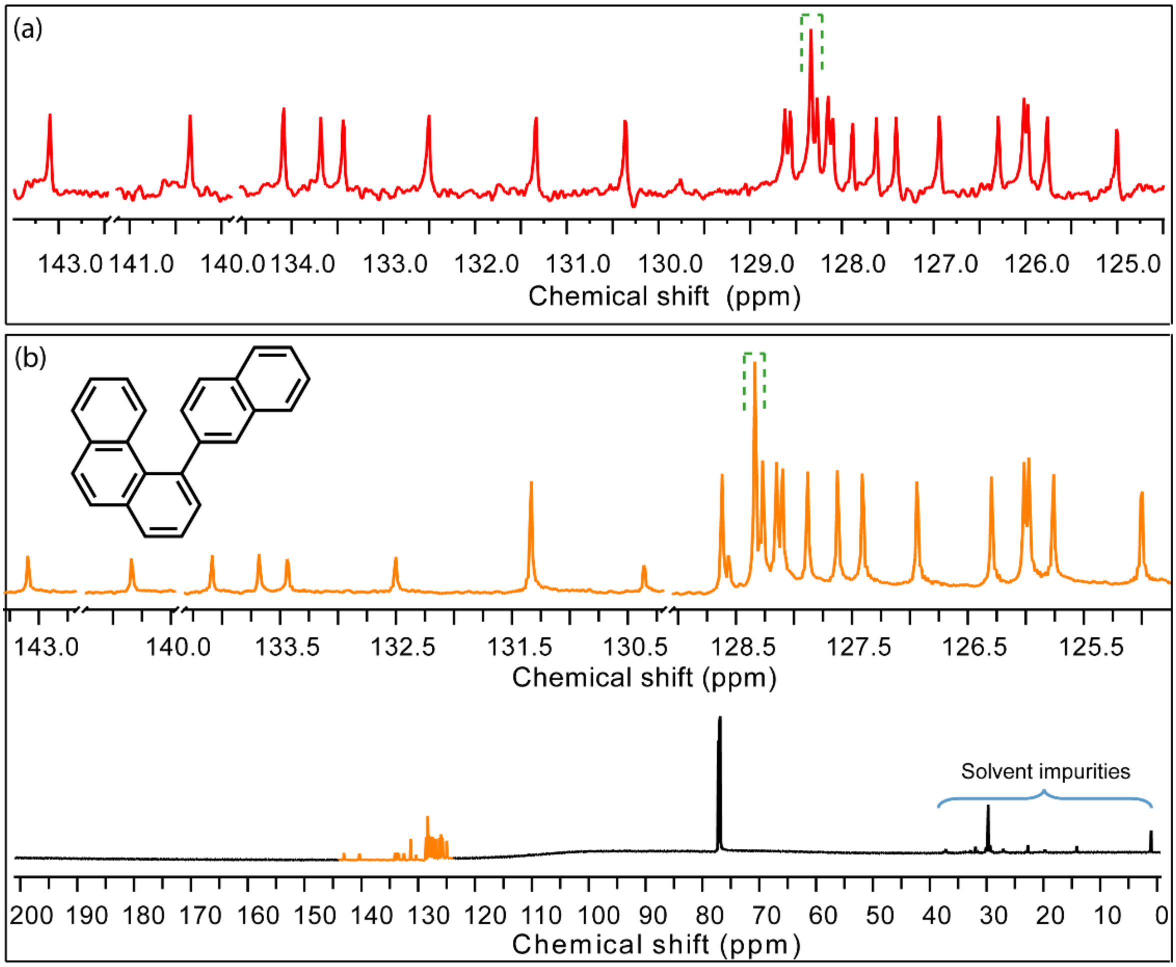

Figure S42. ^{13}C NMR spectra of [4+2] cycloaddition product 4-Phe-2-Nap: ^{13}C NMR spectra of cycloaddition product 4-Phe-2-Nap ($CDCl_3$, 800 MHz instrument). From the structure it is clear that there are 16 carbon centres which have 1 proton attached with them and 8 carbons with no proton. In ^{13}C spectra (Figure b) there are 16 peaks with high intensity (from carbon having one proton) and 8 peaks with low intensity (from carbon having no proton). In this spectrum we could see clear 8 peaks from carbon having no proton but there only 15 peaks are visible from carbon having one proton because the peak at 128.34 ppm (marked with green dashed line) is broader and more intense as compared to the other peaks from carbon having one proton. This hints that peak (128.34 ppm) could have contributions from 2 carbon nuclei. To resolve this and know the exact number of carbon nuclei involved in this peak ^{13}C NMR spectra was recorded in inverse-gated proton decoupling mode, where we do not decouple during the relaxation delay to stop any enhancement of carbon signal due to proton nuclear Overhauser effect (NOE). The ^{13}C spectra was recorded with expectation to see all the signals of same intensity except from one at 128.34 ppm. Fig (a) clearly shows that 22 peaks have similar intensity and only 1 peak at 128.34 ppm have higher intensity as compared to others. From here we could clearly say that the peak at 128.34 ppm have contributions from 2 carbon nuclei. Chemical Shift Values: 143.10 (Cq), 140.34 (Cq), 134.09 (Cq), 133.68 (Cq), 133.44 (Cq), 132.50 (Cq), 131.34 (CH), 130.36 (Cq), 128.62 (CH), 128.56 (Cq), 128.34 (CH), 128.34 (CH), 128.27 (CH), 128.15 (CH), 128.10 (CH), 127.88 (CH), 127.63 (CH), 127.41 (CH), 126.94 (CH), 126.30 (CH), 126.02 (CH), 125.97 (CH), 125.76 (CH), 125.00 (CH). ^{13}C NMR spectra confirms the formation of 4-Phe-2-Nap. Solvent impurities marked peaks are from n-hexane and ethyl acetate which were used in isolation and purification.

The obtained ^{13}C NMR spectra of (4-Phe-2-Nap) has good match with reported spectra in following reports:

Liu and co-workers[22] (*Helv. Chim. Acta* **2021**, *104*, e2100056)

Fleming and co-workers[23] (*Angew. Chem. Int. Ed.* **2016**, *55*, 14770 –14773).

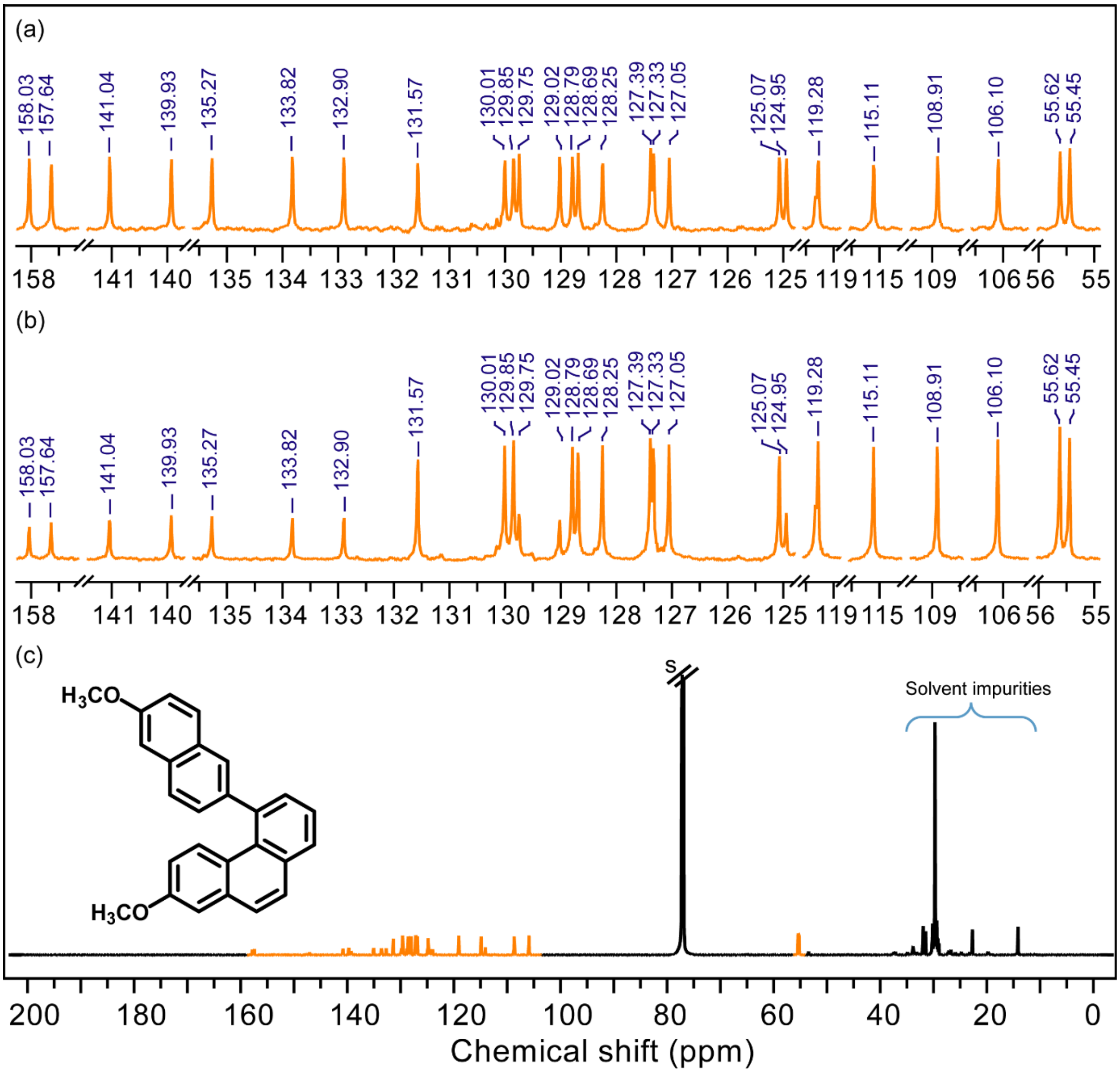

Figure S43. ^{13}C NMR spectra of [4+2] cycloaddition product 2,6'-di-Ome-5-Phe-2-Nap: ^{13}C NMR spectra of [4+2] cycloaddition product 2-methoxy-5-(6-methoxynaphthalen-2-yl)phenanthrene [Named as: 2,6'-di-Ome-5-Phe-2-Nap] recorded in $CDCl_3$ solvent using 800 MHz NMR instrument. Complete NMR spectra of 2,6'-di-Ome-5-Phe-2-Nap (c) and its zoomed-in spectra is shown in figure (b). The ^{13}C NMR spectra of 2,6'-di-Ome-5-Phe-2-Nap in inverse-gated proton decoupling mode is shown in figure (c). From the structure it is clear that there are 16 carbon centres which have proton attached with them and 10 carbon centres with no proton. In the recorded ^{13}C spectra there are 16 peaks with high intensity (from carbon having proton) and 10 peaks with low intensity (from carbon having no proton). The peaks in aliphatic region (55 ppm) are from the two methoxy group present in the molecule and the rest peaks present in aromatic region are from the carbon atoms of naphthalene and phenanthrene rings. The inverse-gated proton decoupled mode spectra have 26 peaks of equal intensities which aligns with spectra (b). The presence of required number of carbon signal with their respective peak positions and intensities confirms the formation of 2,6'-di-Ome-5-Phe-2-Nap.

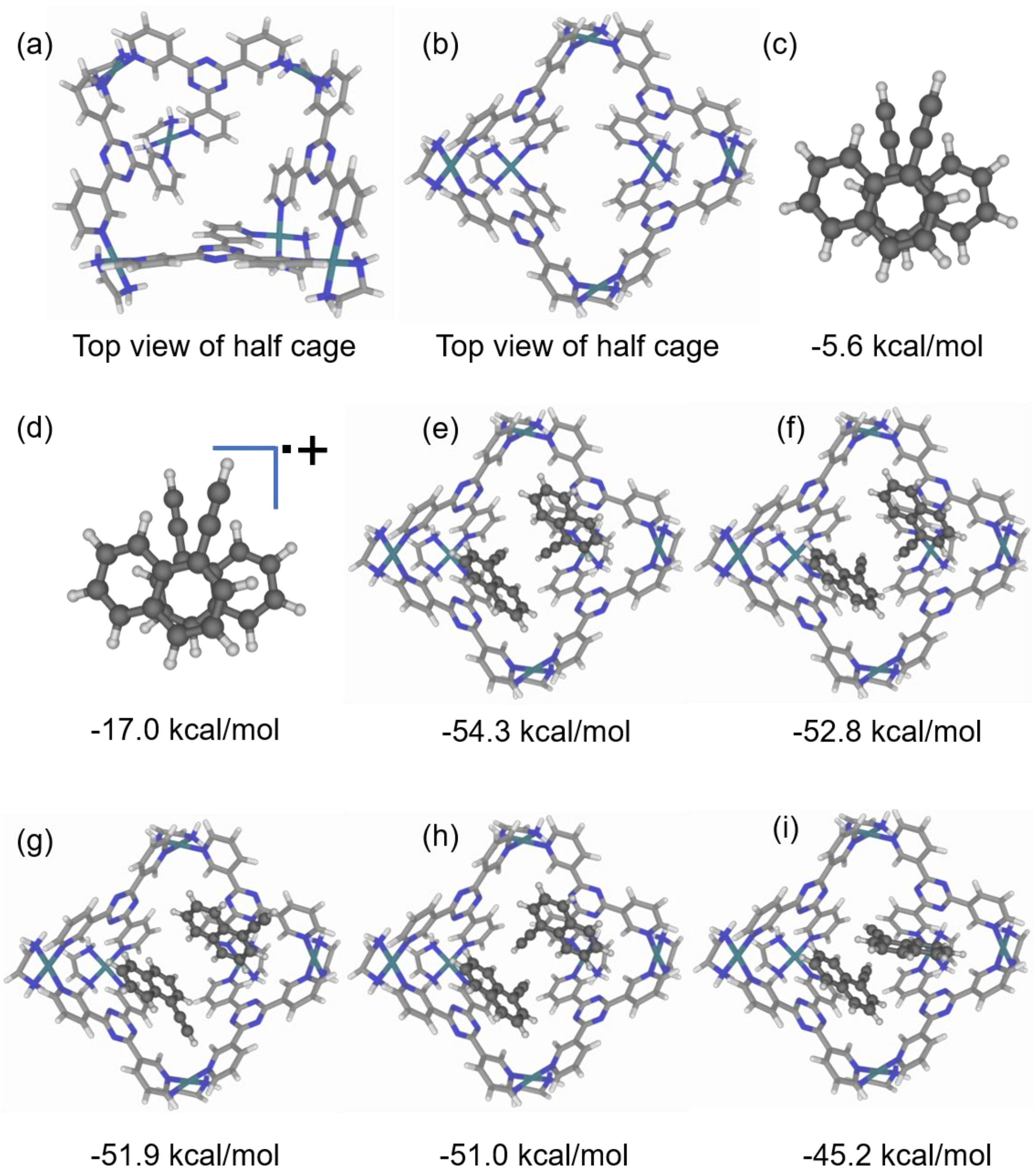

Figure S44. Optimized conformations of 1-ENap molecules within half cage: (a) Top view of half cage used in Figure 3 and (b) Top view of half cage for Figures (e-i). Optimized conformation of (c) 1-ENap dimer in gas phase and (e-i) 1-ENap dimer in various orientations within rigid half cage. All optimizations are done using PBE-D3 functional and def2-SVP basis set. (d) Interaction energy of radical cation 1-ENap dimer at optimized neutral geometry (c). All interaction energies are computed using PBE0-D4 and def2-SVP basis set. The final optimized conformations (e-h) have an average interaction energy of -52.5 kcal/mol and their energies are varying within 2 kcal/mol range.

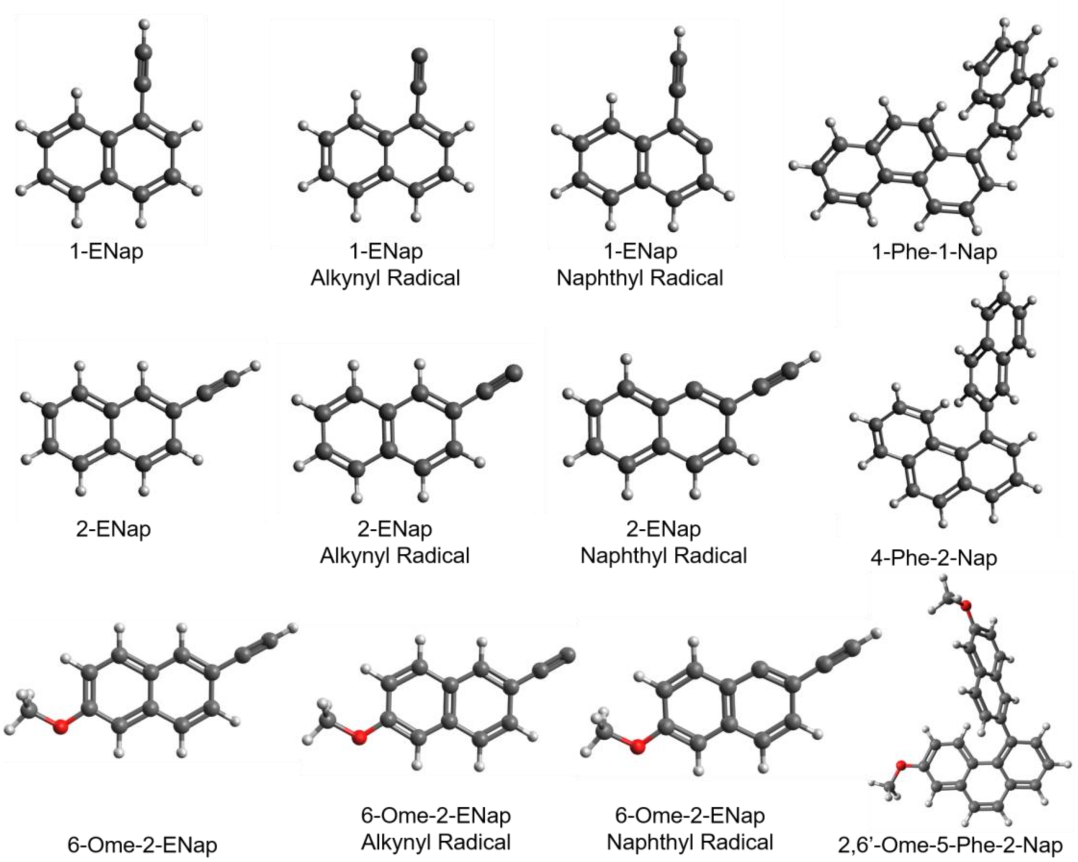

Figure S45. Optimized structure of reactants, proposed intermediates and products: Optimized geometries of 1-ENap and 2-ENap, their neutral alkynyl and naphthyl radical and their coupling product 1-Phe-1-Nap and 4-Phe-2-Nap.

Reaction	PBE0-D4 Energy (eV)
1-ENap → 1-Phe-1-Nap	- 4.85
2-ENap → 4-Phe-2-Nap	- 4.96
6-Ome-2-ENap → 2,6'-di-Ome-5-Phe-2-Nap	- 4.82

Table S1: Reaction enthalpies of [4+2] cycloaddition reaction using PBE0+D4/def2-TZVPP level of theory. All values are in eV.

Molecule	Stability (eV)
1-ENap	- 0.54
2-ENap	- 0.60
6-Ome-2-ENap	- 0.51

Table S2: Stability of Naphthyl radical compared to alkynyl radical ($E_{Naphthyl} - E_{Alkynyl}$) computed using PBE0+D4/def2-TZVPP level of theory. All values are in eV.

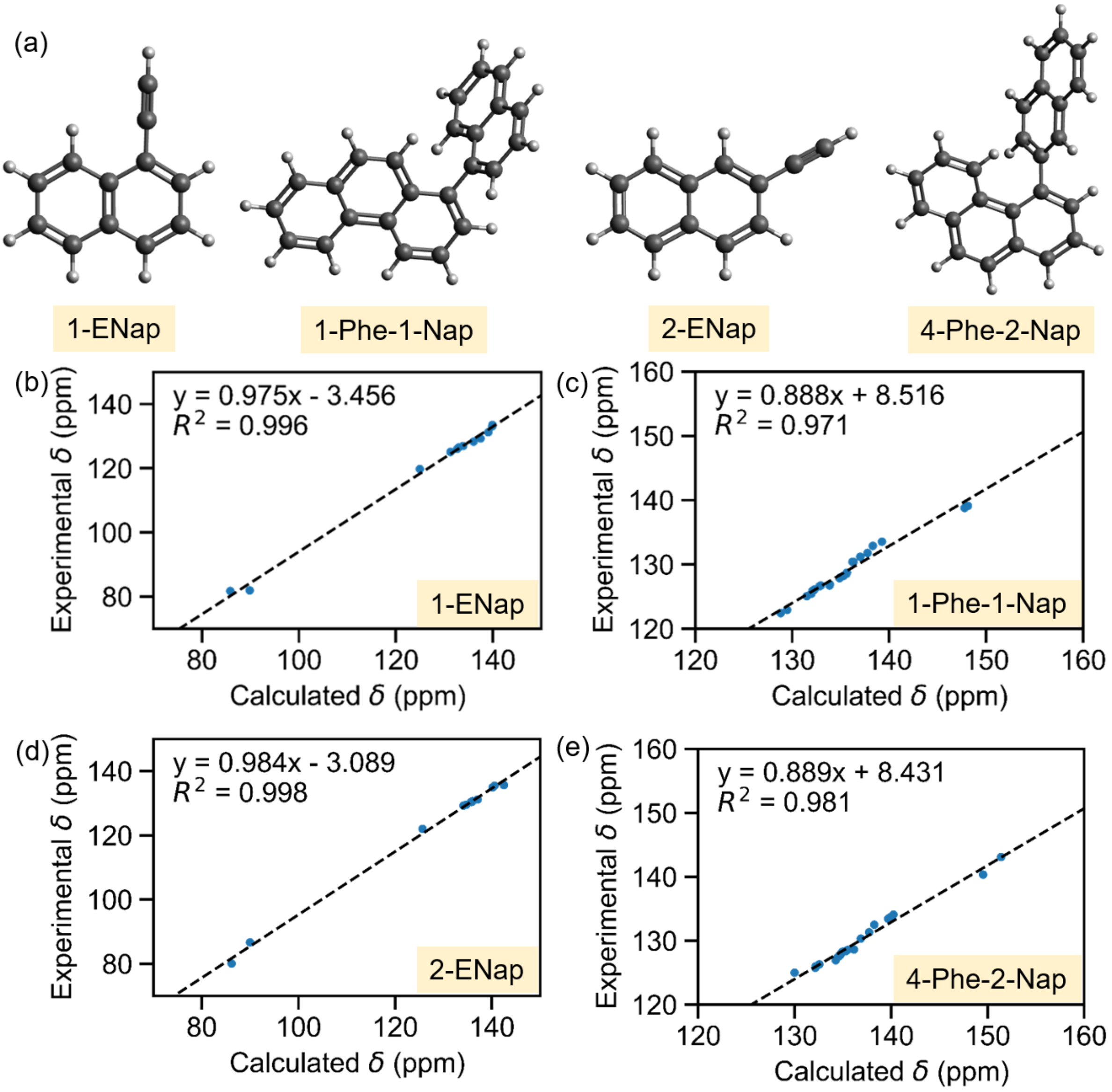

Figure S46. Theoretical and experimental ^{13}C NMR spectra of reactants and products: (a) Optimized structure of 1-ENap, 2-ENap, 1-Phe-1-Nap and 4-Phe-2-Nap, (b-e) their respective correlation plots between experimental and theoretical ^{13}C NMR spectra of 1-ENap, 2-ENap, 1-Phe-1-Nap and 4-Phe-2-Nap. Theoretical spectra are computed using CENSO[11], utilizing PBE0 functional[15] (with D4 dispersion correction) and def2-TZVPP basis set[17] and $CHCL_3$ as solvent. Conformational averaged chemical shift values are reported for computed spectra. All ab initio electronic structure calculations were conducted using version 5.0.4 of the ORCA package[8].

Since computed NMR spectra usually have a shift w.r.t experimental values due to basis-set deficiencies and electron-correlation effects[24; 25], we use the correlation of the theoretical peak patterns with the experimental results as a measure of the exactness of the structure.[26-28] For the 1-ENap, we obtain an R^2 value of 0.996 indicating an excellent correlation with the experimental ^{13}C NMR peak pattern thereby confirming our benchmarks and the choice of DFT functionals and basis-sets. For the coupling product (1-Phe-1-Nap), the correlation pattern for coupling product has R^2 value of 0.971 which is also a very good correlation confirming the final product formation. A similar result is observed for the ^{13}C-NMR spectra of 2-ENap and 4-Phe-2-Nap.

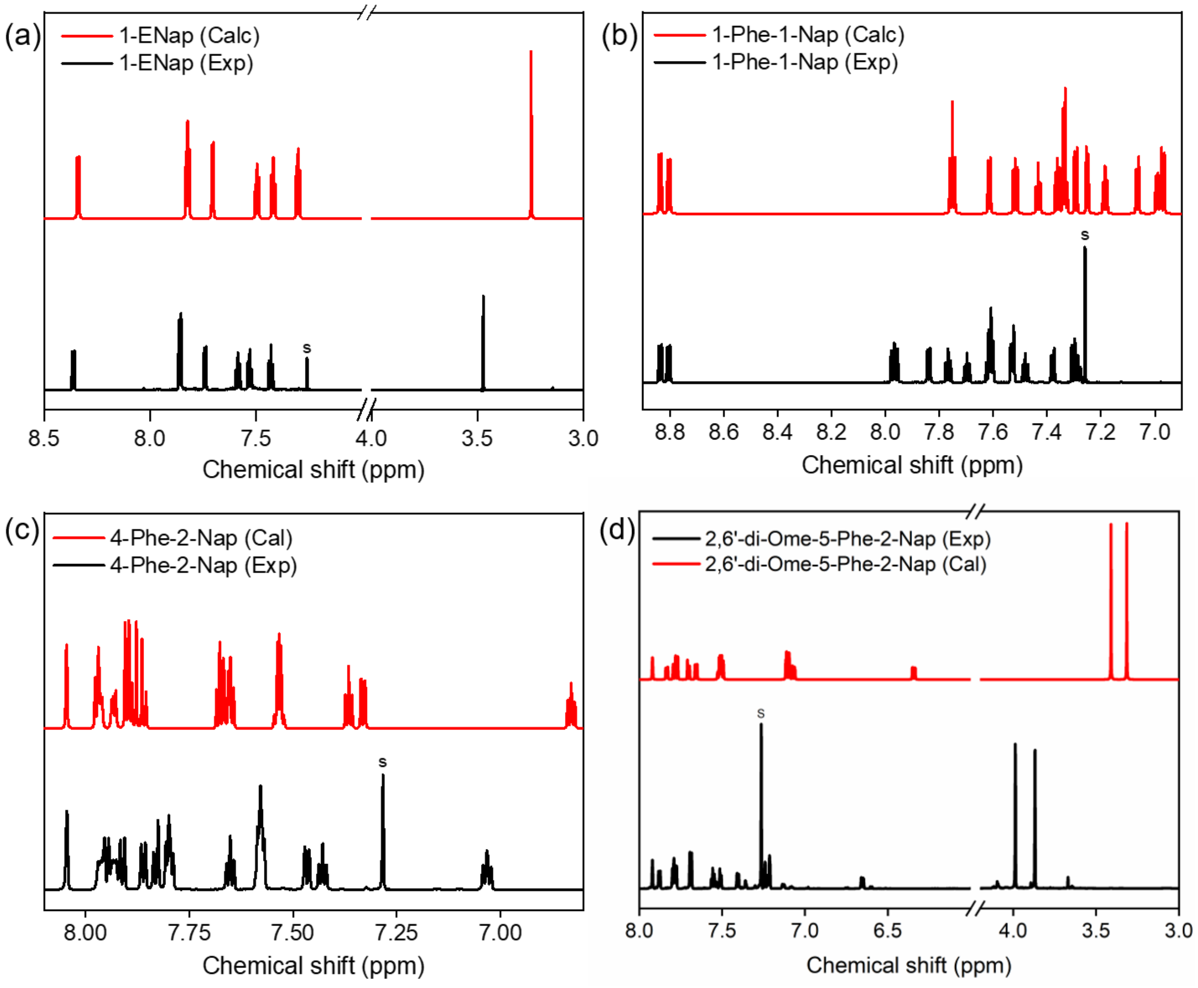

Figure S47. Theoretical and experimental ^{1}H NMR spectra of reactants and products: Comparison of experimental ^{1}H NMR spectra with calculated ^{1}H NMR spectra of 1-ENap (a), 1-Phe-1-Nap (b), 4-Phe-2-Nap (c), and 2,6'-di-Ome-5-Phe-2-Nap (d). The computational details are given in section 1.3. All the ^{1}H NMR spectra are recorded in CDCl3 and the solvent peak is marked with ‘s’. The calculated spectra are shifted with respect to most downfield peak of experimental NMR spectra.

The computed ^{1}H-NMR spectra of the coupling product 1-Phe-1-Nap after benchmarking for 1-ENap using CENSO[11], is in good agreement with experimental spectra (Figure a-b). We identified same coupling patterns as in experimental data, 2 sets of correlations involving *d-t-d* like coupling pattern, 2 sets of correlations pertaining to *d-t-t-d* like coupling pattern and 2 doublets coupled with each other arising from coupling between hydrogens of central ring of phenanthrene moiety. Similar results are obtained for ^{1}H-NMR spectra of coupling product 4-Phe-2-Nap (Figure c) and 2,6'-di-Ome-5-Phe-2-Nap (Figure d). The calculated and experimental NMR peak positions are agreeing within 0.1-0.25 ppm values. The experimental and theoretical ^{1}H NMR spectra have a good match which supports the formation of coupling products in the reaction.

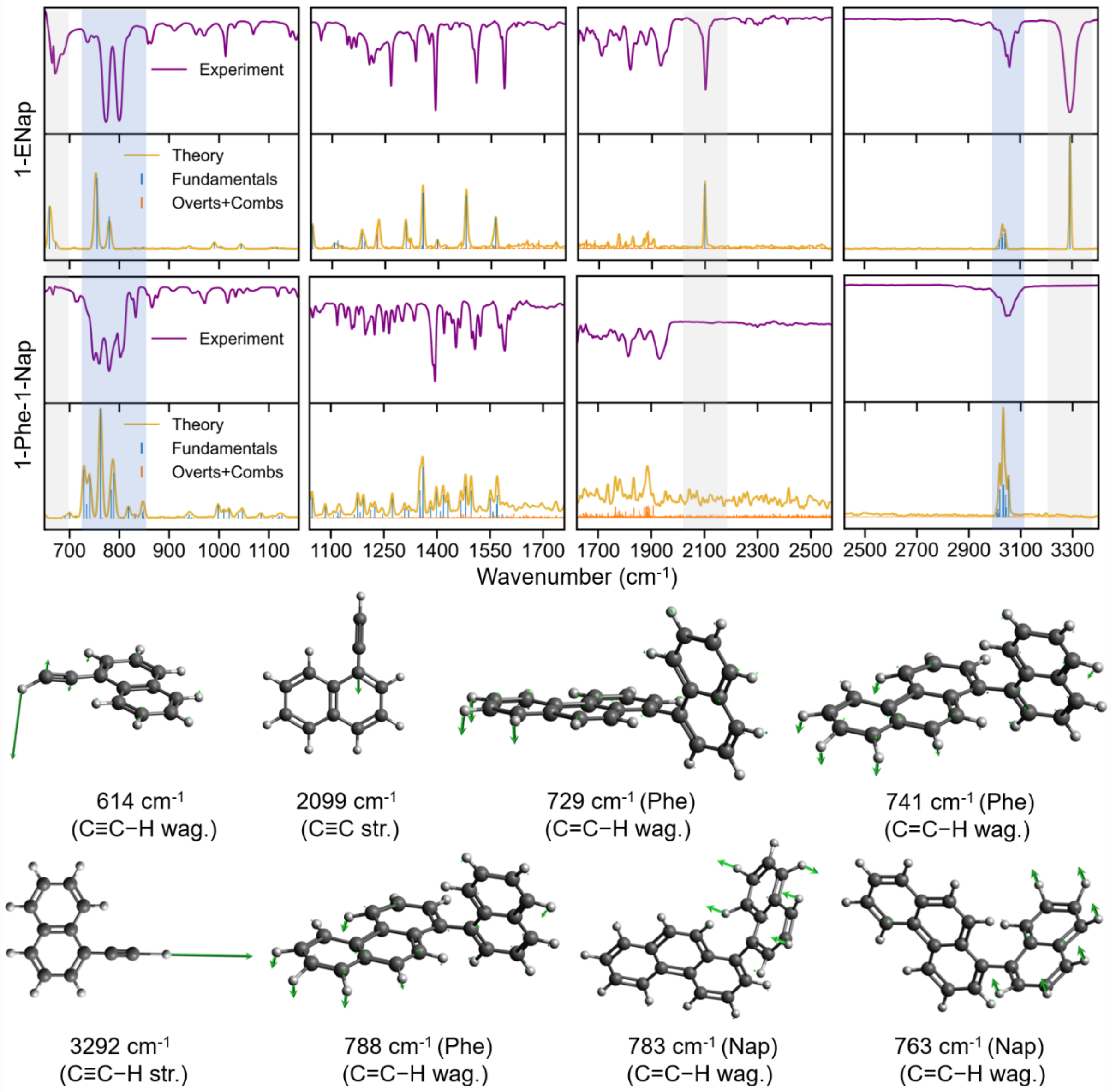

Figure S48. Theoretical and experimental FT-IR spectra of 1-ENap and 1-Phe-1-Nap: IR spectra of 1-ENap and 1-Phe-1-Nap. Upper and lower panel in each figure indicate experimental and calculated IR spectra respectively. Theoretical spectra are computed using PBE0-D4 and def2-TZVPP basis set. Scaling of 0.94516 and gaussian broadening of 5 cm-1 is used for all computed spectra. Grey transparent box contains peak corresponds to alkyne bond in 1-ENap while blue transparent box contains peak from naphthalene and phenanthrene moieties of coupling product 1-Phe-1-Nap. In computed spectra blue and red sticks corresponds to fundamentals and overtones/combined frequencies respectively.

The experimental IR spectra of 1-Phe-1-Nap is highly rich in the frequency ranges between 3000 to 3100 cm^{-1} and shows disappearance of the alkyne C-H stretch at 3292 cm^{-1} while having the C-H stretches from the naphthalene and phenanthrene moiety ~3050 cm^{-1}. Additionally, small features in the range of 1600 to 2000 cm^{-1} is always observed for naphthalene core arising from overtones and combination bands. However, the region from 700 cm^{-1} to 1600 cm^{-1} is very congested, which required theoretical modeling for clear assignment of the vibrational features. We computed the theoretical IR spectra at the PBE0-D4/def2-TZVPP level, with overtones and combination frequencies computed using the semi-empirical GFN2-XTB method[29]. We found a good agreement between the experimental and theoretical spectra in all regions. Using the theoretical spectra, we identified the peaks corresponding to the alkyne group in the 1-ENap, appearing at 3292 cm^{-1} (CH stretching of *sp* carbon),

2099 cm^{-1} (*sp* C≡C stretching), 660 cm^{-1} (CH bending of *sp* carbon), and 614 cm^{-1}(CH wagging of *sp* carbon) as shown in Figure 4 (bottom). The absence of these peaks in the coupling product (1-Phe-1-Nap) IR spectra indicates reaction of the alkyne group**.** In case of 1-Phe-1-Nap, in high frequency range, vibrational features specific to naphthalene or phenanthrene are hard to assign due to overlap. However, in the low frequency region of the computed spectra, we observe peaks at 729, 741 and 788 cm^{-1} corresponds to CH wagging of sp^2 carbon of phenanthrene ring and at 763 and 783 cm^{-1} corresponding to CH wagging of sp^2 carbon of naphthalene ring (appearing at 755 and 778 cm-1 in 1-ENap). All these assigned modes confirm the formation of the 1-Phe-1-Nap product.

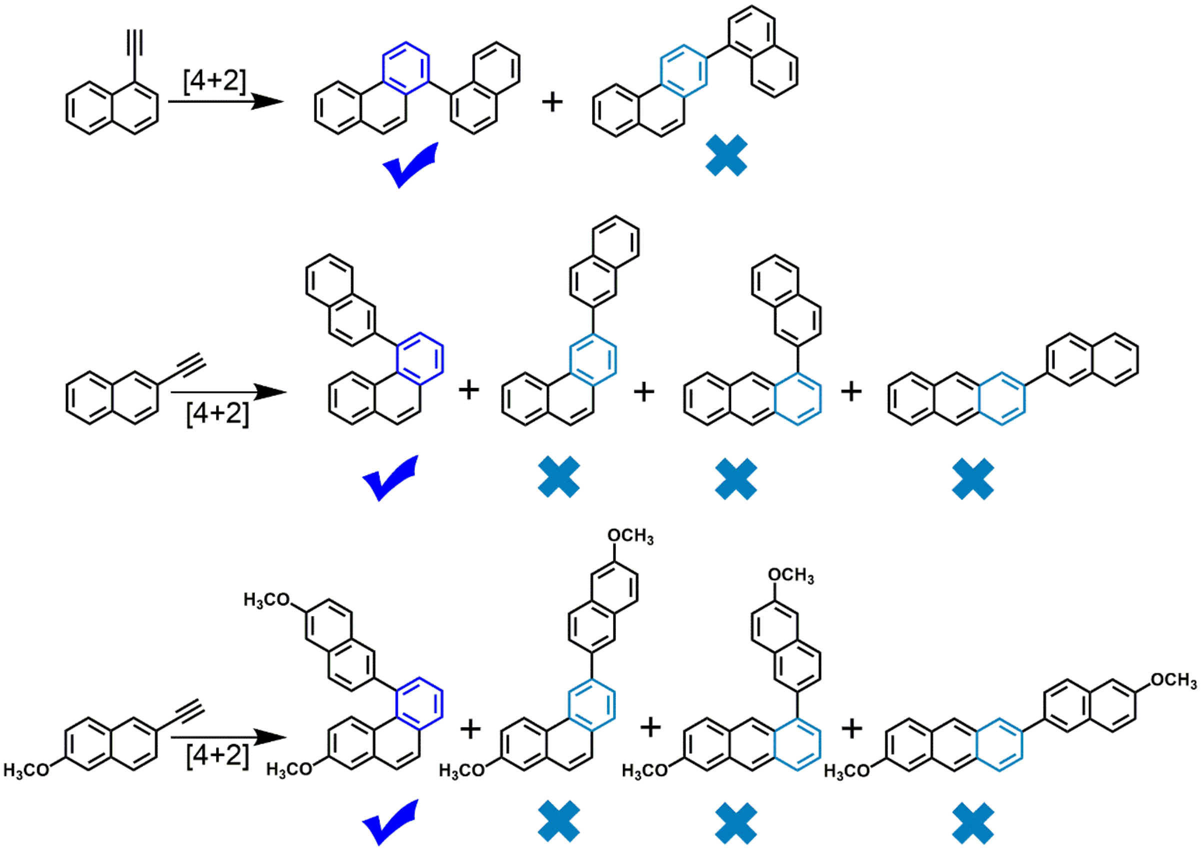

Figure S49. The possible coupling products in reaction: Possible [4+2] cycloaddition products in case of 1-ENap, 2-ENap and 6-Ome-2-ENap. Relative percentages may vary with reaction conditions. In our scheme, reaction inside the confinement there is formation of selectively one product which could be due to the pre-organized orientations of reactant molecules induced by confinement.

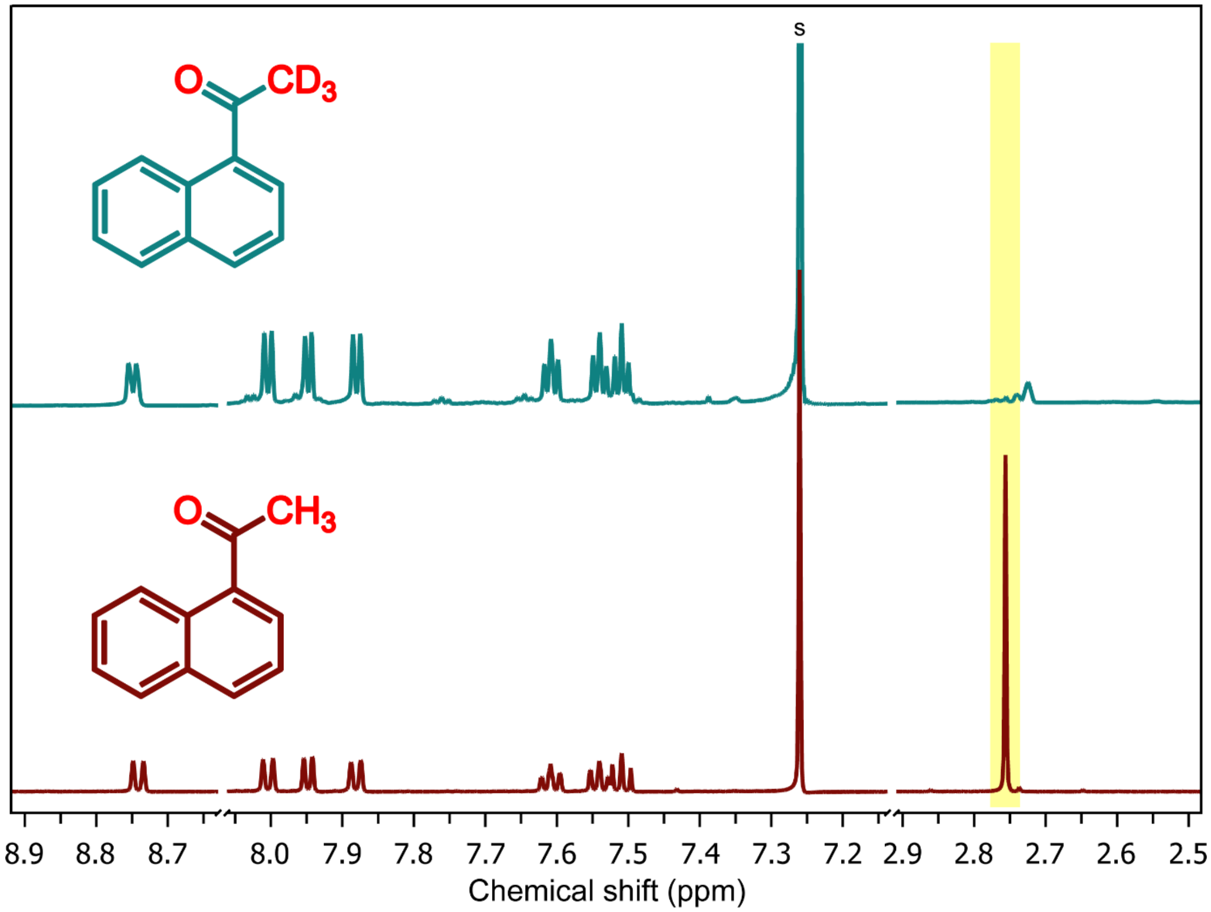

Figure S50. ^1H NMR spectra of alkyne hydration products 1-acetonaphthone-H_3/D_3: ^1H NMR spectra of alkyne hydration (oxidation) product (1-acetonaphthone-H_3/D_3) obtained after photoreaction in H_2O solvent (bottom spectra, 600 MHz instrument, $CDCl_3$) and D_2O solvents (top spectra, 800 MHz instrument, $CDCl_3$). From the ^1H NMR spectra it is clear that all the aromatic proton peaks are present in the photoproduct obtained after reaction in D_2O solvent except alkyl proton peak (aliphatic region) which is highlighted with yellow coloured shaded region (δ2.76 ppm). The absence of alkyl proton peak in upper spectra (region highlighted in yellow colour, around δ2.76 ppm) confirms the presence of deuterium atom at the alkyl position and this ultimately supports the formation of oxidation product with deuterium atom i.e., $C_{12}H_7D_3O$ (1-acetonaphthone-D_3).

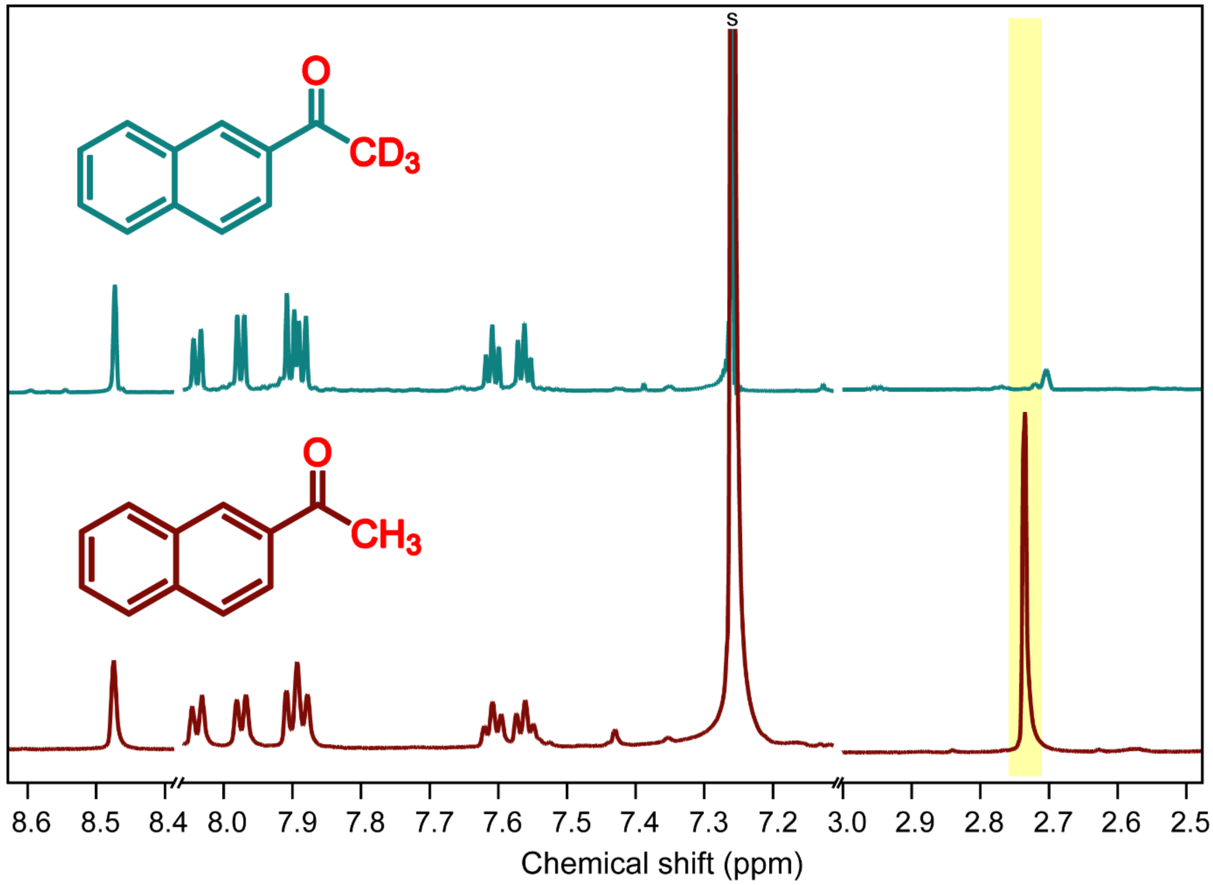

Figure S51. ^{1}H NMR spectra of alkyne hydration products 2-acetonaphthone-H_3/D_3: ^{1}H NMR spectra of oxidation product (2-acetonaphthone-H_3/D_3) obtained after photoreaction in H_2O solvent (bottom spectra, 600 MHz instrument, $CDCl_3$) and D_2O solvents (top spectra, 800 MHz instrument, $CDCl_3$). From the ^{1}H NMR spectra it is clear that all the aromatic proton peaks are present in the photoproduct obtained after reaction in D_2O solvent except alkyl proton peak (aliphatic region) which is highlighted with yellow coloured shaded region (δ2.74 ppm). The absence of alkyl proton peak in upper spectra (region highlighted in yellow colour) confirms the presence of deuterium atom at the alkyl position and this ultimately supports the formation of oxidation product with deuterium atom i.e., $C_{12}H_7D_3O$ (2-acetonaphthone-D_3).

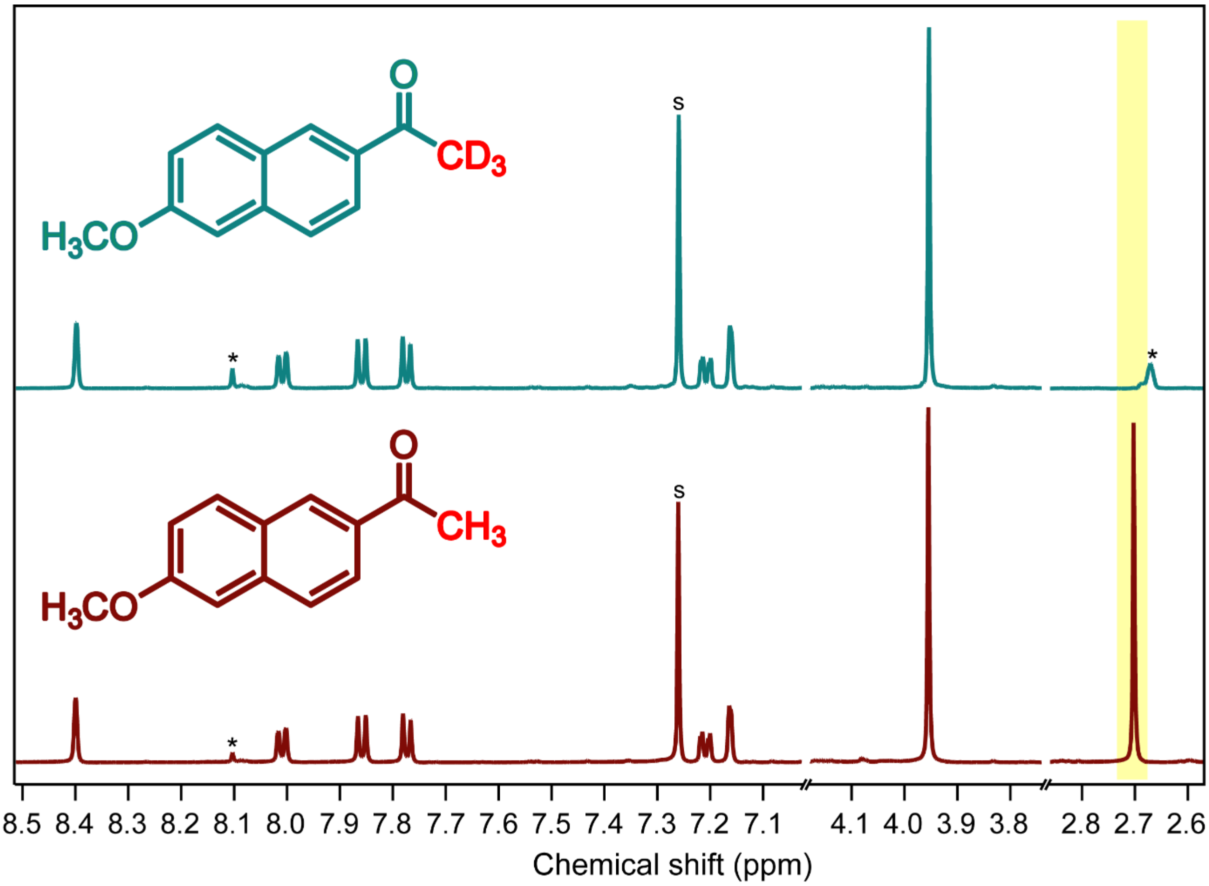

Figure S52. ^{1}H NMR spectra of alkyne hydration products 2-acetyl-6-methoxynaphthalene -H_3/D_3: ^{1}H NMR spectra of oxidation product (2-acetyl-6-methoxynaphthalene-H_3/D_3) obtained after photoreaction in H_2O solvent (bottom spectra, 600 MHz instrument, $CDCl_3$) and D_2O solvents (top spectra, 600 MHz instrument, $CDCl_3$). From the ^{1}H NMR spectra it is clear that all the aromatic proton peaks are present along with proton peak from methoxy group (δ3.95 ppm) in the photoproduct obtained after reaction in D_2O solvent except alkyl proton peak at δ2.70 ppm which is highlighted with yellow coloured shaded region. The absence of alkyl proton peak in upper spectra (region highlighted in yellow colour) confirms the presence of deuterium atom at the alkyl position and this ultimately supports the formation of oxidation product with deuterium atom i.e., $C_{13}H_9D_3O_2$ (2-acetyl-6-methoxynaphthalene-D_3).

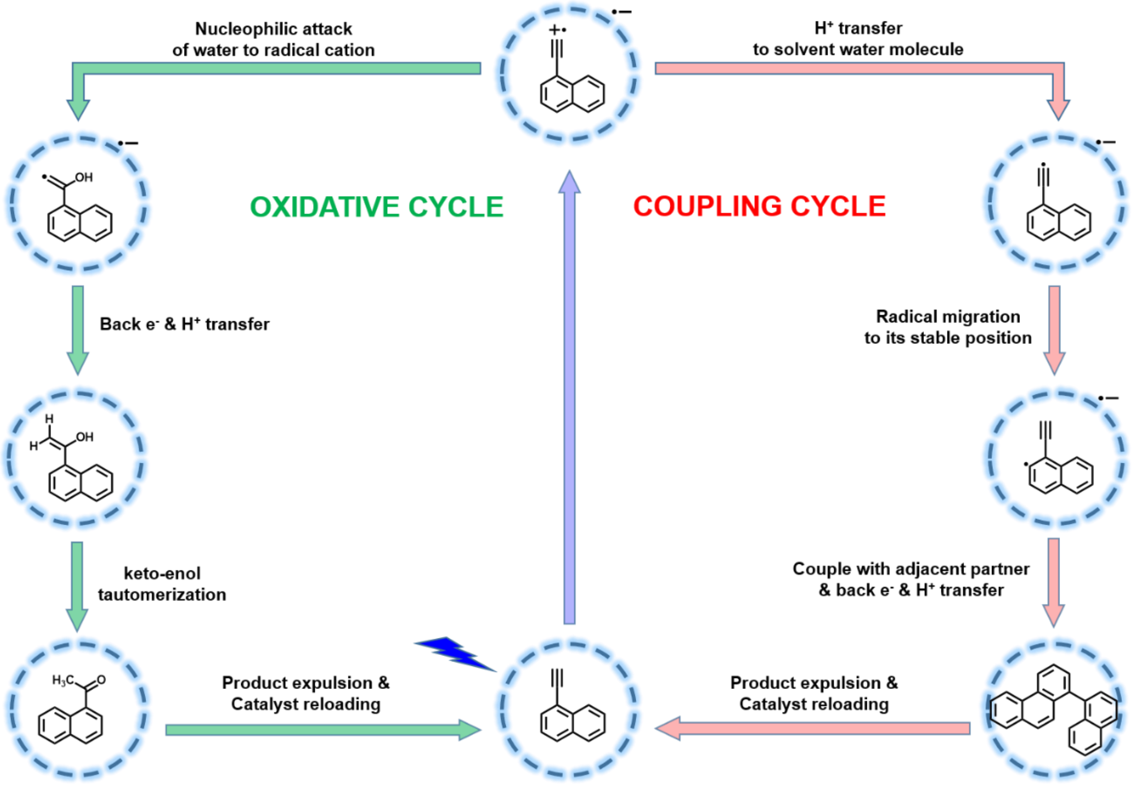

Figure S53. Proposed reaction mechanism of alkyne hydration reaction inside full cage: The possible reaction mechanism for alkyne hydration reaction (green, oxidative cycle) and coupling reaction (red, coupling cycle) inside full-cage.

The light excitation on H-G complex in CT band region induces structural and electronic changes in reactant molecule in excited state which drives photoinduced electron transfer process from aromatic guest molecule (1-ENap) to full-cage leads to the generation of radical-cation on the guest and radical anion on the full-cage. From this point there are two possibilities which are mentioned below:

Oxidative cycle: The radical cation inside full-cage is exposed to pre-organized water molecules due to free pores on alternate faces of the octahedra hence nuclephilic attack of water molecule on radical cation forms a nuetral radical species with hydroxyl functionality on vinylic double bond. In next step the back electron and proton transfer from cavity and solvent molecules respectively leads the formation of enol form of product which subsequently tautomerises to keto form. Next the reactant molecules which are in excess in free solution displace the product molecules and continue the catalytic cyle.

Coupling cycle: In some cases where the reactive partners are in proper orientations for coupling reaction to take place, the neutral radical formed after the deprotonation steps re-arranges to its stable form (naphthyl radical) which interacts with neighbouring neutral partner and leads the formation of coupling product. Next the reactant molecules which are in excess in free solution displace the product molecules and continue the catalytic cyle.

To track the proposed intermediates of catalytic cycle femtosecond transient absorption (TA) measurement is done on 1-ENap ⊂ Full Cage H-G CT complex by exciting the sample in CT band region using 400 nm pump pulse and the evolution of excited state is monitored using broadband while-light probe pulse. The Transient absorption data is summarised in next figure.

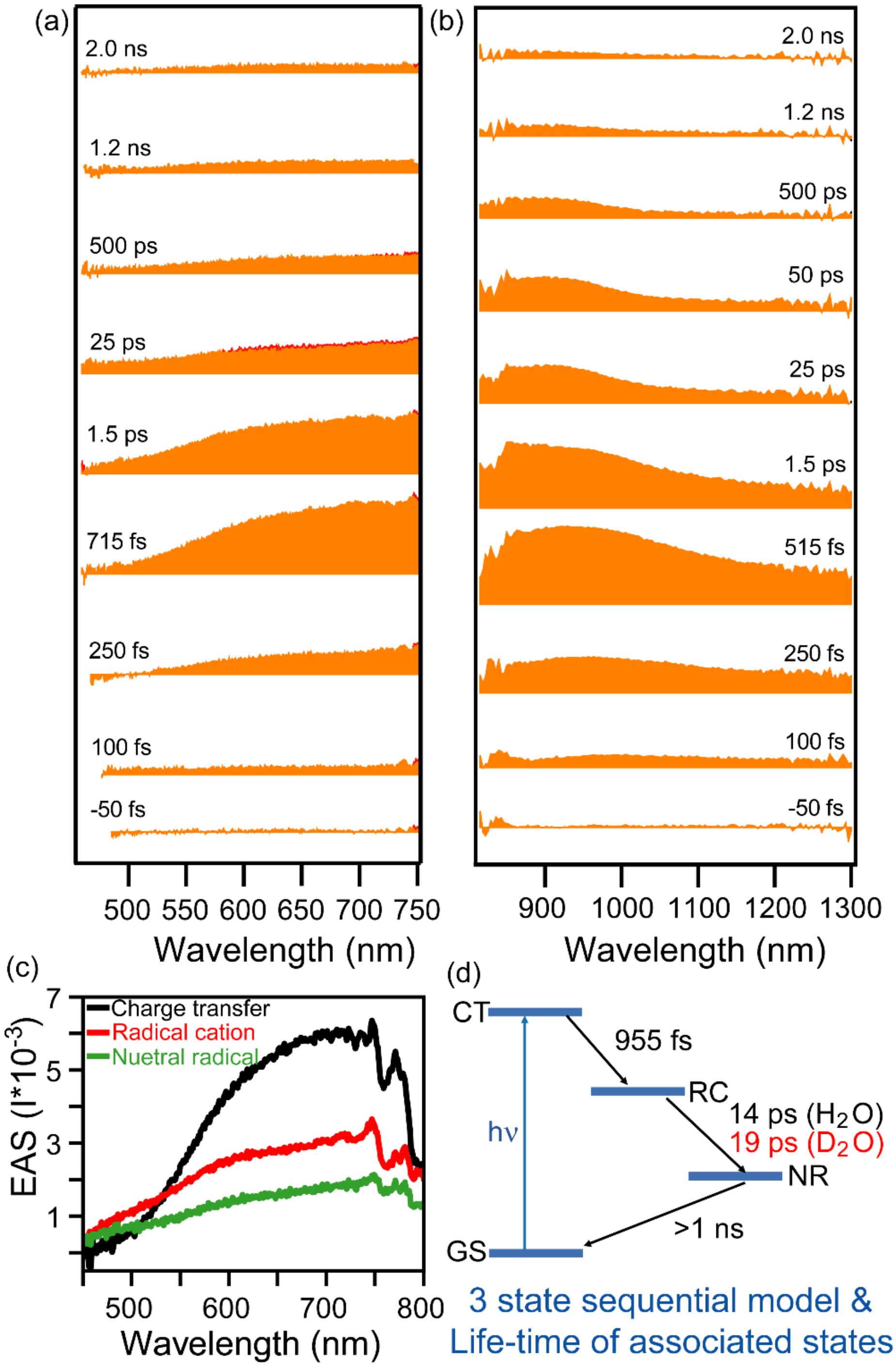

Figure S54. Transient absorption spectra of 1-ENap ⊂ 1 H-G complex: Transient absorption (TA) data of 1-ENap ⊂ Full cage H-G CT complex: (a) TA spectra in the visible probe window, (b) NIR probe window. TA spectra show the increase in the population of excited state absorption (ESA) spectra first, then it started to decrease with non-decaying population up to 2 ns. (c) The excited state absorption (ESA) spectra of species associated / generated after photoexcitation on H-G CT complex. The photoexcitation led to the generation of radical cation (red spectra) at guest molecule and radical anion on full cage simultaneously.[6; 30] The feature in near IR region arises from the host-guest CT interaction and the radical anion state of full cage (after solvation dynamics timescale).[6; 31] The radical cation interacts with water molecules in the vicinity and generate neutral radical species (green spectra) which have life time of more than 1 ns. (d) The possible model for this system: 3-state sequential model, and lifetime of associated states. The Charge-transfer state (CT: 955 fs), Radical cation (RC: 14 ps in H_2O and 19 ps in D_2O), Neutral radical (NR: >1 ns). The different decay rate of radical cation state in H_2O *vs* D_2O solvent suggest that the solvent molecules are playing role in this process.

5mM, 2mL Half cage solution in H_2O + 8 equivalent 1-Ethynylnaphthalene —(400nm LED, $t_{h\nu}$ = 14h)→ Photoproducts

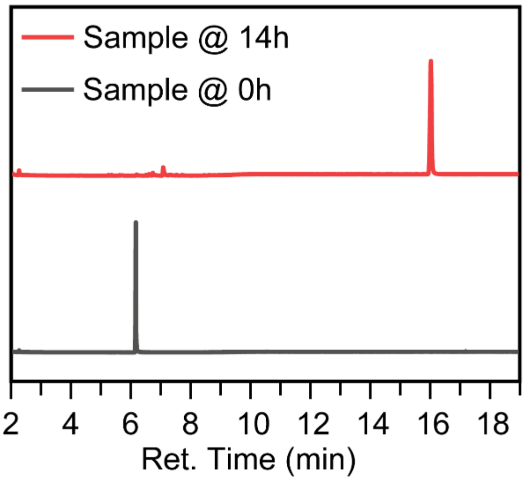

Ret. Time (min)	Mass (Products)	Relative % (Products)
6.19	152	0.5
7.08	170	6.5
16.02	304	93.0

Figure S55. Photocatalytic cycles of 1-ENap ⊂ 2 H-G complex system: Photoreaction scheme (top), GCMS chromatogram before (sample @ 0h) and after (sample @ 14h) photoreaction, the relative percentages of the photoproducts are shown in table.

Photoreaction procedure: 8 equivalents of 1-ethynylnaphthalene with respect to amount of half cage was added in the solution of half cage (5mM, 2mL) and stirred at room temperature for 1.5 h. The H-G complex solution with excess guest molecules was then irradiated in its CT band region using 400 nm LED for 14 h. The photoproducts extraction done in $CHCl_3$ and relative percentages were analysed with GCMS. The GC-MS data shows that this scheme is performing very well with high selectivity of 93 % towards [2+2] cycloaddition product up to four catalytic cycle.

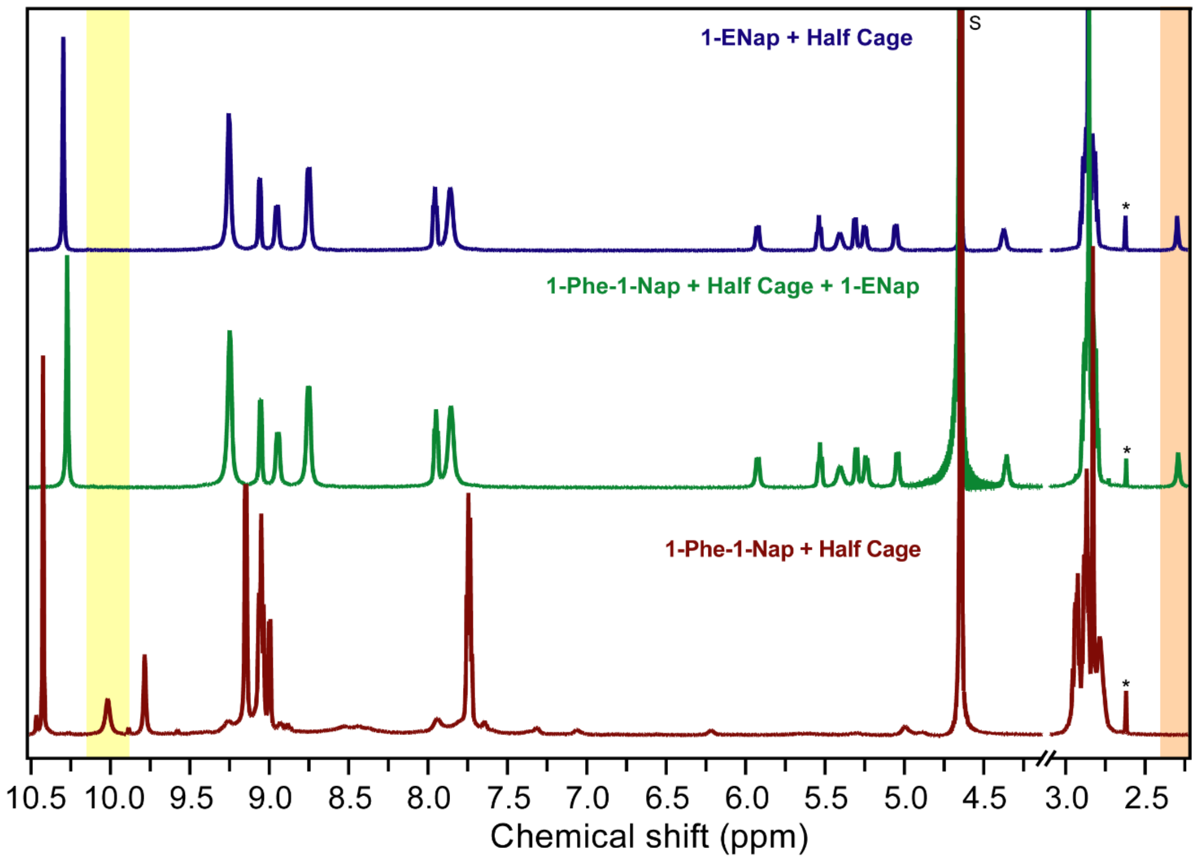

Figure S56a. Control experiment - Product displacement by reactant molecules: Displacement of cycloaddition product from half cage by the reactant molecule. ^{1}H NMR spectra of H-G Complex 1-Phe-1-Nap incarcerated in Half Cage (1-Phe-1-Nap + Half Cage, crimson colour) shows that the coupling product molecule interacts with half cage. There are several small-small peaks in between 10.25 – 5.0 ppm which indicates that there is heterogeneity in system and some part of the 1-Phe-1-Nap is inside while the rest part might be hanging outside the half cage therefore we do not see upfield shift in all the peaks of 1-Phe-1-Nap molecule. To test the displacement of coupling product (1-Phe-1-Nap) molecule by reactant molecules (1-ENap), we added the 1-ENap in the H-G complex solution of 1-Phe-1-Nap incarcerated in half cage and stirred at room temperature for 45 minutes which resulted white coloured turbidity in solution (Figure S56b, schematic representation of displacement process with pictures of the H-G complex solutions). The observed white-coloured turbidity was the displaced 1-Phe-1-Nap molecules from the half cage by the 1-ENap molecules and due to its insolubility in water it separated out as precipitate. The filtered solution is coloured and its recorded ^{1}H NMR spectra is shown in green colour (1-Phe-1-Nap + Half Cage + 1-ENap), which is very similar to the ^{1}H NMR spectra of H-G complex of 1-ENap incarcerated inside Half Cage (1-ENap + Half Cage, Blue colour). From the spectra it is clear that peak from the product molecule (yellow shaded region) disappeared after addition of 1-ENap in the solution and peak corresponding to incarcerated 1-ENap (alkyne proton, light orange shaded region) molecule appeared. This confirms that reactant molecules have stronger binding constant than product molecules hence displace it from half cage during photocatalytic cycle.

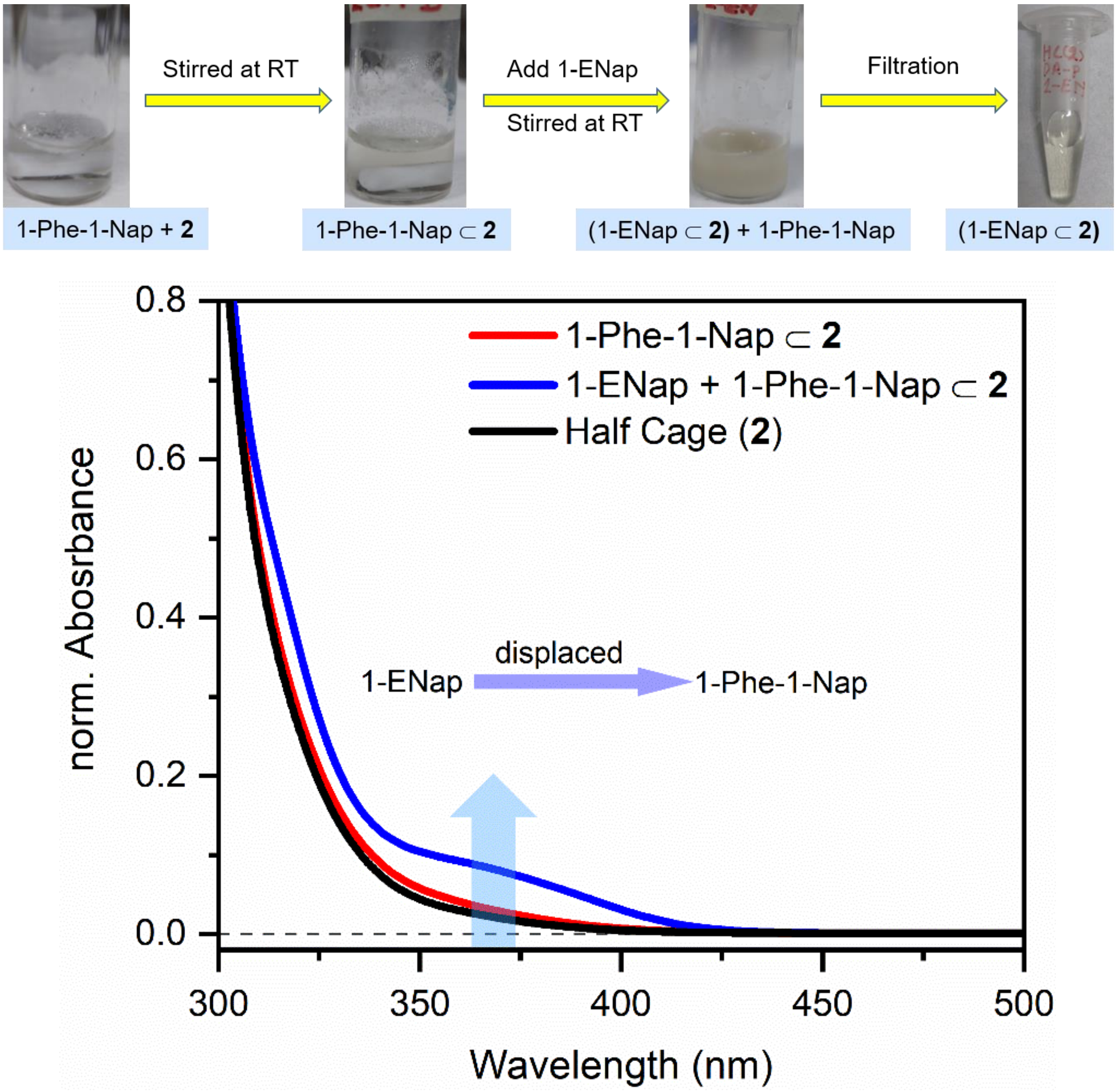

Figure S56b. Control experiment - Product displacement by reactant molecules: Displacement of cycloaddition product from half cage by the reactant molecule. The absorption spectra of 1-Phe-1-Nap incarcerated in Half Cage (red spectra) has very weak CT band as compared to the CT band present in 1-ENap incarcerated in Half Cage complex. This data also confirms that the reactant molecule can displace the product molecules during photocatalytic cycle.

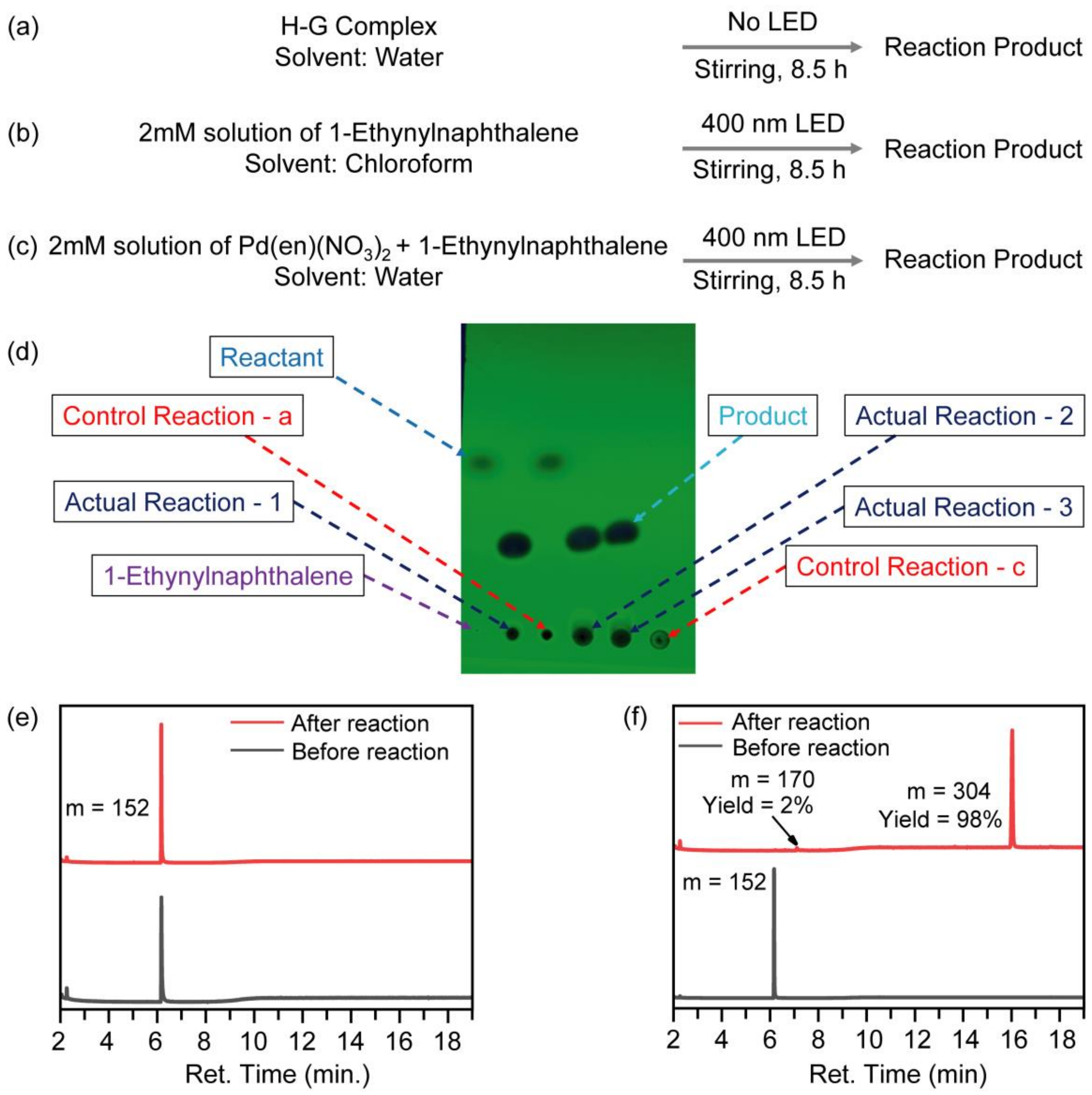

Figure S57. Control experiments in photoreaction with 1-ENap system: The three different control experiments are listed above (a), (b) & (c). The result of control experiment (a) is shown in Figure (e), where it can be seen that there is no change in chromatogram before (black spectra) and after the reaction (red spectra). The chromatograms have only peak corresponding to reactant molecule, no sign of product peak which confirms that there is no product formation in dark condition (without light; no LED). The results of control experiments (b) and (c) is summarized in fig (d) which is an image of TLC plate (TLC was run in n-hexane). The image of TLC plate clearly shows that there is no spot corresponding to cycloaddition product when reaction is done in free solution (without cavity) using chloroform as solvent. To make sure that there is no involvement of Pd-metal present in cavity photoreaction was done using metal precursor Pd(en)$(NO_3)_2$ (which is used in the synthesis of cavities) and 1-ethynylnaphthalene in aqueous medium under same reaction condition as mentioned below (in actual reaction condition). In image it can be seen that there is no spot corresponding to cycloaddition product. These two control experiments (b) and (c) confirms that there is a roll of confinement in this reaction. The actual reaction-1, 2, and 3 shows the reproducibility of this scheme. Actual reaction: H-G complex was made using host molecule (half cavity) and guest molecule (1-ethynylnaphthalene) in H_2O and 400 nm LED was used to illuminate the H-G complex. The GC-MS chromatograms of actual reaction are shown in Figure (f), reaction condition before light illumination (in black) and after light illumination (in red).

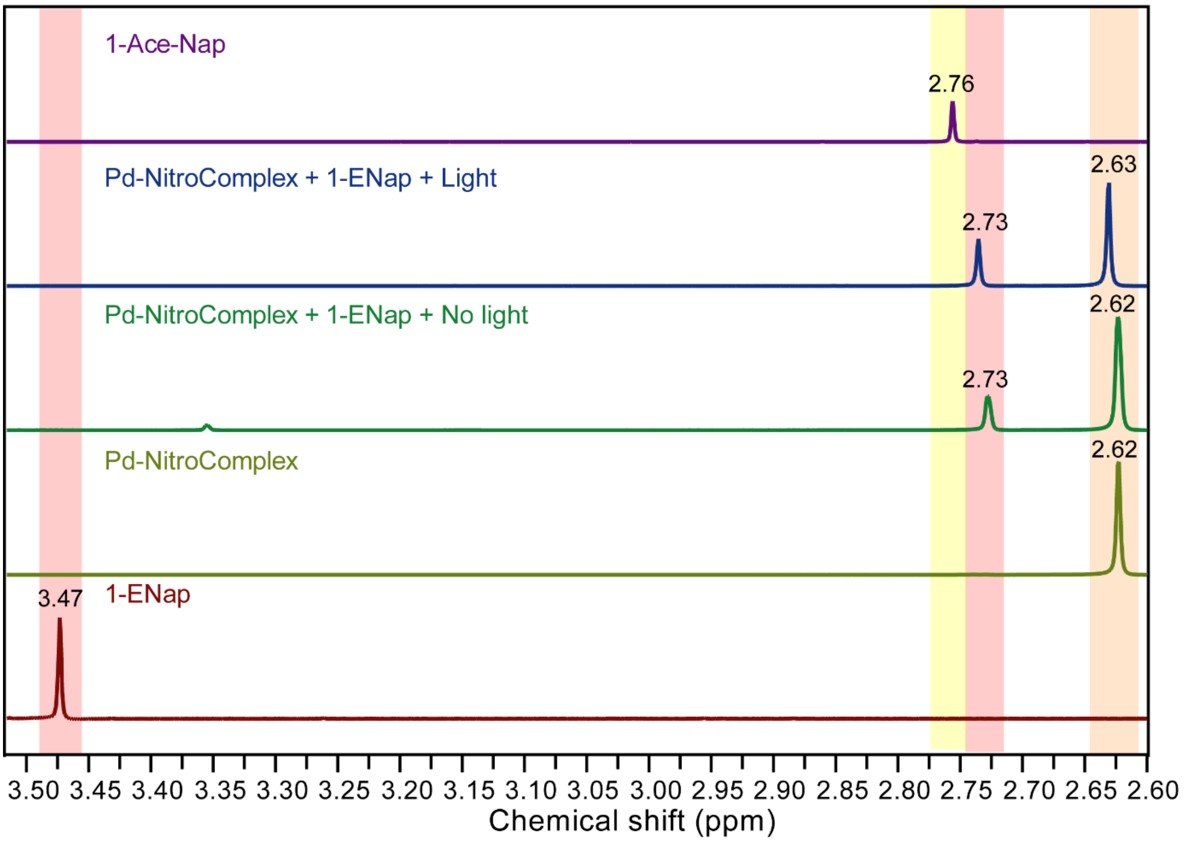

Figure S58. Control experiments in photoreaction with 1-ENap + Pd(en)(NO_3)$_2$ system: ^{1}H NMR spectra (600 MHz, Room temperature) of 1-ethynylnaphthalene (Named as: 1-ENap, $CDCl_3$), Pd(en)(NO_3)$_2$ complex (Named as: Pd-NitroComplex, D_2O), [Pd(en)(NO_3)$_2$ + 1-ENap] complex before light irradiation (Named as: PdNitroComplex + 1-ENap + No light, D_2O), [Pd(en)(NO_3)$_2$ + 1-ENap] complex after light irradiation (Named as: PdNitroComplex + 1-ENap + light, D_2O) and 1-acetonaphthone (Named as: 1-Ace-Nap, $CDCl_3$).

Control experiment: 2 equivalents of 1-ethynylnaphthalene was added in 7mM, 2mL solution of Pd(en)(NO_3) in D_2O solvent and stirred for 45 minutes which resulted a yellowish-brown coloured solution. The [Pd(en)(NO_3)$_2$ + 1-ENap] complex solution of was filtered with 2[illegible]m syringe filter. The 1mL complex solution was kept for characterisation as t_0 sample and / or reaction in dark, and remaining 1mL solution of complex was irradiated for 3.5 h using 400 nm LED which resulted a pale brownish coloured solution.

Both the reaction mixtures (before and after light irradiation) were characterized with ^{1}H NMR to check the formation of 1-acetonaphthone as oxidation product. There is shift in the peak position of alkyne proton of 1-ENap from 3.47 to 2.62 ppm after complex formation with Pd(en)(NO_3)$_2$ complex, this could be possibly due to the interaction with Pd-metal centre. The alkyne proton peak of 1-ENap before and after light irradiation is almost same at 2.73 ppm. The reaction mixture after light irradiation have only two peaks corresponding to bound alkyne protons (2.73 ppm) and ethylenediamine protons (2.63 ppm) from Pd(en)(NO_3)$_2$ complex. It does not have a peak corresponding to alkyl protons of 1-acetonaphthone which comes around 2.76 ppm (in CDCl3 solvent). This confirms that oxidation reaction does not occur in presence of Pd(en)(NO_3)$_2$ complex. The Fujita and co-workers also have mentioned (*Chem.Commun*., **2011**, 47, 10960–10962) that neither Pd-Nitro complex [Pd(tmeda)(NO_3)$_2$] nor the triazine ligand (TPT) promotes the reaction.[32]

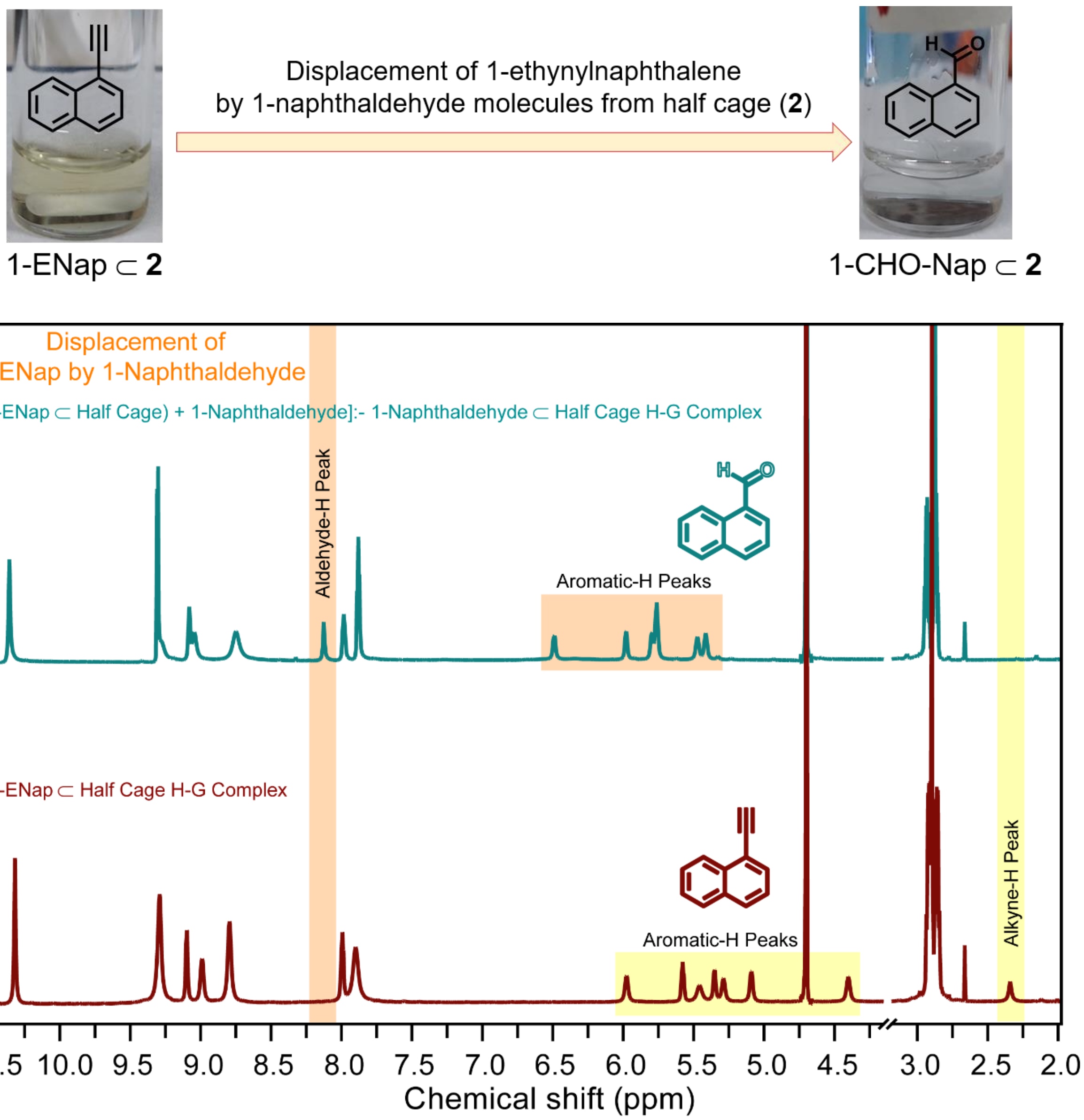

Figure S59. Control experiment - Displacement of 1-ENap by 1-naphthaldehyde molecule: Schematic representation of displacement of 1-ENap from half cage by excess 1-CHO-Nap (colour change in solution). The ^{1}H NMR spectra of 1-ENap ⊂ Half Cage H-G complex (crimson colour) and the 1H NMR spectra of resulting solution after displacement process is shown in cyan colour i.e., 1-naphthaldehyd ⊂ Half Cage H-G complex solution.

Experimental procedure: We took 1-naphthaldehyde (1-CHO-Nap) molecule having complementary naphthalene core as the reactant molecule 1-ENap. To test the displacement of 1-ENap molecule by excess 1-naphthaldehyde molecule, we added excess amount of 1-naphthaldehyde (20 equivalents with respect to half cage concentration) to the H-G complex coloured solution of 1-ENap ⊂ Half Cage and stirred for some time which resulted decolorization in the solution, indicating the H-G complexation between 1-ENap and half cage has been disturbed by excess 1-naphthaledehyde molecules, i.e. displacement of 1-ENap by 1-naphthaledehyde and formation of 1-CHO-Nap ⊂ Half Cage H-G complex. After addition of 1-CHO-Nap molecule in the H-G complex solution of 1-ENap ⊂ Half Cage there is disappearance of peaks corresponding to incarcerated 1-ethynylnaphthalene molecules (such as alkyne proton peak, highlighted with yellow shade) and appearance of peaks corresponding to incarcerated 1-naphthaledehyde molecules (such as aldehydic proton peak, highlighted in orange coloured shaded region). The peaks corresponding to the aromatic protons are also highlighted with coloured shaded region. This ^{1}H NMR data confirms that the excess amount of 1-CHO-Nap can displace the reactant molecule (1-ENap) from the half cage.

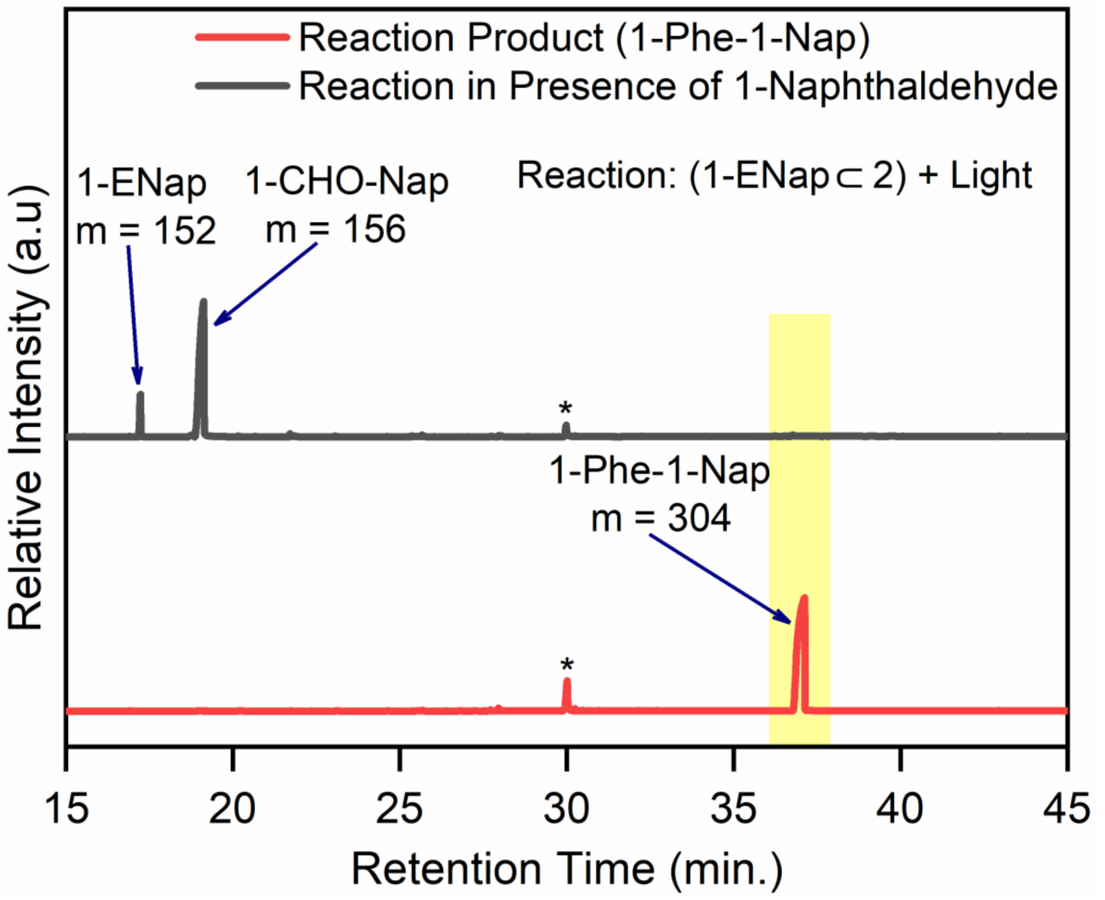

Figure S60. Control experiment - Reaction in presence of 1-naphthaldehyde: Inhibition of cavity pocket by the presence of other molecule in the reaction mixture to stop the reaction if it is driven due to the cavity pocket. The GC-MS chromatogram of the pure 1-Phe-1-Nap product molecule (red colour) and the reaction mixture obtained when the photoreaction was performed in the presence of 1-naphthaldehyde (black colour). The data shows that there is no formation of coupling product (1-Phe-1-Nap) when the reaction was done in the presence of excess amount of 1-naphthaldehyde (highlighted in the yellow region). Asterisk (*) marked peak is some impurity from the GC-MS column. The retention times of the reactant and product molecules in this chromatogram differ from those in previously recorded chromatograms (shown in Figure S22 and S26) due to the use of a different GC-MS instrument and column in this analysis.

Experimental procedure: The photoreaction was done in similar conditions by adding excess amount of 1-naphthaldehyde (20 equivalents with respect to half cage concentration) to the H-G complex solution of 1-ENap ⊂ Half Cage. The reaction mixture was analysed using GC-MS which suggested that there is no formation of cycloaddition product when reaction is done in presence of excess amount of 1-naphthaldehyde. This confirms that the [4+2] cycloaddition reaction only takes place inside the confinement of cavity not in the free solution.

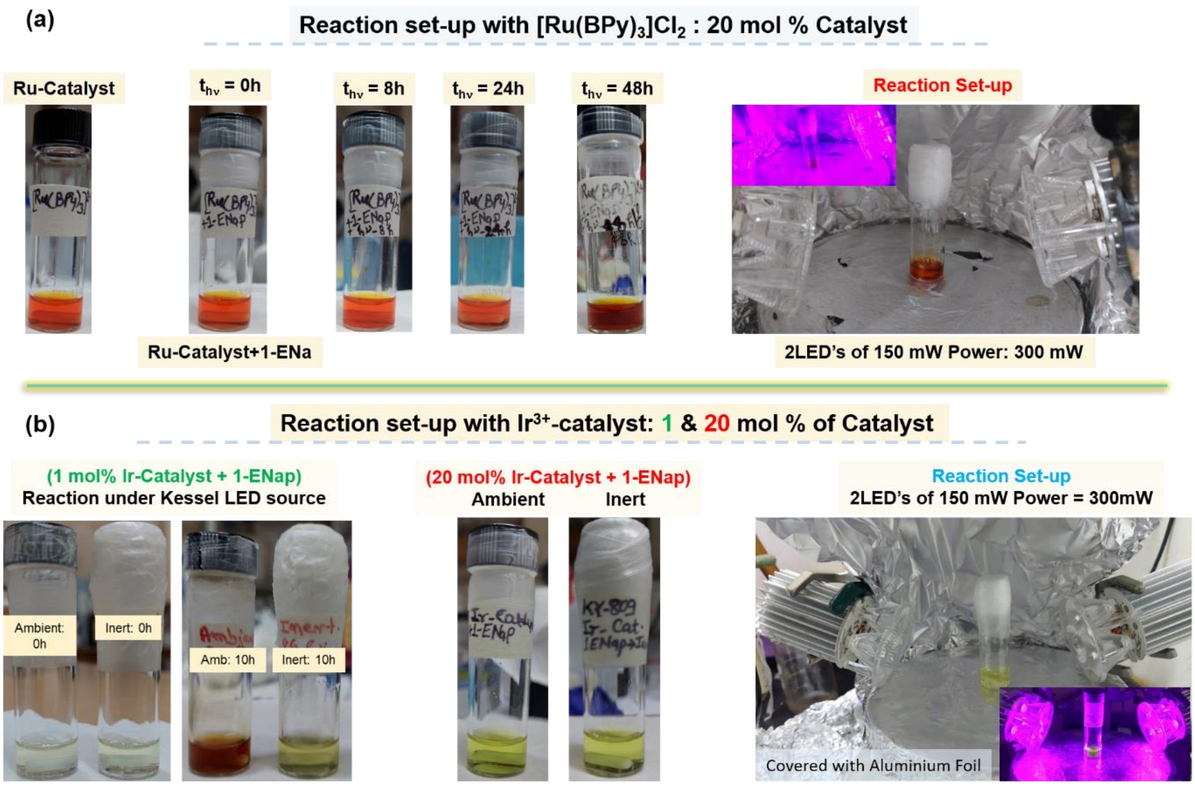

Figure S61. Reaction scheme for photoredox reactions with Ru(II)- / Ir(III) photocatalysts: (a) Reaction set-up with Ru(II)-photocatalyst and pictures of Ru(II)-catalyst (20 mol%) solution in acetonitrile, (Ru(II)-catalyst with 1-ENap) solution in acetonitrile and samples after light illumination such as t_{hv} = 0h, 8h, 24h and 48h. (b) Reaction set-up with Ir(III)-photocatalyst and images of (Ir(III)-catalyst with 1-ENap) solution in acetonitrile with 1 mol% and 20 mol% of catalyst under inert and ambient conditions. Reaction done under standard Kessel 420 nm LED light source for 10 hours using 1 mol% of Ir(III)-catalyst has a colour change in the solution as shown in figure. The 400 nm wavelength diffuse LED's of around 150 mW power was assembled to increase the total power of reaction set-up up top 300 mW.

The reaction conditions and its results are mentioned below point by point:

Cavity mediated H-G CT-driven photoreactions in water: The 5 mM H-G complex solution in water was illuminated with 400 nm diffused LED (optical power: 50 mW) light source under ambient conditions for 8.5 hours.

Conventional photoredox catalysis and its reaction conditions in organic solvents: We chose two types of well-known photoredox catalysts for the photoreactions. The chemical formulae and structures of the catalysts are as follows:

1. Ru^{2+}-metal based catalyst: [Ru(bpy)$_3$]Cl$_2$, Tris(bipyridine)ruthenium(II) chloride.
2. Ir^{3+}-metal based catalyst: [Ir{dF(CF$_3$)ppy}$_2$(dtbpy)]PF$_6$, [4,4′-Bis(1,1-dimethylethyl)-2,2′-bipyridine-N1,N1′]bis[3,5-difluoro-2-[5-(trifluoromethyl)-2-pyridinyl-N]phenyl-C]Iridium(III) hexafluorophosphate.

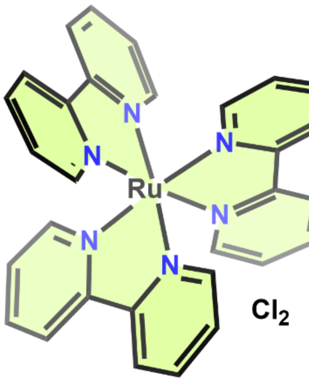

$Ru(bpy)_3]Cl_2$

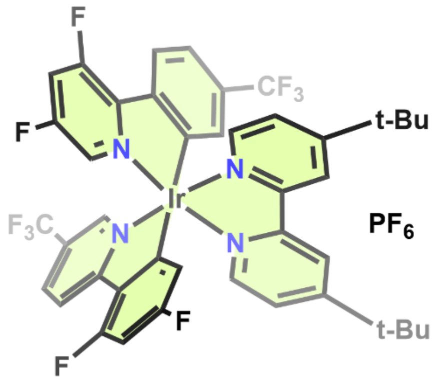

$[Ir\{dF(CF_3)ppy\}_2(dtbpy)]PF_6$

To test the feasibility and the limits of this class of non-aqueous reaction under typical photoredox conditions using well known photoredox catalysts, the reaction was performed in several conditions which are mentioned as follows:

1. **Photoredox reaction with analogous conditions as Host-Guest photocatalysis but in organic solvent:** The reaction was chosen to in acetonitrile solvent at higher concentration (20 mM) of reactant (1-ENap) using 50 mW power, 400 nm diffused LED light source with the following details:

   Reaction procedure: The 20 mM, 1 mL solution of 1-ENap in acetonitrile was taken for the reaction. The Ir(III) / Ru(II) photocatalyst was added either 1 mol% or 20 mol% and reaction was done in ambient conditions with 50 mW of 400 nm diffused LED light source. Reaction was monitored for 8 h, 24 h, 48 h and 60 h using GC-MS.

   Result: No [4+2] cycloaddition product was observed after reaction for either 1 mol% or 20 mol% of catalyst loading. There were traces of oxidation product such as 1-naphthaldehyde and 1-naphthoic acid in presence of Ru(II)-catalyst after long time (60 hours) photoirradiation of reaction mixture.

2. **Reactions with increased power of light source of 300 mW diffused LED under inert conditions:** The idea was to test the possibility of this reaction at higher power of light source. The reaction conditions and their corresponding reaction products are described below:

   (a) Reaction using 1 mol% Ir(III) catalyst: It was done using high concentration (20 mM) of reactant molecule (1-ENap) under inert and ambient conditions.

   Result: Reaction under ambient conditions yielded no coupling product. Traces of oxidation product such as 1-naphthladehyde and 1-naphthoic acid observed after 24 hours of light irradiation. However, reaction under inert conditions yielded a maximum of 3.5% of coupling product in 24 h of reaction time. The percentage of coupling product at 8.5 h was only 2.66%. This data indicates that under above mentioned conditions with 1 mol% of Ir(III)-catalyst, reaction could be pushed up to only 3.5%.

   (b) Reaction using 20 mol% catalyst: To test the limits of this reaction or to push it beyond 3.5% (observed with 1-mol% of Ir-catalyst), using photoredox catalysts in acetonitrile solvent, the reaction was performed using 20 mol% of catalyst under the assembly of LED's (300 mW optical power) in both ambient and inert conditions.

   Result: Reaction under inert conditions with Ir(III)-catalyst yielded maximum 15% of [4+2] cycloaddition product in 48 hours of reaction time while it reduced to 11% in 60 hours.

Reaction under ambient conditions with Ir(III)-catalyst yielded no [4+2] cycloaddition product. Traces of oxidation product such as 1-naphthladehyde and 1-naphthoic acid observed after long-time photoirradiation.
Reaction with Ru(II)-catalyst under inert and ambient both condition yielded only traces of oxidation product. No [4+2] cycloaddition product was observed.

These results suggest that the reaction is not economical and cannot be driven to any satisfactory level of completion in bulk organic medium even with higher mol% (20 mol%) of photoredox catalyst along with high concentration (20 mM) of reactant.

(c) **Reaction with standard Kessel 420 nm LED light source (40W), typical for Organic photoredox catalysis:** The reaction was done using typical standard photoredox conditions with 1 mol% of Ir(III)-catalyst with high concentration (20 mM) of 1-ENap under inert and ambient conditions.
Result: Reaction under ambient conditions yielded no [4+2] cycloaddition product. There are traces of oxidation product such as 1-naphthaldehyde. However, the reaction under inert conditions yielded only 8.5% of [4+2] cycloaddition product in 10 hours of reaction time.

All the above data cumulatively suggest that at high concentrations of substrates and prolonged hours of high intensity light illumination, small amount of [4+2] cycloaddition product can be obtained under inert conditions. However, this method clearly cannot be translated for driving complete conversion as the catalyst or products degrade in bulk organic solvents in 60 hours.

Our host-guest photocatalytic method in contrast clearly provides 100% conversion with 98% selectivity in just 8.5 hours at much lesser light intensities in water, with the only requirement of a geometrically suitable nanocage.

The GC-MS chromatograms related to few of the above-mentioned experiments with Ru(II)- and Ir(III)-photoredox catalysts and higher concentration of reactant (1-ENap) molecules are shown in Figure S62:

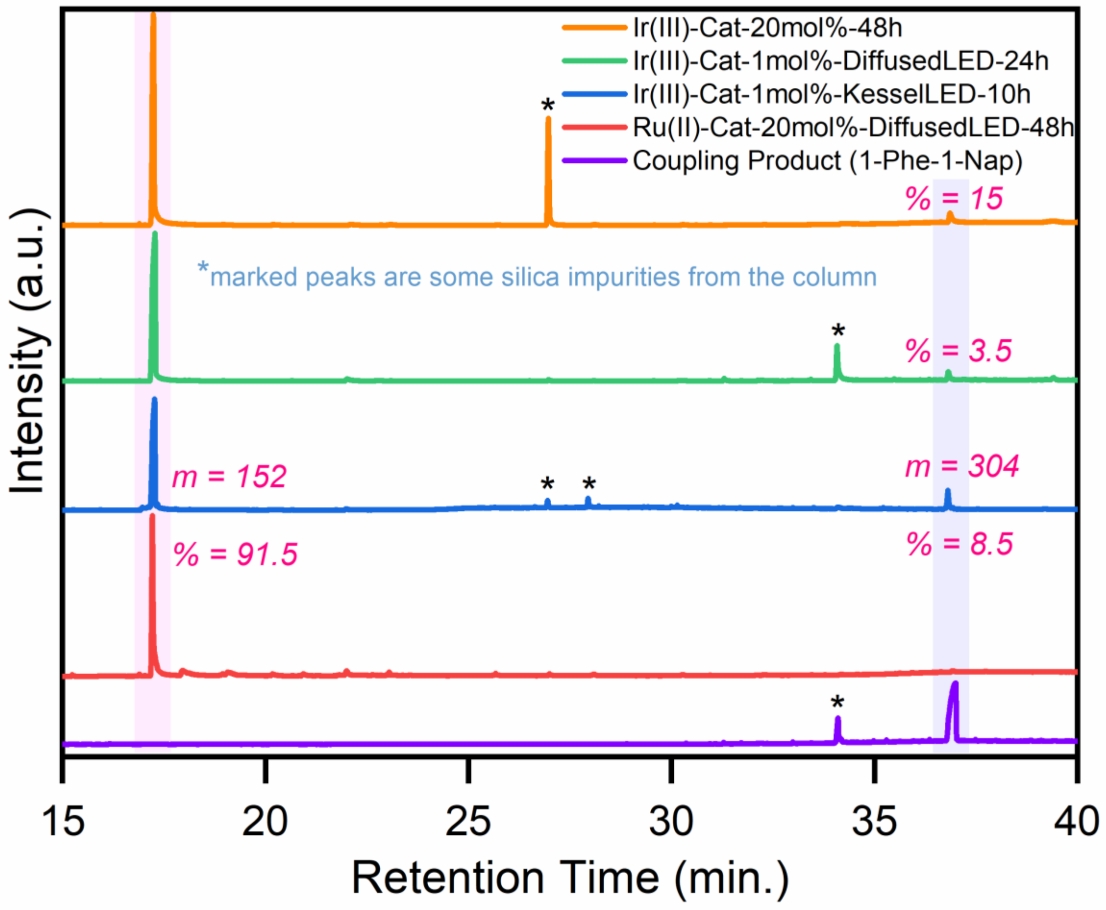

Figure S62. GC-MS chromatograms for reactions with Ru(II)- / Ir(III) photocatalysts: GC-MS chromatograms of the experiments done with Ru(II)- and Ir(III)-photoredox catalysts under different reaction conditions. The [4+2] cycloaddition product (1-Phe-1-Nap) was run in the GC-MS instrument separately and its chromatogram is shown in spectra (bottom). From the datasets shown above, it is clear that there is nominal conversion to coupling product by this conventional photoredox way. The major peak around retention time of 17 minutes correspond to the starting material (1-ENap) and the peak around 37 minutes correspond to the [4+2] cycloaddition product (1-Phe-1-Nap). There are some additional peaks in the chromatograms marked with asterisks (*), these are from the silica impurities coming from the GCMS column. By this method we could push reaction to only 15% in 48 h using higher power of light source and higher mole percent (20 mol %) of Ir(III)-catalyst under inert reaction condition.

All the results mentioned above suggests that this [4+2] cycloaddition reaction is not favourable in bulk medium using traditional Ru(II)- / Ir(III)-based photoredox catalysts. Now it's clear that cavity-based approach, accelerates the reaction rate and make it more selective via pre-polarization and preorganization of bonds. Importantly we could do this reaction in water under ambient conditions using significantly low power LED light source, which is not a very easy task via traditional ways of organic transformation because most of the organic transformations occur in organic medium using very high-power LED light sources.

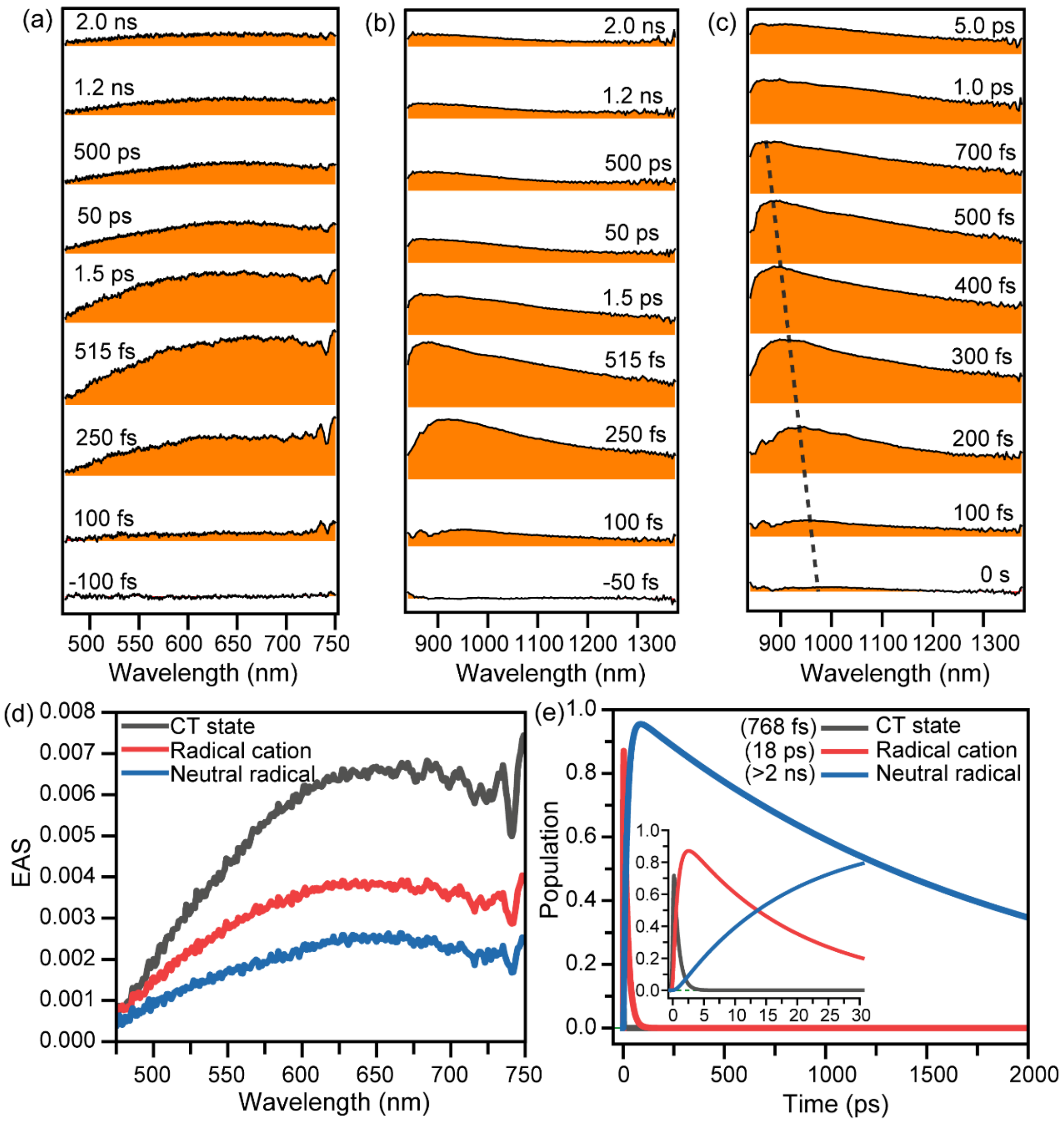

Figure S63. Transient absorption spectra of 1-ENap ⊂ 2 H-G complex: Transient absorption (TA) data of 1-ENap ⊂ Half cage H-G CT complex: (a) TA spectra in the visible probe window, (b) NIR probe window. TA spectra show the increase in the population of excited state absorption (ESA) spectra first, then it started to decrease with non-decaying population up to 2 ns. (c) The excited state absorption spectra at different time delays after photoexcitation in charge transfer band region. The blue shift in the ESA spectra (black dotted line) up to around 1ps time delay clearly shows the relaxation of CT state in the excited state through the solvation dynamic process. (d) The ESA spectra of species associated after photoexcitation on H-G CT complex. The photoexcitation led to the generation of radical cation (red spectra) at guest molecule and radical anion on half-cage simultaneously.[6; 30] The feature in near IR region arises from the host-guest CT interaction and the radical anion state of cavity (after solvation dynamics time scale).[6; 31] The radical cation interacts with water molecules in the vicinity, give its proton to water molecules and generate neutral radical species (blue spectra) which have life time of more than 2 ns. The respective decay kinetics of the species associated in this process is shown in (e).

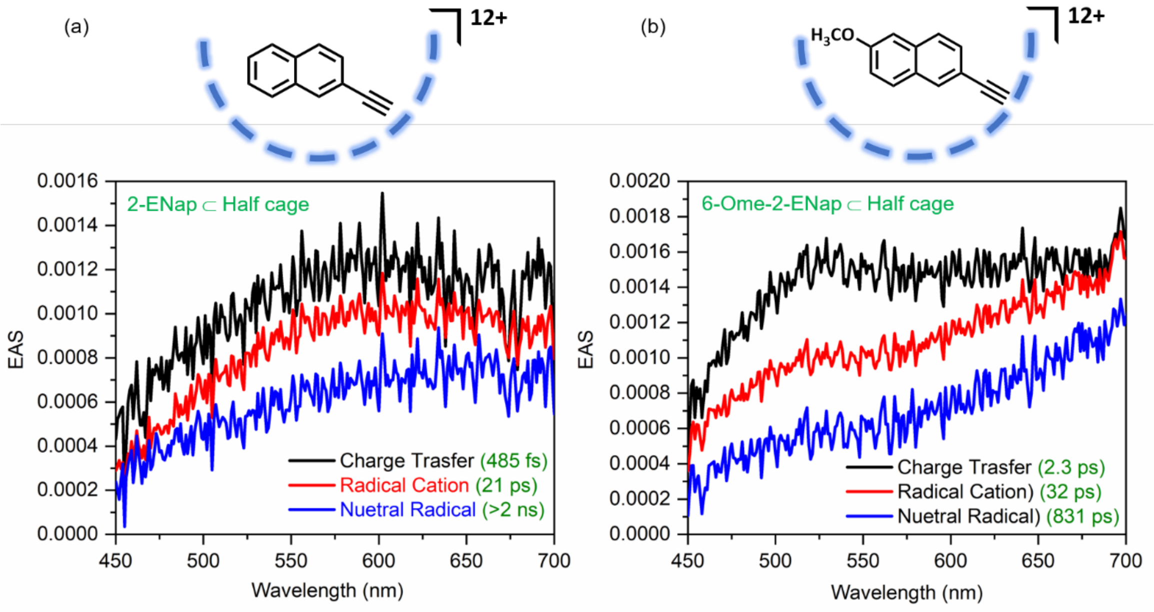

Figure S64. Transient absorption spectra of 2-ENap ⊂ 2 and 6-Ome-2-ENap ⊂ 2: Transient absorption data of 2-ENap ⊂ Half Cage and 6-Ome-2-ENap ⊂ Half cage H-G CT complex: (a) Species associated spectra after photoexcitation (EAS) of 2-ENap ⊂ Half Cage H-G complex system with their respective life time, charge transfer state (black) have life-time of 485 fs, radical cation (red) state have life-time of 21 ps and neutral radical (blue) states have life-time of more than 2 ns. Similarly, the EAS spectra and their respective life of 6-Ome-2-ENap ⊂ Half Cage H-G CT complex is shown in figure (b).

The photoexcitation in CT band region led to the generation of radical cation (red spectra) at guest molecule and radical anion on cage simultaneously. The radical cation interacts with water molecules in the vicinity, give its proton to water molecules and generate neutral radical species (blue spectra). From the data in figure (a) and (b), it is clear that the deprotonation step is slow in case of 6-Ome-2-ENap ⊂ Half Cage and this could be due to the electron donating nature of methoxy group which pushed the electron density of the naphthalene ring making it electron rich. And ultimately this lower down the acidity of radical cation formed on 6-Ome-2-ENap as compared to the radical cation on 2-ENap that's why the deprotonation process is slow in later case. Not only this the methoxy group also altered the life time of neutral radical formed after deprotonation step. In case of 2-ENap ⊂ Half Cage the neutral radical has life time of more than 2 ns while in case of 6-Ome-2-ENap ⊂ Half Cage the life time is just 830 ps. This shows that the methoxy group does not only makes the naphthalene ring sufficiently electron rich and lowers the acidity of radical cation but also alters the packing of guest molecules inside half cage. In this case there is possibility that the radical cation is exposed to nearby water molecules but due to its lower acidity the deprotonation step is not that much favourable so the nucleophilic attack of water molecule can take place on radical cation and leads the formation of oxidation product. This could be one reason that in case of methoxy substituted derivative of 2-ENap i.e. 6-Ome-2-ENap the oxidation product is major one and in case of 2-ENap the coupling product is the major product.

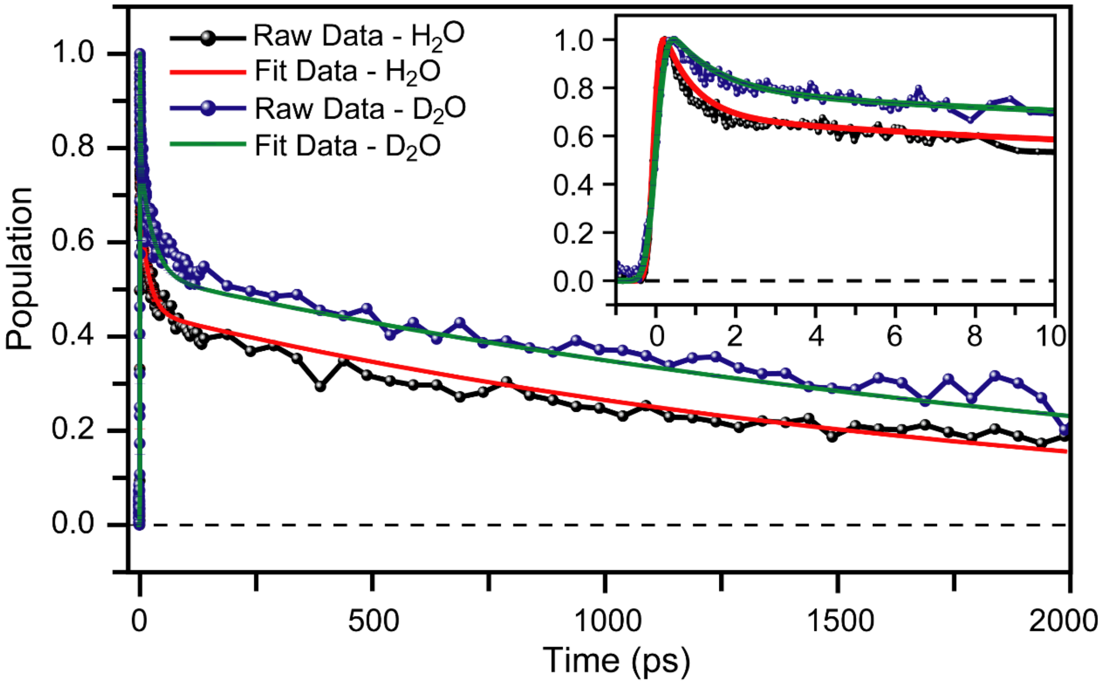

Figure S65. Comparative study of decay kinetics of the radical cation state in H_2O & D_2O: Secondary kinetic isotope effect: comparative decay kinetics of the radical cation state to form neutral radical in H_2O versus D_2O solvents. The decay rate of radical cation state is slow in D_2O as compared to H_2O. The decay kinetic trace at initial time points is shown in inset. The difference in rate of the decay kinetics suggest that there is involvement of proton removal step in this process of neutral radical generation.[6]

Chemical formula	m/z	Observed m/z
$C_{24}H_{15}D$	305.1307	305.1307

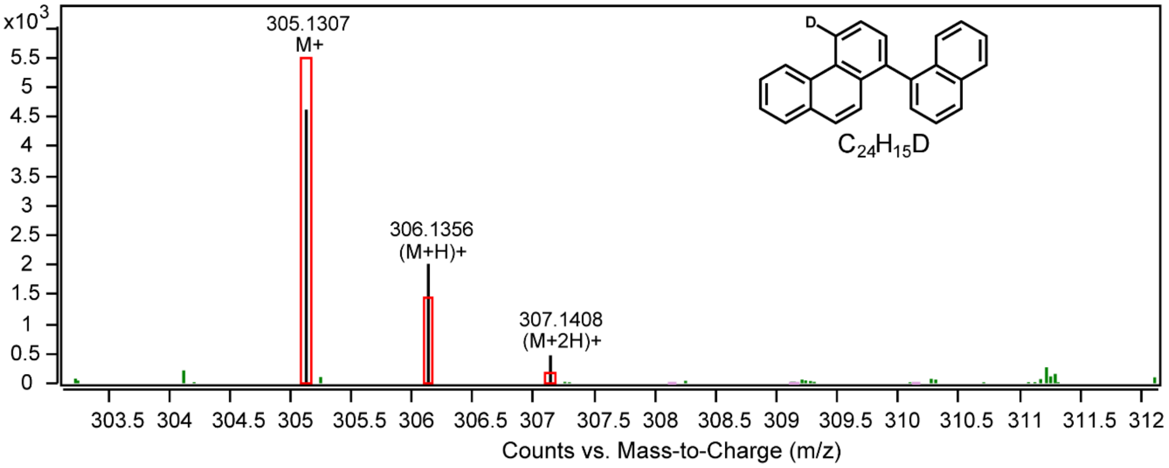

Figure S66. HR-MS chromatogram of cycloaddition product after photoreaction in D_2O: The HR-MS data after reaction done in D_2O solvent to track the back electron and proton transfer process. In this case expected chemical formula and molecular weight are $C_{24}H_{15}D$ and 305.1307 Da. The chromatogram has parent mass (M+) with highest intensity along with coupling adducts with hydrogen. The Calculated m/z (305.1307 Da) and observed m/z (305.1307 Da) have excellent match up to four decimal places. The HR-MS data highly supports to the formation of $C_{24}H_{15}D$ product, which confirms that back proton transfer process is taking place from solvent molecules during catalytic process.

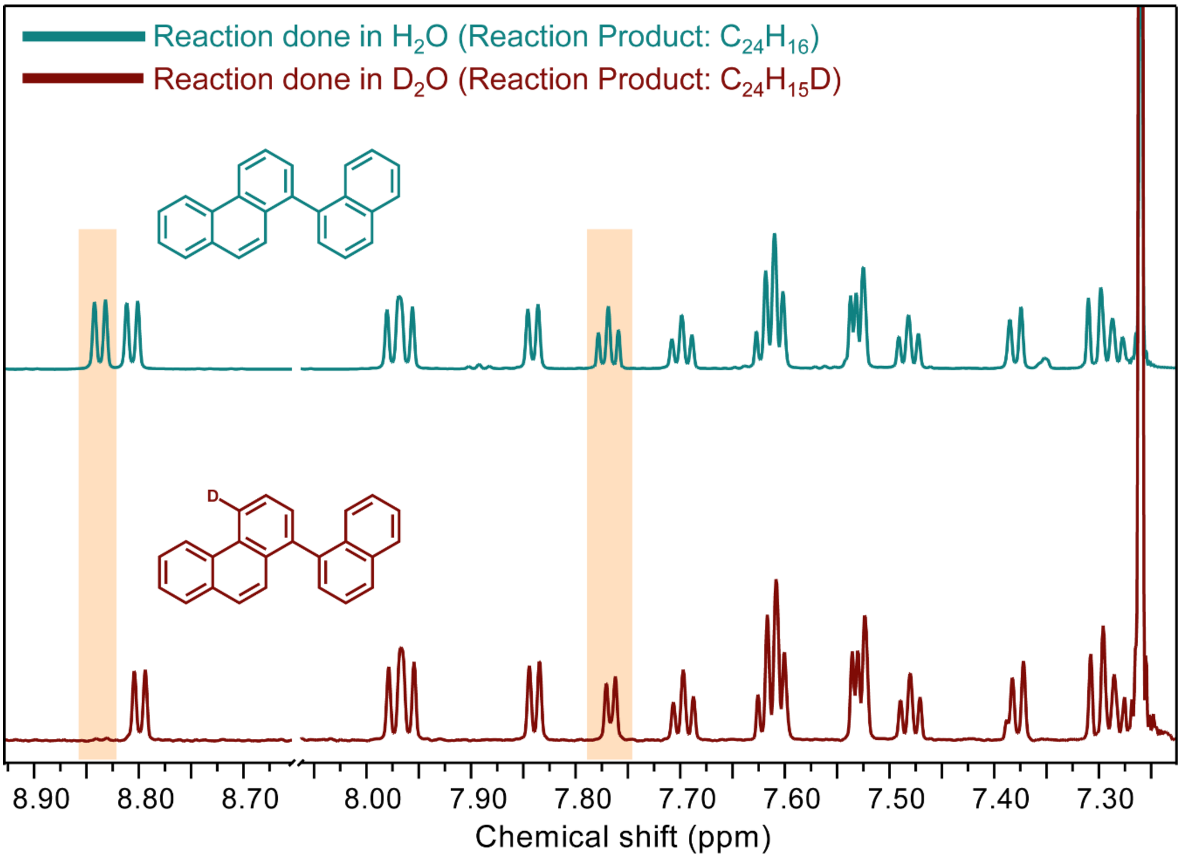

Figure S67. ^{1}H NMR spectra of cycloaddition product after photoreaction in D_2O: ^{1}H NMR spectra in aromatic region (800 MHz, $CDCl_3$) of photoproducts, when reaction is done in H_2O (green spectra; top; $C_{24}H_{16}$) and when reaction is done in D_2O (red spectra; bottom; $C_{24}H_{15}D$). Comparison of these spectra clearly shows that there is disappearance of one doublet peak (8.84 ppm) and conversion of one triplet peak to a doublet peak (7.77 ppm) highlighted with orange shaded region. The complete disappearance of doublet (8.84 ppm) and formation of one doublet at the place of triplet peak (7.77 ppm) clearly break the pattern of one d-t-d coupling scheme which is present in $C_{24}H_{16}$ molecule (clear coupling patterns are shown in Figure S35). This confirms the addition of deuterium atom during reaction and formation of coupling product $C_{24}H_{15}D$, which proves that there is involvement of back electron and proton transfer step during complete catalytic cycle.

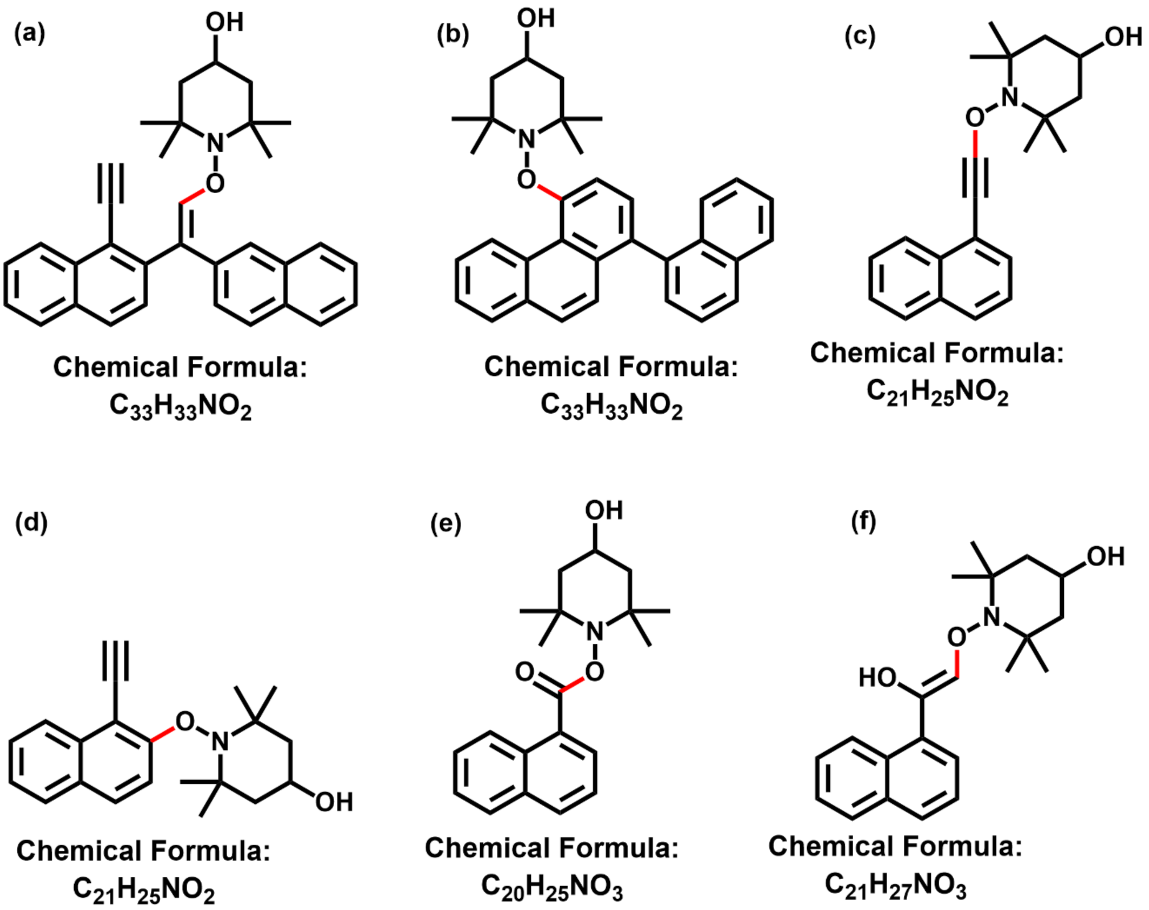

Figure S68a. All the potential adducts of TEMPO based radical trapping: The structure (b), (e) and (f) are observed in HRMS data.

In the above shown structures, (a) and (b) have same chemical formula so the molecular weight, and similarly structure (c) and (d).

Reaction Condition: The excess amount (15 equivalents with respect to cage concentration) of 4-hydoxy-TEMPO was added in the H-G Complex solution of 1-ENap ⊂ Half Cage and photoreaction was done in inert condition. The reaction mixture was analyzed using HR-MS, we could see only the masses corresponding to the chemical formula $C_{33}H_{33}NO_2$ [(structures (a) or (b)], $C_{20}H_{25}NO_3$ [structure (e)] and $C_{21}H_{27}NO_3$ [structure (f)] while no masses corresponding to the chemical formula structures (c) or (d) were observed. This indicates that the radical corresponding to either structure (c) or (d) are instantaneously reacting with neighbouring molecule due to proximity induced reactivity just after their formation. We think this could a possible reason for those not being trapped by 4-hydroxy-TEMPO.

The structure corresponding to chemical formula $C_{33}H_{33}NO_2$ is (b) since we also observed the deuterium atom addition at the same position when reaction was done in D_2O solvent. This data showed that only the radical after the cyclization step could be trapped with 4-hydroxy-TEMPO. No other potential intermediate radical species that could be involved in the step-wise cyclization pathways were trapped during the photoreaction. Another important observation is that the reaction product obtained in this reaction is the result of cyclization reaction going through less stable radical after the addition of photogenerated radical to the adjacent neutral molecule. These datasets and observations confirmed that the cyclization reaction in this case is a concerted mechanism.

Molecular (M) Formula	m/z	Observed m/z
M = $C_{33}H_{33}NO_2$	475.2441	475.2441
M + H = $C_{33}H_{34}NO_2$	476.2531	476.2531

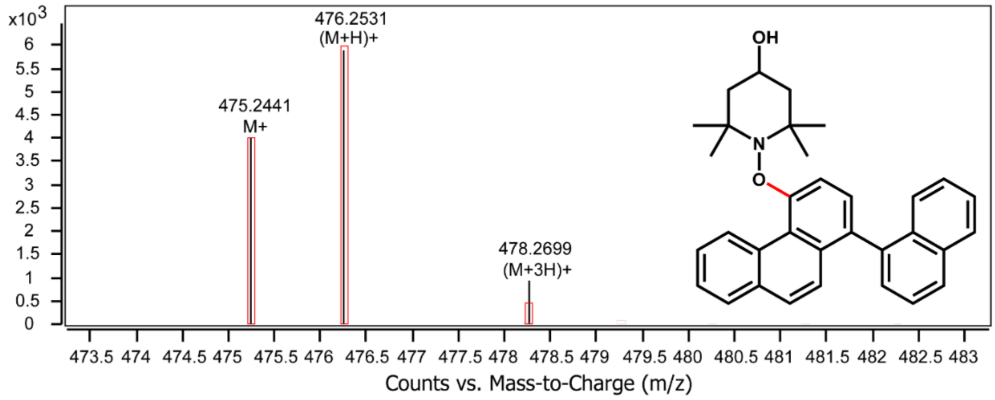

Molecular (M) Formula	m/z	Observed m/z
M = $C_{20}H_{25}NO_3$	327.1753	327.1753
M + H = $C_{20}H_{26}NO_3$	328.1902	328.1902

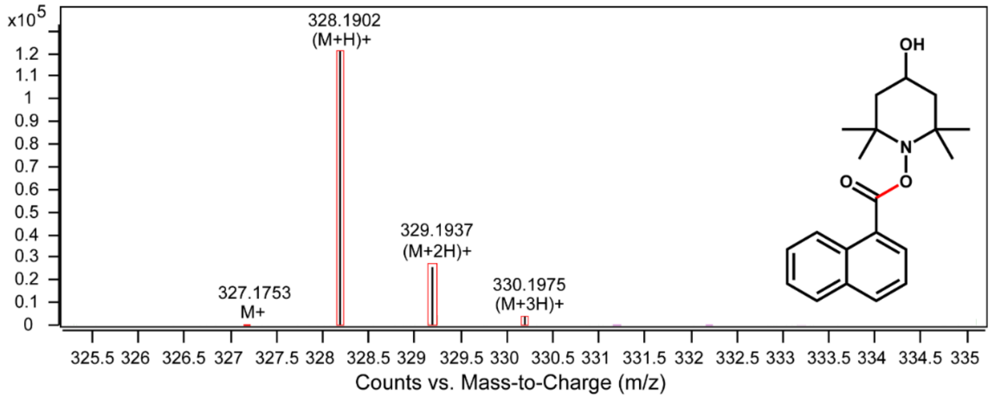

Molecular (M) Formula	Theoretical m/z	Observed m/z
(M+H) = $C_{21}H_{28}NO_3$	342.2060	342.2060

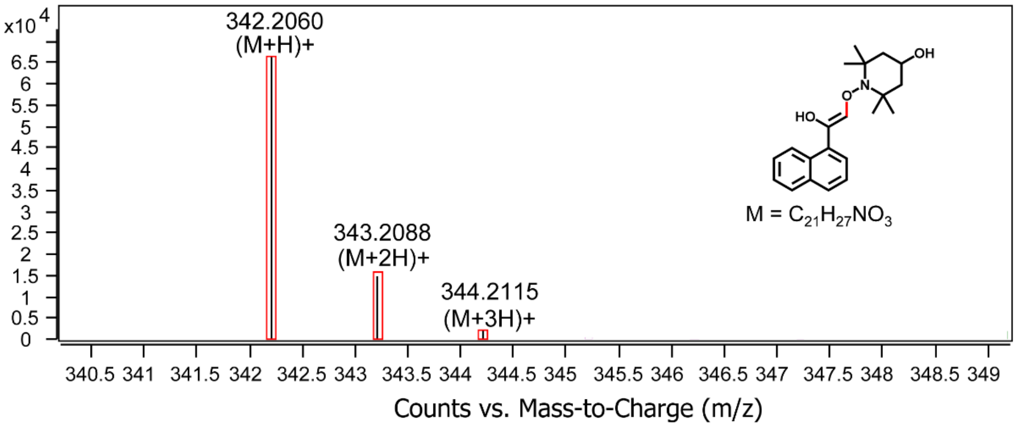

Figure S68b. HR-MS chromatograms of radical intermediates trapped with 4-hydroxy-TEMPO: The HR-MS data and structures of the species trapped with 4-hydroxy-TEMPO. The structure of the radical species formed after the cyclization step and trapped with 4-hydroxy-TEMPO is shown in top figure. The radical species generated after the water attack to the photogenerated radical cation during alkyne hydration reaction is shown in bottom figure after trapping with 4-hydroxy-TEMPO.

REFERENCES;

1. Gilg, K., Mayer, T., Ghaschghaie, N., and Klüfers, P. (2009). The metal-binding sites of glycose phosphates. Dalton Transactions, 7934-7945.
2. Zhu, L., Lu, M., Qu, D., Wang, Q., and Tian, H. (2011). Coordination-assembly for quantitative construction of bis-branched molecular shuttles. Organic & Biomolecular Chemistry *9*, 4226-4233.
3. Leenders, S.H., Becker, R., Kumpulainen, T., de Bruin, B., Sawada, T., Kato, T., Fujita, M., and Reek, J.N. (2016). Selective Co-Encapsulation Inside an M6L4 Cage. Chemistry–A European Journal *22*, 15468-15474.
4. Fujita, M., Yu, S.Y., Kusukawa, T., Funaki, H., Ogura, K., and Yamaguchi, K. (1998). Self-assembly of nanometer-sized macrotricyclic complexes from ten small component molecules. Angewandte Chemie International Edition *37*, 2082-2085.
5. Fujita, M., Oguro, D., Miyazawa, M., Oka, H., Yamaguchi, K., and Ogura, K. (1995). Self-assembly of ten molecules into nanometre-sized organic host frameworks. Nature *378*, 469-471.
6. Das, A., Mandal, I., Venkatramani, R., and Dasgupta, J. (2019). Science advances *5*, eaav4806.
7. Snellenburg, J.J., Laptenok, S., Seger, R., Mullen, K.M., and van Stokkum, I.H.M. (2012). Glotaran: A Java-Based Graphical User Interface for the R Package TIMP. Journal of Statistical Software *49*, 1 - 22.
8. Neese, F., Wennmohs, F., Becker, U., and Riplinger, C. (2020). The ORCA quantum chemistry program package. The Journal of Chemical Physics *152*.
9. Pracht, P., Bohle, F., and Grimme, S. (2020). Automated exploration of the low-energy chemical space with fast quantum chemical methods. Physical Chemistry Chemical Physics *22*, 7169-7192.
10. Grimme, S. (2019). Exploration of Chemical Compound, Conformer, and Reaction Space with Meta-Dynamics Simulations Based on Tight-Binding Quantum Chemical Calculations. Journal of Chemical Theory and Computation *15*, 2847-2862.
11. Grimme, S., Bohle, F., Hansen, A., Pracht, P., Spicher, S., and Stahn, M. (2021). Efficient Quantum Chemical Calculation of Structure Ensembles and Free Energies for Nonrigid Molecules. The Journal of Physical Chemistry A *125*, 4039-4054.
12. Becke, A.D. (1997). Density-functional thermochemistry. V. Systematic optimization of exchange-correlation functionals. The Journal of Chemical Physics *107*, 8554-8560.
13. Grimme, S., Ehrlich, S., and Goerigk, L. (2011). Effect of the damping function in dispersion corrected density functional theory. Journal of Computational Chemistry *32*, 1456-1465.
14. Grimme, S. (2006). Semiempirical GGA-type density functional constructed with a long-range dispersion correction. Journal of Computational Chemistry *27*, 1787-1799.
15. Adamo, C., and Barone, V. (1999). Toward reliable density functional methods without adjustable parameters: The PBE0 model. The Journal of Chemical Physics *110*, 6158-6170.
16. Caldeweyher, E., Bannwarth, C., and Grimme, S. (2017). Extension of the D3 dispersion coefficient model. The Journal of Chemical Physics *147*.
17. Weigend, F., and Ahlrichs, R. (2005). Balanced basis sets of split valence, triple zeta valence and quadruple zeta valence quality for H to Rn: Design and assessment of accuracy. Physical Chemistry Chemical Physics *7*, 3297-3305.
18. Marenich, A.V., Cramer, C.J., and Truhlar, D.G. (2009). Universal Solvation Model Based on Solute Electron Density and on a Continuum Model of the Solvent Defined by the Bulk Dielectric Constant and Atomic Surface Tensions. The Journal of Physical Chemistry B *113*, 6378-6396.
19. . Chemcraft - graphical software for visualization of quantum chemistry computations.
20. Macrae, C.F., Sovago, I., Cottrell, S.J., Galek, P.T.A., McCabe, P., Pidcock, E., Platings, M., Shields, G.P., Stevens, J.S., Towler, M., *et al.* (2020). Mercury 4.0: from visualization to analysis, design and prediction. Journal of Applied Crystallography *53*, 226-235.
21. Tagliatesta, P., Elakkari, E., Leoni, A., Lembo, A., and Cicero, D. (2008). Highly selective biaryl formation by the cyclooligomerization of arylethynes catalyzed by rhodium and ruthenium porphyrins. New Journal of Chemistry *32*, 1847-1849.
22. Diao, H., Wang, C., Zhang, Z., Shi, Z., and Liu, F. (2021). Fe-Catalyzed Intramolecular Cross-Dehydrogenative Arylation (CDA), Efficient Synthesis of 1-Arylnaphthalenes and 4-Arylcoumarins. Helvetica Chimica Acta *104*, e2100056.
23. Li, Y., and Fleming, F.F. (2016). Direct Conversion of Nitriles into Alkene “Isonitriles”. Angewandte Chemie International Edition *55*, 14770-14773.
24. Gryff-Keller, A. (2011). Theoretical modeling of 13C NMR chemical shifts—How to use the calculation results. Concepts in Magnetic Resonance Part A *38A*, 289-307.

25. Bagno, A., Rastrelli, F., and Saielli, G. (2006). Toward the Complete Prediction of the 1H and 13C NMR Spectra of Complex Organic Molecules by DFT Methods: Application to Natural Substances. Chemistry – A European Journal *12*, 5514-5525.
26. Iron, M.A. (2017). Evaluation of the Factors Impacting the Accuracy of 13C NMR Chemical Shift Predictions using Density Functional Theory—The Advantage of Long-Range Corrected Functionals. Journal of Chemical Theory and Computation *13*, 5798-5819.
27. Pierens, G.K. (2014). 1H and 13C NMR scaling factors for the calculation of chemical shifts in commonly used solvents using density functional theory. Journal of Computational Chemistry *35*, 1388-1394.
28. Toomsalu, E., and Burk, P. (2015). Critical test of some computational methods for prediction of NMR 1H and 13C chemical shifts. Journal of Molecular Modeling *21*, 244.
29. Grimme, S., Bannwarth, C., and Shushkov, P. (2017). A Robust and Accurate Tight-Binding Quantum Chemical Method for Structures, Vibrational Frequencies, and Noncovalent Interactions of Large Molecular Systems Parametrized for All spd-Block Elements (Z = 1–86). Journal of Chemical Theory and Computation *13*, 1989-2009.
30. Roy, D., Paul, S., and Dasgupta, J. (2023). Photocatalytic terminal C− C coupling reaction inside water soluble nanocages. Angewandte Chemie International Edition *62*, e202312500.
31. Furutani, Y., Kandori, H., Kawano, M., Nakabayashi, K., Yoshizawa, M., and Fujita, M. (2009). In Situ Spectroscopic, Electrochemical, and Theoretical Studies of the Photoinduced Host−Guest Electron Transfer that Precedes Unusual Host-Mediated Alkane Photooxidation. Journal of the American Chemical Society *131*, 4764-4768.
32. Murase, T., Takezawa, H., and Fujita, M. (2011). Photo-driven anti-Markovnikov alkyne hydration in self-assembled hollow complexes. Chemical Communications *47*, 10960-10962.
